# A modular chemigenetic calcium indicator for multiplexed in vivo functional imaging

**DOI:** 10.1038/s41592-024-02411-6

**Published:** 2024-09-20

**Authors:** Helen Farrants, Yichun Shuai, William C. Lemon, Christian Monroy Hernandez, Deng Zhang, Shang Yang, Ronak Patel, Guanda Qiao, Michelle S. Frei, Sarah E. Plutkis, Jonathan B. Grimm, Timothy L. Hanson, Filip Tomaska, Glenn C. Turner, Carsen Stringer, Philipp J. Keller, Abraham G. Beyene, Yao Chen, Yajie Liang, Luke D. Lavis, Eric R. Schreiter

**Affiliations:** 1 Janelia Research Campus, Howard Hughes Medical Institute, Ashburn, VA USA; 2 Department of Neuroscience, Washington University in St. Louis, St. Louis, MO USA; 3 Department of Diagnostic Radiology and Nuclear Medicine, University of Maryland School of Medicine, Baltimore, MD USA; 4 Department of Chemical Biology, Max Planck Institute for Medical Research, Heidelberg, Germany; 5 Present Address: Department of Pharmacology, University of California San Diego, La Jolla, CA USA; 6 Present Address: Department of Electrical and Computer Engineering, Center for BioEngineering, Neuroscience Research Institute, University of California, Santa Barbara, Santa Barbara, CA USA

**Keywords:** Fluorescent dyes, Fluorescent proteins, Imaging, Neuroscience

## Abstract

Genetically encoded fluorescent calcium indicators allow cellular-resolution recording of physiology. However, bright, genetically targetable indicators that can be multiplexed with existing tools in vivo are needed for simultaneous imaging of multiple signals. Here we describe WHaloCaMP, a modular chemigenetic calcium indicator built from bright dye-ligands and protein sensor domains. Fluorescence change in WHaloCaMP results from reversible quenching of the bound dye via a strategically placed tryptophan. WHaloCaMP is compatible with rhodamine dye-ligands that fluoresce from green to near-infrared, including several that efficiently label the brain in animals. When bound to a near-infrared dye-ligand, WHaloCaMP shows a 7× increase in fluorescence intensity and a 2.1-ns increase in fluorescence lifetime upon calcium binding. We use WHaloCaMP1a to image Ca^2+^ responses in vivo in flies and mice, to perform three-color multiplexed functional imaging of hundreds of neurons and astrocytes in zebrafish larvae and to quantify Ca^2+^ concentration using fluorescence lifetime imaging microscopy (FLIM).

## Abstract (web summary)

WHaloCaMP is a chemigenetic calcium indicator that can be combined with different rhodamine dyes for multiplexed or FLIM imaging in vivo, as demonstrated for calcium imaging in neuronal cultures, brain slices, *Drosophila*, zebrafish larvae and the mouse brain.

## Main

Fluorescent indicators allow non-invasive imaging of cellular physiology in living animals, and genetically encoded fluorescent Ca^2+^ indicators (GECIs) have been especially useful to approximate neuronal activity during behavior^1–3^. The most widely used GECIs are engineered from fluorescent proteins and emit in the green-to-red region of the visible spectrum. Near-infrared (~670–900 nm) fluorophores can be imaged deeper in tissue due to reduced scattering and multiplexed with existing green and red probes^4^. However, there are limited functional indicators in the near-infrared.

Existing near-infrared fluorescent indicators require either a cofactor or a synthetic small molecule, which poses challenges for in vivo use^5^. Near-infrared Ca^2+^ indicators that make use of biliverdin-binding proteins^6,7^ are limited by the availability of biliverdin, a product of heme catabolism in mammalian cells, and have low quantum yields, making in vivo imaging with good signal-to-noise ratio challenging. While synthetic small-molecule dyes can be brighter than biliverdin-binding proteins, they must be delivered to the desired tissue. In addition, to achieve genetic targeting, the small-molecule dyes must be selectively retained by a specific cell type, for example, by using a self-labeling protein^8^. We previously engineered fluorescent indicators^9^ with far-red or near-infrared emission using environmentally sensitive dye-ligands, such as JF_635_-HaloTag ligand^10^ and the self-labeling HaloTag protein^9^ in a ‘chemigenetic’ or ‘hybrid’ approach. However, these dye-ligands exhibited limited bioavailability in the central nervous system and the resulting sensors could not be efficiently used for functional imaging in the brains of animals.

Here, we describe chemigenetic Ca^2+^ indicators built from bright dye-ligands for which the fluorescence output is modulated by photoinduced electron transfer (PET) to a protein-based quencher. The modular approach allowed us to use rhodamine dye-ligands with emission ranging from green to near-infrared, including dye-ligands that efficiently distribute into the central nervous system in animals. We use these sensors to demonstrate imaging of Ca^2+^ responses in flies and mice and perform three-color multiplexed functional imaging as well as quantitative Ca^2+^ FLIM in zebrafish larvae.

## Results

### Design of the chemigenetic Ca^2+^ indicator WHaloCaMP

**Fig. 1:**
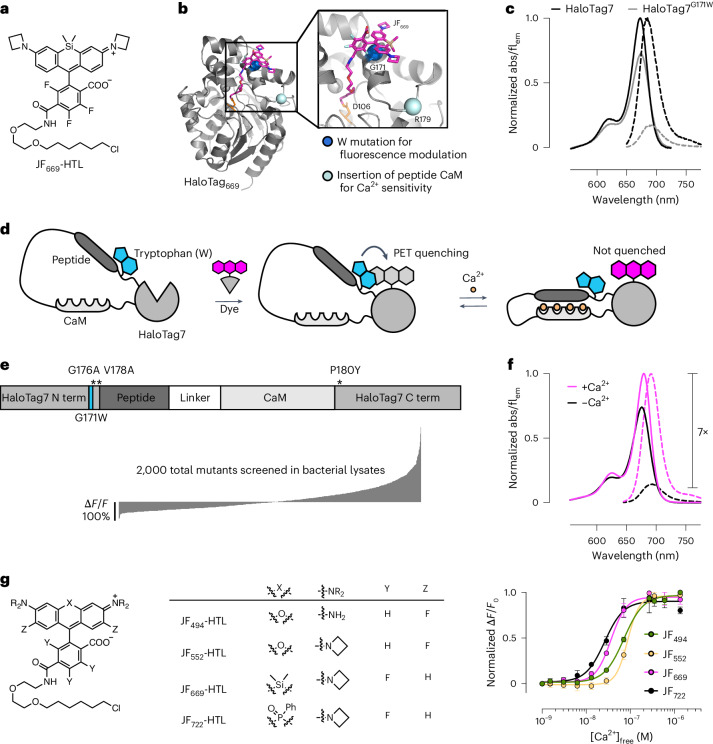
Engineering chemigenetic Ca^2+^ indicators with tryptophan quenching. **a**, Chemical structure of the JF_669_-HaloTag ligand (HTL). **b**, Crystal structure of HaloTag7 bound to the JF_669_-HaloTag ligand (HaloTag_669_) (PDB 8SW8). The positions of G171, which was mutated to a tryptophan to quench dye fluorescence emission, and R179, where Ca^2+^-sensitive protein domains were inserted, are highlighted as spheres. **c**, Normalized absorption (abs; solid lines) and fluorescence emission (fl_em_; dashed lines) spectra of the JF_669_-HaloTag ligand bound to HaloTag7 or the HaloTag7^G171W^ mutant. **d**, Schematic representation of WHaloCaMP, showing domain arrangement, covalent binding of the dye-ligand and the quenching tryptophan. **e**, Primary structure of WHaloCaMP1a (top) and ∆*F*/*F*_0_ values of variants (bottom) from a bacterial lysate screen to select WHaloCaMP1a. Term, terminus. **f**, Normalized absorption (solid lines) and fluorescence emission (dashed lines) spectra of the JF_669_-HaloTag ligand bound to purified WHaloCaMP1a in the presence (magenta) and absence (black) of Ca^2+^. **g**, Chemical structures of the dye-ligands used here with WHaloCaMP1a (left) and normalized Ca^2+^ titrations of WHaloCaMP1a bound to these dye-ligands (right). Data points represent mean and s.d. from *n* = 3 replicates. [Ca^2+^], Ca^2+^ concentration.

To construct a bright, near-infrared chemigenetic Ca^2+^ sensor for use in animals, we focused on the JF_669_-HaloTag ligand (Fig. 1a). This dye-ligand has emission just on the edge of the near-infrared and has previously been reported to efficiently label neurons in mouse brains following intravascular injection^11,12^. We did not concentrate on environmentally sensitive dye-ligands such as JF_635_-HaloTag ligand, which we previously used to engineer the HaloCaMP chemigenetic Ca^2+^ indicators, as we could not detect the JF_635_-HaloTag ligand in the central nervous system of mice following vascular injection. Fluorescence changes in the HaloCaMP sensors relied on modulating the environment of the dyes via protein conformational changes driven by Ca^2+^ binding. The environmental sensitivity of rhodamine dyes is characterized by the equilibrium between the nonfluorescent lactone and the fluorescent zwitterionic forms. In previous work^10,11,13^, we found that the lactone–zwitterion equilibrium constant (*K*_L–Z_) of a rhodamine dye is a descriptor that largely predicts both bioavailability in the central nervous system and the environmental sensitivity of dye-ligand derivatives (Supplementary Note 1). Unfortunately, the optimal range of *K*_L–Z_ for bioavailability shows little overlap with the optimal range for environmental sensitivity. Thus, the excellent bioavailability of the JF_669_-HaloTag ligand is at the expense of environmental sensitivity; this dye-ligand does not exhibit large Ca^2+^-dependent fluorescence changes when used with the previously published HaloCaMP sensors (Supplementary Fig. 1).

Instead of tuning the lactone–zwitterion equilibrium to find a dye with suboptimal bioavailability and environmental sensitivity, we hypothesized that we could generate Ca^2+^-binding-induced fluorescence changes via reversible quenching of the dye by tryptophan from the protein through PET. Tryptophan mutants of HaloTag have previously been used to quench the fluorescence of rhodamine dyes^14^ and to improve the dynamic range of fluorescent indicators^15^. To guide our engineering efforts, we solved the crystal structure of the JF_669_-HaloTag ligand bound to HaloTag7 (Fig. 1b, Protein Data Bank (PDB) 8SW8 and Supplementary Table 1) and found that G171 was within the required spatial distance of ~5 Å for fluorescence quenching through PET^16^. Mutating G171 on HaloTag7 to a tryptophan decreased fluorescence emission of bound JF_669_ by 85% (Fig. 1c), providing a way to strongly modulate the dye fluorescence emission.

**Extended Data Fig. 1:**
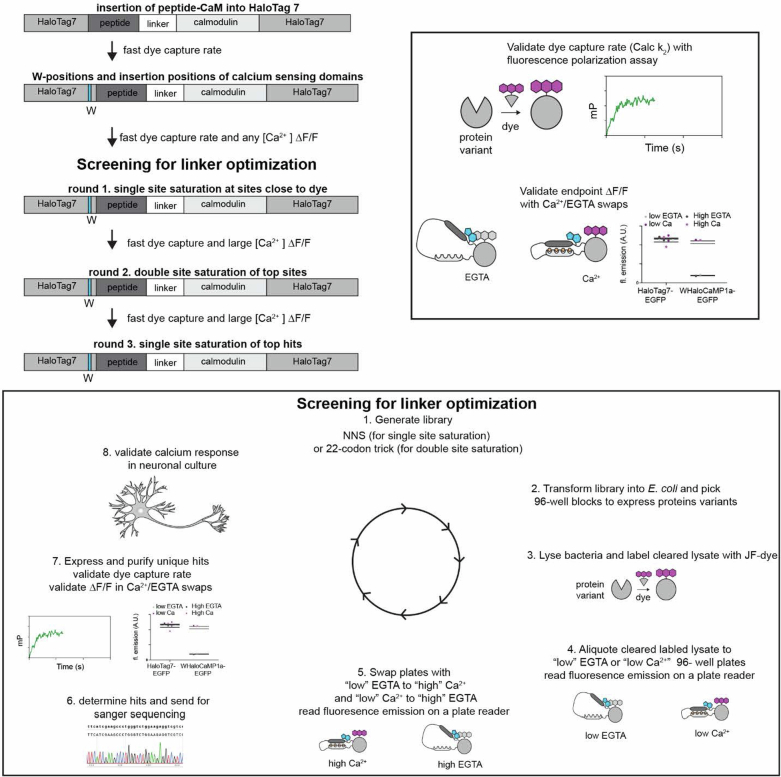
Engineering strategy for WHaloCaMPs. Engineering of WHaloCaMPs was performed in several consecutive steps. First, insertion sites of Calmodulin (CaM) and a CaM-binding peptide were explored. Insertion sites with high dye capture rate were moved forward to explore rational tryptophan mutations together with insertions. The variants with fastest dye capture rate and any fluorescence modulation upon calcium addition were moved forward to targeted directed evolution through single or double site saturation mutagenesis. After each round, Ca^2+^ indicator performance was validated in cultured neurons in a field stimulation assay.

**Extended Data Fig. 2:**
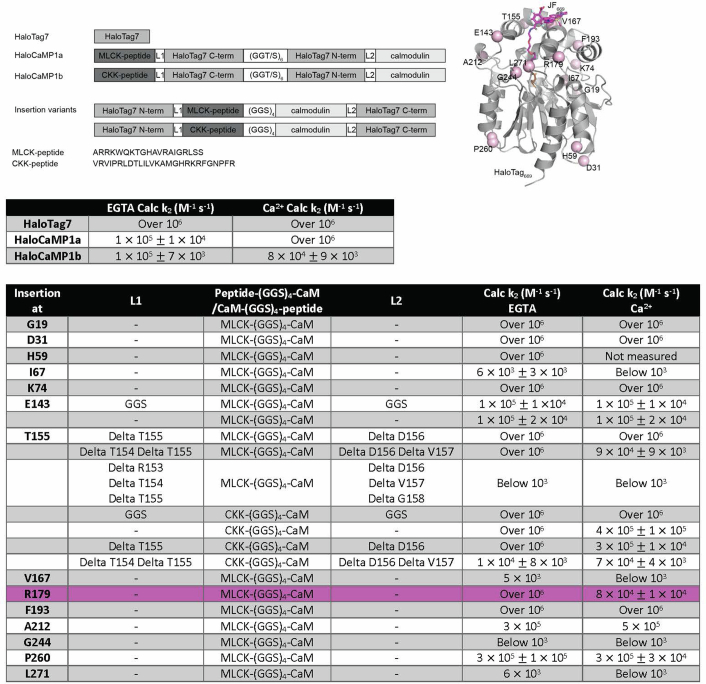
Insertion of Ca^2+^sensing domains into HaloTag7. 1D schematic of the topology of the insertion variants of CaM and CaM-binding peptide into HaloTag7 in comparison to HaloCaMP. The crystal structure of HaloTag7 bound to JF_669_-HaloTag ligand is shown, highlighting the insertion positions. Bottom two tables report the measured dye capture rate with JF_549_-HaloTag ligand and the indicated protein. The insertion position (R179) which led to WHaloCaMP1a is highlighted in magenta.

**Extended Data Fig. 3:**
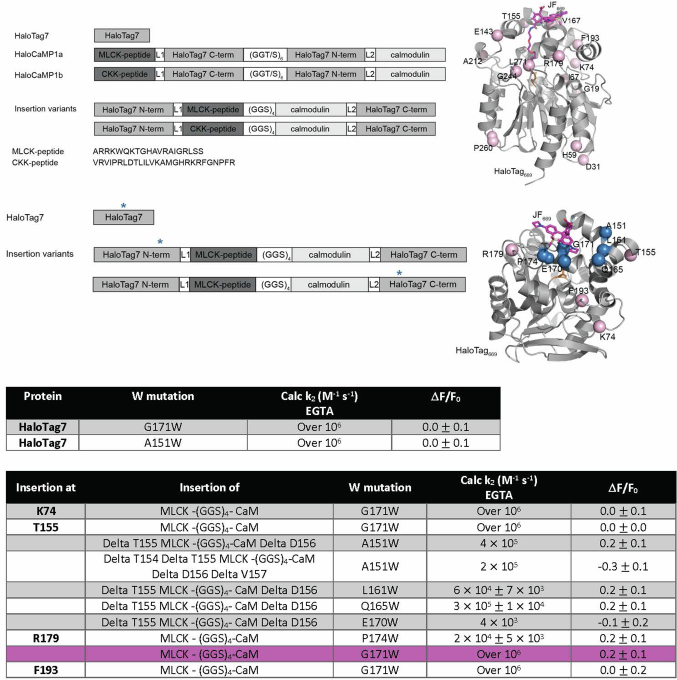
Tryptophan placements for WHaloCaMP development. 1D schematic of the topology of insertion variants of CaM and CaM-binding peptide into HaloTag7 (top left). Tryptophan positions are annotated with a blue star. The crystal structure of HaloTag7 bound to JF_669_-HaloTag ligand is shown, highlighting the insertion positions and tryptophan placements (top right). Bottom tables show the measured dye capture rate with JF_549_-HaloTag ligand and the indicated protein, and any change in fluorescence emission ΔF/F_0_ with JF_669_-HaloTag ligand. The insertion position (R179), and the tryptophan placement (G171W) which led to WHaloCaMP1a is highlighted in magenta.

**Extended Data Fig. 4:**
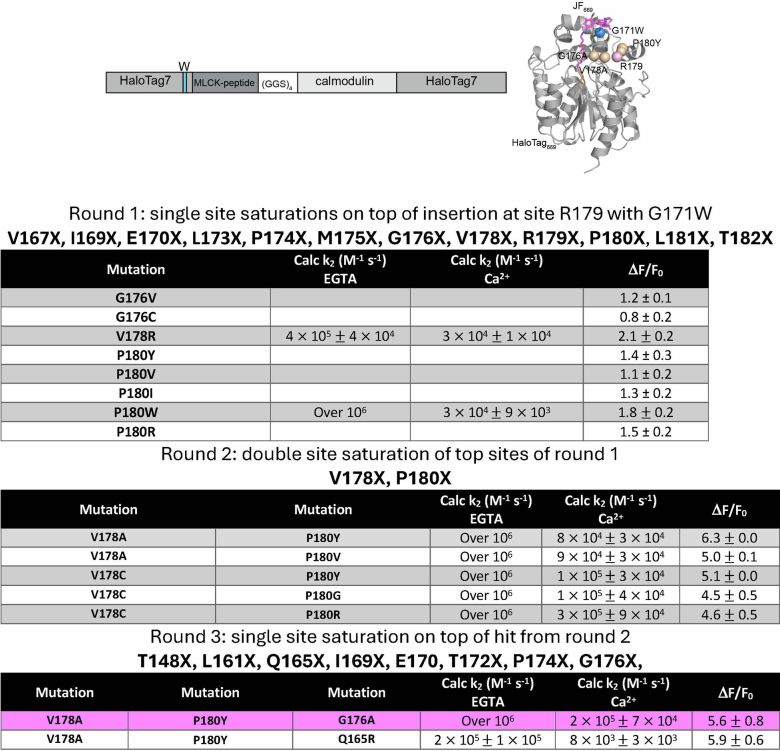
Design of WHaloCaMP1a. 1D schematic of the topology (top left) of the insertion variants of CaM and CaM-binding peptide into HaloTag7 with tryptophan position and highlighted residues on the crystal structure of HaloTag_669_ (top right). Tables report top hits from sequential mutagenic library screens. First, sites close to the dye binding site were chosen for single site saturation. Double site saturation was then performed on the top two sites from the first round of screening. A third round of single site saturation on top of the be best performing variant was then performed with sites close to the dye binding site. After each round, dye capture rates of top hits were measured by fluorescence polarization using JF_549_-HaloTag ligand and a calcium endpoint assay measured to validate fluorescence modulation by calcium using JF_669_-HaloTag ligand. WHaloCaMP1a is highlighted in magenta.

We next sought to couple the fluorescence quenching of HaloTag-bound JF_669_ to Ca^2+^-induced conformational changes in the protein (Fig. 1d and Extended Data Fig. 1). We explored different topologies of peptide calmodulin (CaM) insertions into HaloTag^G171W^ at positions spatially proximal to the bound dye and screened for insertions that retained a high dye-ligand-capture rate close to that of HaloTag7 (above 10^6^ M^−1^ s^−1^)^17^ to ensure good labeling with dye-ligand following bolus loading into complex tissue. Insertion of the myosin light-chain kinase (MLCK) CaM-binding peptide followed by CaM at position R179 of HaloTag7^G171W^ resulted in a protein with a fast dye-ligand-capture rate and a modest Ca^2+^-dependent change in fluorescence (Extended Data Figs. 2 and 3). We then performed three rounds of directed evolution on this scaffold with single- and double-site saturation mutagenesis at positions close to the insertion site and the dye–protein interface (Fig. 1e and Extended Data Fig. 4). In total, we screened ~2,000 variants in bacterial lysates and validated ten variants in primary rat hippocampal neuronal culture. We named the variant with the best performance WHaloCaMP1a: it has the fastest dye-ligand-capture rate, the largest change in fluorescence intensity on addition of Ca^2+^, reasonable Ca^2+^ kinetics (Supplementary Fig. 2), a low one-photon bleaching rate in the absence of Ca^2+^ (Supplementary Fig. 3) and the best performance in neuronal culture. We chose the name WHaloCaMP because the one-letter abbreviation for tryptophan is W, HaloTag is used to localize the dye-ligand to the protein sensor (Halo) and it is a Ca^2+^-modulated protein (CaMP).

WHaloCaMP1a_669_ showed a small increase in absorbance (1.4×) upon binding Ca^2+^ but a larger increase in fluorescence quantum yield (6.2×), which produced an overall Ca^2+^-induced fluorescence emission increase of 7× at 690 nm (Fig. 1f and Table 1). WHaloCaMP1a_669_ is more than twice as bright as jGCaMP8s^18^ and 40× brighter than the brightest biliverdin-based near-infrared Ca^2+^ indicator reported to date, iGECI^7^ (Supplementary Table 2), in the Ca^2+^-bound state and shows favorable maximum fluorescence change, Ca^2+^ affinity and kinetics of fluorescence change. WHaloCaMP1a exhibits increased fluorescence emission upon addition of Ca^2+^, making it a ‘positive-going’ indicator, while iGECI and most biliverdin-binding Ca^2+^ indicators show decreased fluorescence upon addition of Ca^2+^.

**Table 1:** Photophysical properties of WHaloCaMP1a bound to dye-ligands

WHaloCaMP1a	Abs_apo_ (nm)	Abs_sat_ (nm)	Ex/Em_apo_ (nm)	Ex/Em_sat_ (nm)	Dynamic range (*F*_max_/*F*_min_)	*K*_d_ (nM)	Hill coeff.	*ε*_apo_ (×1,000) (M^−1^ cm^−1^)	*ε*_sat_ (×1,000) (M^−1^ cm^−1^)	*Φ*_apo_	*Φ*_sat_	Brightness_apo_ (mM^−1^ cm^−1^)	Brightness_sat_ (mM^−1^ cm^−1^)
JF_494_-HTL	505	502	504/524	502/522	10	71 ± 3	1.9 ± 0.1	138	144	<0.05	0.20	N.D.	28.8
JF_552_-HTL	561	562	559/578	565/582	4	87 ± 5	3.1 ± 0.7	73	78	0.05	0.25	3.6	19.5
JF_669_-HTL	675	678	677/688	679/690	7	37 ± 2	2.5 ± 0.3	127	174	0.08	0.50	10.1	87.0
JF_722_-HTL	726	730	728/743	732/748	16	26 ± 2	2.0 ± 0.3	99	119	<0.05	0.16	N.D.	19.0

The quantum yield was too low to be measured. Abs, absorbance; apo, in presence of EGTA; max, maximum; min, minimum; sat, in presence of saturating Ca^2+^; Ex, excitation maximum; Em, emission maximum; *K*_d_, estimated dissociation constant for Ca^2+^ or half-maximum effective concentration (EC_50_) from titration curves; Hill coeff., Hill coefficient; *ε*, molar extinction coefficient; *Φ*, quantum yield; brightness (mM^−1^ cm^−1^), the product of molar extinction coefficient × quantum yield divided by 1,000; N.D., not determined.

**Extended Data Fig. 5:**
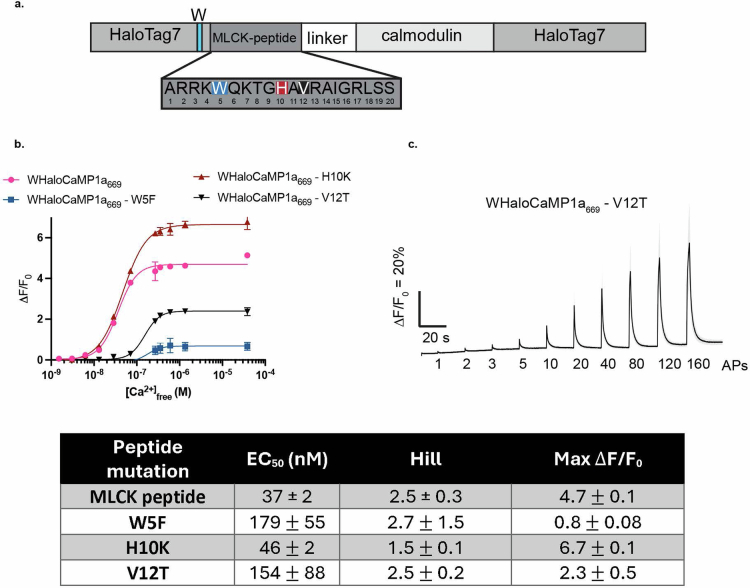
Reduced Ca^2+^ affinity of WHaloCaMP by mutations. **a**, Previously described mutations in the CaM-binding peptide were mapped onto WHaloCaMP1a. **b**, Ca^2+^ titrations of MLCK peptide variants (top) and values extracted from curve fits (bottom table). Titrations are plotted as the mean and s.d. of two technical replicates. **c**, Fluorescence trace (black) from WHaloCaMP1a_669_-V12T in primary neuron culture with field stimulation. Grey trace is s.e.m. F_0_ was calculated 1 s before the first field stimulus. WHaloCaMP1a_669_-V12T showed faster fluorescence decay in neurons compared to WHaloCaMP1a, but lower ∆F/F_0_ response at single AP, therefore WHaloCaMP1a_669_ was used for *in vivo* demonstrations.

**Extended Data Fig. 6:**
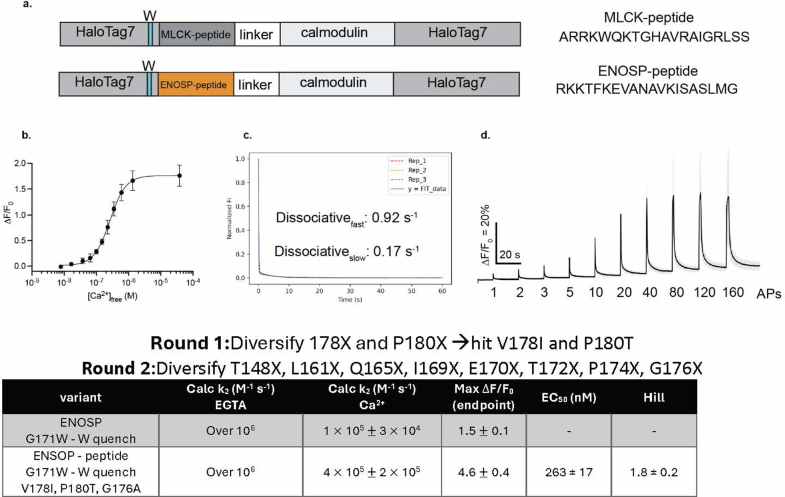
Peptide swap in WHaloCaMP1a for faster Ca^2+^ kinetics. **a**, The MLCK peptide used in WHaloCaMP1a (top) was replaced with the ENOSP-peptide used in jGCaMP8 series (bottom). Two rounds of directed evolution were performed on the new scaffold: the first round was a double site saturation of sites important in the evolution of WHaloCaMP1a; the second was single site saturation on top of the top hit from the first round, which gave a variant (V178I, P180T, G176A) with fast dye capture rate, lower Ca^2+^ affinity (**b**.) titrations is plotted as the mean and s.d. of three technical replicates, and fast Ca^2+^ kinetics measured by stopped flow (**c**.). **d**, A representative fluorescence trace from a field stimulation (black) from one run of the field stimulation assay with ~40 neuron ROIs. Grey trace is standard error of the mean (s.e.m.) of the fluorescence trace. F_0_ was calculated 1 s before the first field stimulus. Table reports fits from experiments in panels **b**. Even though the ENOSP variant had faster Ca^2+^ kinetics in neurons, it had lower ∆F/F_0_ response at single AP, therefore WHaloCaMP1a_669_ was used for *in vivo* demonstrations.

**Extended Data Fig. 7:**
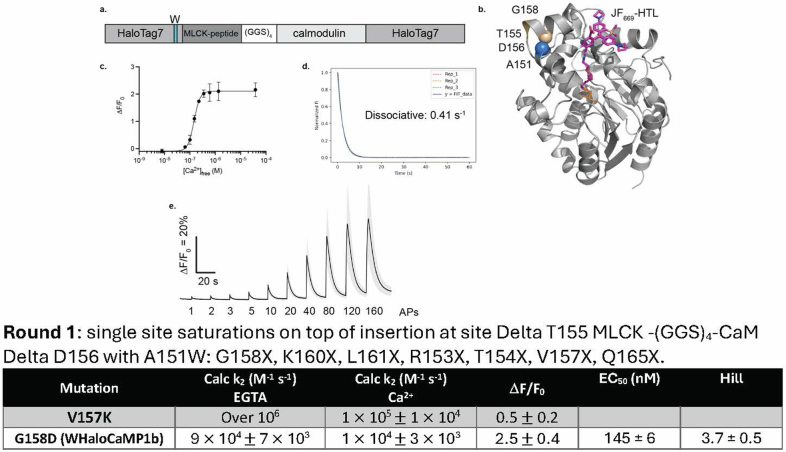
Design and characterization of WHaloCaMP1b. **a**, The MLCK CaM-binding peptide and CaM were inserted at HaloTag T154, deleting T155 and D156. One round of single site saturation mutagenesis produced hits with improved calcium response (G158D). Further rounds of directed evolution did not give better variants. **b**, Crystal structure of HaloTag7_669_ highlighting relevant positions. **c**, ∆F/F_0_ vs. [Ca^2+^] of WHaloCaMP1b_669_. Titrations is plotted as the mean and s.d. of three technical replicates. **d**, Stopped-flow Ca^2+^ kinetics of unbinding for WHaloCaMP1b_669_. **e**, Representative trace (black) from one run of the field stimulation assay with ~40 neuron ROIs. Grey trace is standard error of the mean (s.e.m.) of the fluorescence trace. F_0_ was calculated 1 s before the first field stimulus. The table reports fits from experiments in panels **c**.

WHaloCaMP proved to be amenable to further modification of its properties based on previous GECI engineering efforts. We decreased the Ca^2+^ affinity by single point mutations in the MLCK CaM-binding peptide^19^ (Extended Data Fig. 5) and produced faster response kinetics by substituting the CaM-binding peptide with endothelial nitric oxide synthase peptide (ENOSP), previously used in the jGCaMP8 series^18^ (Extended Data Fig. 6). We also explored other topologies of WHaloCaMP. Several protein engineering efforts^20,21^ have focused on a loop close to the dye-binding site at position T155 of HaloTag7. We generated WHaloCaMP1b by inserting MLCK CaM at position T154 and mutating A151 to tryptophan (Extended Data Fig. 7). We further characterized WHaloCaMP1a in this work because it showed larger fluorescence changes, but we note that the WHaloCaMP1b topology also appears to be a reasonable solution to generating chemigenetic indicators.

**Extended Data Fig. 8:**
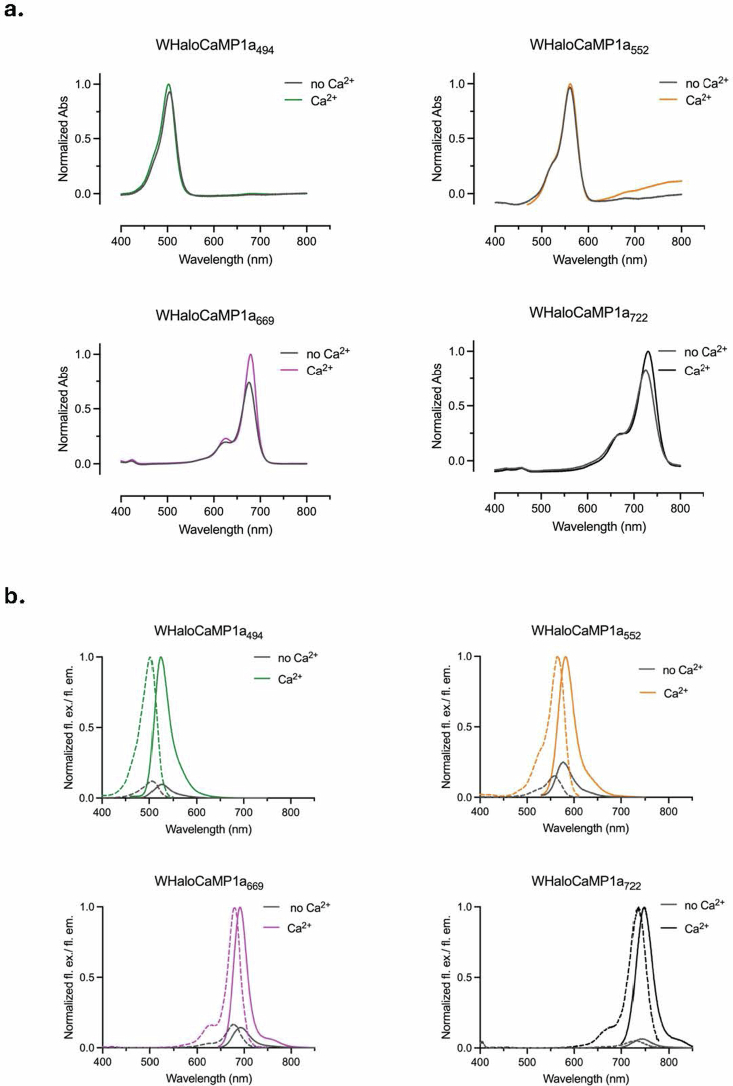
Absorption and fluorescence spectra for WHaloCaMP1a. WHaloCaMP1a bound to JF_494_-HaloTag ligand, JF_552_-HaloTag ligand, JF_669_-HaloTag ligand and JF_722_-HaloTag ligand. Absorption (**a**). and fluorescence excitation and emission spectra (**b**.). Spectra were normalized to max absorbance or flourescence exitation or emission in the Ca^2+^ state.

A benefit of chemigenetic indicators is that the color of fluorescence emission of the indicator can be altered by changing the small-molecule dye-ligand. In addition to the near-infrared-emitting JF_669_-HaloTag ligand, we explored WHaloCaMP1a bound to other rhodamine-based dyes, including the green-emitting JF_494_-HaloTag ligand, based on 2,7-difluororhodamine 110 (ref. ^22^), the orange-emitting JF_552_-HaloTag ligand^13^ and the near-infrared-emitting JF_722_-HaloTag ligand^11^ (Fig. 1g, Extended Data Fig. 8 and Supplementary Figs. 4 and 5). Consistent with a tryptophan PET quenching mechanism, fluorescence changes were mainly conferred by changes in the quantum yield of each bound dye (Table 1). WHaloCaMP1a displayed a high Ca^2+^ affinity (26 ± 2 nM to 71 ± 3 nM for different dyes; Fig. 1g and Table 1), similar to that of jGCaMP8s (46 ± 1 nM)^18^. The WHaloCaMP1a sensor bound to different dye-ligands showed Ca^2+^-dependent fluorescence changes from pH 5 to 9. WHaloCaMP1a_669_ was particularly stable, exhibiting little change in Ca^2+^ response in the pH range of 6–8 (Supplementary Fig. 6). The relative pH insensitivity indicates that WHaloCaMP1a could be used in a wide range of cellular compartments.

### WHaloCaMP performance in neuron cultures and brain slices

**Fig. 2:**
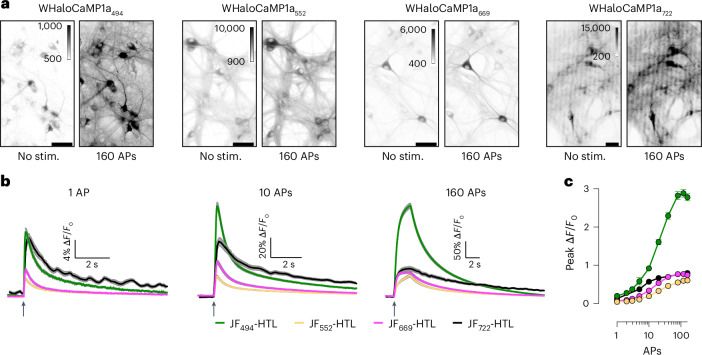
Characterization of WHaloCaMP1a in neuronal cultures. **a**, Representative images of cultured rat hippocampal neurons expressing WHaloCaMP1a labeled with dye-ligands unstimulated or stimulated with 160 induced APs. Scale bars, 50 µm. Stim., stimulation. **b**, The ∆*F*/*F*_0_ response of WHaloCaMP1a expressed in cultured rat hippocampal neurons and labeled with the indicated dye-ligands to trains of APs. Solid line (mean) and gray outline (s.e.m.) for *n* = 153, 168 and 141 neurons for the JF_494_-HaloTag ligand, the JF_552_-HaloTag ligand and the JF_669_-HaloTag ligand and for *n* = 20 for the JF_722_-HaloTag ligand. Black arrows indicate the start of stimulation. **c**, Peak ∆*F*/*F*_0_ as a function of the number of APs. Data are presented as mean and s.e.m. for *n* = 153, 168 and 141 neurons for the JF_494_-HaloTag ligand, the JF_552_-HaloTag ligand and the JF_669_-HaloTag ligand and for *n* = 20 for the JF_722_-HaloTag ligand. APs were elicited with a field stimulation electrode with a pulse width of 1 ms at 80 Hz and 40 V.

We expressed WHaloCaMP1a in primary rat hippocampal neurons in culture, labeled with dye-ligands and elicited action potentials (APs) evoked by electrical stimulation with a field electrode (Fig. 2a, Supplementary Fig. 7 and Supplementary Tables 3 and 4). WHaloCaMP1a could detect single APs with a ∆*F*/*F*_0_ value of up to 20% for WHaloCaMP_494_ (Fig. 2b). WHaloCaMP_722_ is the furthest red-shifted chemigenetic fluorescent indicator that can follow single-AP transients in neurons reported to date. In our hands, WHaloCaMP1a_669_ showed a larger max ∆*F*/*F*_0_ value in neurons than the brightest biliverdin-binding Ca^2+^ indicator, iGECI (Fig. 2c and Supplementary Tables 3 and 4). In addition, WHaloCaMP1a_669_ showed faster time to peak and half decay time than iGECI (Supplementary Figs. 7 and 8), making it better suited to capture neuronal activity.

The excitation spectrum of WHaloCaMP1a_669_ is better separated from the action spectrum of many blue-excited optogenetic tools than that of red Ca^2+^ indicators. We found via electrophysiology that the light required for exciting WHaloCaMP1a_669_ did not produce any observable membrane depolarization in neurons expressing the sensitive channelrhodopsin variant CheRiff^23^ (Supplementary Fig. 9). Indeed, even at a light intensity exceeding ten times the power required for functional imaging with WHaloCaMP1a_669_, no depolarization was observed. By contrast, the light required for imaging the commonly used red fluorescent protein-based Ca^2+^ indicator jRCaMP1a produced substantial subthreshold depolarization of neurons expressing CheRiff.

The near-infrared emission of WHaloCaMP1a_669_ also allows for multiplexing with current fluorescent protein-based indicators. We performed simultaneous multiplexed two-photon imaging of fluctuating Ca^2+^ and protein kinase A (PKA) activity in response to muscarinic acetylcholine receptor (mAChR) and adenylate cyclase activation in acute mouse brain slices using WHaloCaMP1a_669_ and a green PKA activity indicator, FLIM-AKAR^24^ (Supplementary Fig. 10). Consistent with previous results with single-reporter imaging^25,26^, we observed a concurrent Ca^2+^ and PKA activity increase in response to mAChR activation and only increased PKA phosphorylation in response to adenylate cyclase activation. Because a Ca^2+^ increase is upstream of PKA activation, these results demonstrate the possibility of tracking concurrent dynamics of multiple signals to understand temporal transformations in a signaling transduction cascade.

### WHaloCaMP1a reports neuronal Ca^2+^ in flies and mice

**Fig. 3:**
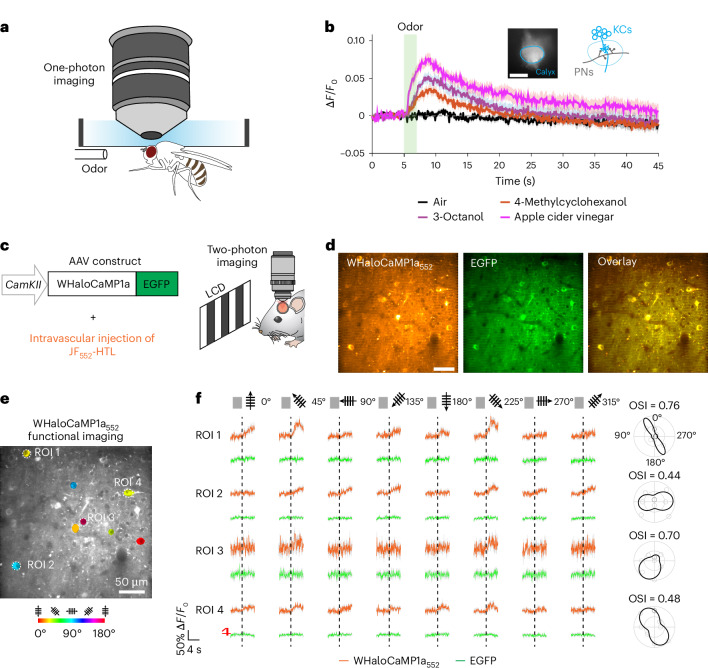
WHaloCaMP1a reports on neuronal activity in flies and mice. **a**, One-photon imaging setup of head-fixed flies expressing WHaloCaMP1a labeled with dye-ligands. **b**, Fluorescence responses from WHaloCaMP1a_669_ in head-fixed flies presented with different odors. WHaloCaMP1a was expressed in mushroom body KCs. Images were acquired from the calyx, where KCs receive dendritic inputs from the olfactory projection neurons (PNs) (insets). Green shading indicates odor presentation for 2 s. Data were from six flies, and odors were presented three times to each fly. The thick line and the shaded areas indicate means and s.e.m. across odor trials. Scale bar, 50 µm. **c**, AAV construct for transducing neurons in the mouse V1 and the schematic of the experimental setup for two-photon functional imaging of WHaloCaMP1a in the visual cortex of mice. The JF_552_-HaloTag ligand was intravascularly injected 1 d before examining orientation selectivity of V1 neurons in the anesthetized mouse exposed to moving grafting visual stimuli of different orientations and directions. LCD, liquid crystal display. **d**, Representative images of a field of view in the mouse V1 showing neurons expressing WHaloCaMP1a_552_ or EGFP. Scale bar, 50 µm. **e**, Functional imaging of V1 neurons shows the orientation selectivity map. **f**, Functional imaging of Ca^2+^ (WHaloCaMP1a_552_ channel) or control (EGFP channel) traces (ROI 1–4) in response to drifting gratings in the directions shown above the traces. Average of five trials. Colored lines indicate means, and shadows indicate s.d. Orientation selectivity index (OSI) of cells is shown on the right. Imaging rate was 15 Hz. A representative imaging session from three imaging sessions is shown. The experiment was repeated independently 19 times in four mice with similar results.

Because previous chemigenetic HaloTag-based sensors, such as HaloCaMPs, were limited to experiments in reduced preparations, we explored whether WHaloCaMP1a could be expressed, labeled with dye-ligands and used to follow physiological Ca^2+^ responses in living animals. Although WHaloCaMP1a_722_ showed large responses in primary cultured neurons (Fig. 2), the JF_722_-HaloTag ligand did not appear to be bioavailable in vivo (Supplementary Note 1). Instead, we focused on the dyes that showed the best in vivo bioavailability: the JF_552_-HaloTag ligand and the JF_669_-HaloTag ligand. First, we tested whether WHaloCaMP1a could report odor-evoked Ca^2+^ responses in mushroom body Kenyon cells (KCs) in adult *Drosophila* (Fig. 3a). KCs are third-order neurons in the fly olfactory pathway, and their odor response profile has been extensively characterized^27^. We expressed WHaloCaMP1a in KCs with the mushroom body driver R13F02-Gal4. We then opened a small window in the head capsule of flies to expose the calyx, which houses the dendritic processes of all KCs (Fig. 3b), and labeled WHaloCaMP1a with JF_669_-HaloTag ligand by application in saline solution. Upon odor presentation, we observed clear WHaloCaMP1a_669_ fluorescence increases in individual trials in response to apple cider vinegar as well as two chemical odors (Fig. 3b and Supplementary Fig. 11). Similar responses were observed when we labeled WHaloCaMP with the JF_552_-HaloTag ligand, highlighting the spectral flexibility of the indicator. Trends in odor response were similar to that of the red fluorescent protein-based Ca^2+^ indicator jRGECO1a^28^ (Supplementary Fig. 12).

We then tested the performance of WHaloCaMP1a in mice. We expressed WHaloCaMP1a with an enhanced green fluorescent protein (EGFP) expression marker from the Ca^2+^–CaM-dependent protein kinase II (*CaMKII*) promoter in the mouse primary visual cortex (V1) using adeno-associated virus (AAV)-mediated gene delivery (Fig. 3c). Intravenous delivery of the JF_552_-HaloTag ligand or the JF_669_-HaloTag ligand via retro-orbital (r.o.) injection successfully labeled WHaloCaMP1a expressed in V1 excitatory neurons (Fig. 3d and Supplementary Figs. 13 and 14). We observed clear visually evoked Ca^2+^ transients in layer 2/3 excitatory neurons with WHaloCaMP1a_669_ using 1,225-nm two-photon excitation in single trials (Supplementary Fig. 13). As most standard two-photon microscopes cannot achieve substantial 1,225-nm excitation, we instead labeled WHaloCaMP1a with the JF_552_-HaloTag ligand and imaged at either 850 nm or 1,050 nm. Using 850-nm light, we could co-excite the EGFP expression marker and cleanly separate the fluorescence emission, providing a nonresponsive normalization channel during two-photon functional imaging, while 1,050-nm light selectively excited WHaloCaMP1a_552_ (Supplementary Fig. 14). We performed functional imaging during presentation of drifting grating visual stimuli to the mouse’s contralateral eye and observed responses in individual trials up to 35% ∆*F*/*F*_0_ as well as orientation tuning of the responses, which is a hallmark of V1 excitatory neurons (Fig. 3e,f and Supplementary Figs. 15 and 16).

When we expressed WHaloCaMP1a_669_–EGFP or iGECI–EGFP in the S1 somatosensory barrel cortex, we observed higher expression and maximum far-red fluorescence signal from WHaloCaMP1a_669_ when imaged by one-photon microscopy (Supplementary Fig. 17). Additionally, we could observe clear functional responses of WHaloCaMP1a_669_ elicited by whisker stimulation of the mouse.

### Functional imaging in zebrafish larvae with WHaloCaMP1a_669_

**Extended Data Fig. 9:**
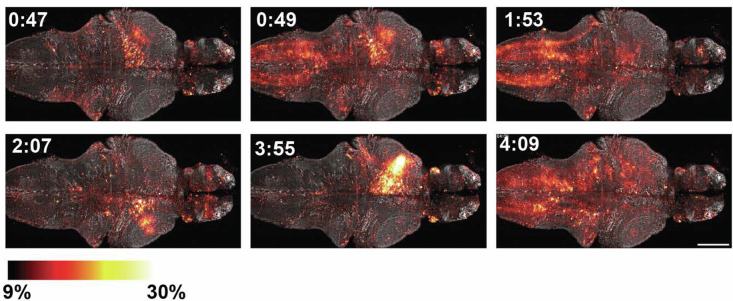
Volumetric whole brain imaging of neurons in zebrafish larvae using SiMView. Tg(*elavl3:*NES-WHaloCaMP1a-EGFP) zebrafish were labeled with JF_669_-HaloTag ligand and mounted in a SiMView light sheet microscope. Three-dimensional volumes of the brain with a 160 µm length along the z-axis were acquired with a z-step size of 4 µm and a rate of 4 volumes per second. Visualization of ∆F/F_0_ (shown using a red-hot color look-up table) displayed over a projection of the concatenated projected ∆F/F_0_ time series that serves as an anatomical reference (shown in grey). Time stamps are in min:s and scale bar is 100 µm. A movie of this whole brain time-lapse image data set can be found in Supplementary Movie 1.

We generated transgenic zebrafish expressing WHaloCaMP1a under the pan-neuronal *elavl3* promoter. First, we verified that we could perform whole-brain functional neuronal imaging with larvae at 4–5 d post fertilization (dpf) with WHaloCaMP1a_669_ using light-sheet imaging with excitation at 685 nm and 4 Hz (Extended Data Fig. 9 and Supplementary Video 1). To benchmark the performance of WHaloCaMP1a_669_ against known red Ca^2+^ indicators^28^, we crossed the *elavl3*-WHaloCaMP1a zebrafish line with a line expressing pan-neuronal red fluorescent protein-based calcium indicator jRGECO1b under the same *elavl3* promoter. We performed dual-color light-sheet imaging (excitation at 561 nm and 640 nm) from a single plane in the hindbrain of zebrafish larvae at 4–5 dpf. WHaloCaMP1a_669_ reported all the Ca^2+^ transients observed with jRGECO1b in a region of interest (ROI) in the hindbrain, albeit with lower maximum ∆*F*/*F*_0_ than the jRGECO1b signal (66% of the maximum ∆*F*/*F*_0_ signal of jRGECO1b; Supplementary Fig. 18).

### Multiplexed imaging in zebrafish larvae with WHaloCaMP1a_669_

**Fig. 4:**
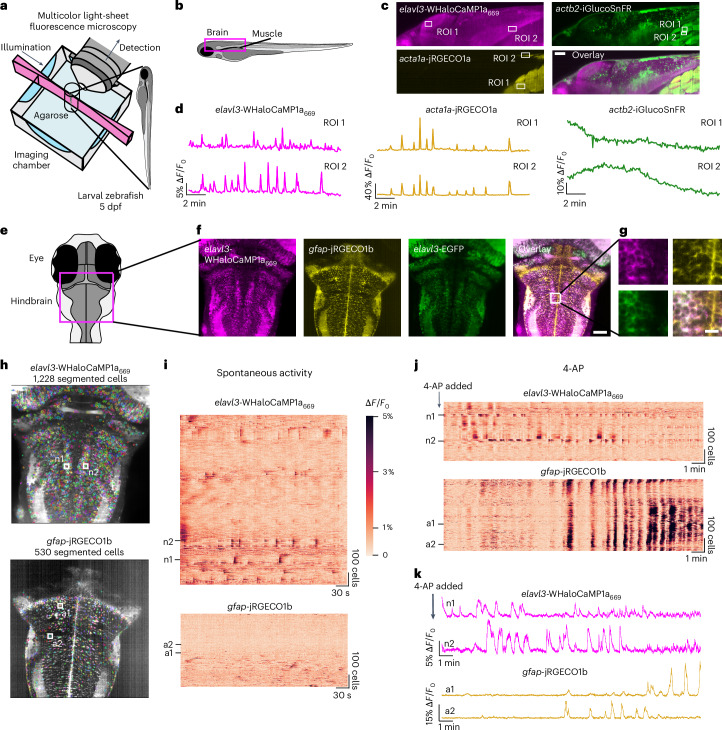
Three-color multiplexed functional imaging in zebrafish larvae. **a**, Light-sheet imaging setup for multiplexed imaging. **b**, Schematic of side-view zebrafish larvae highlighting the field of view for three-color multiplexed functional imaging of glucose and Ca^2+^ in muscles and neurons. **c**, Representative images of WHaloCaMP1a expressed in neurons from the *elavl3* promoter, iGlucoSnFR expressed from the *actb2* ubiquitous promoter and jRGECO1a expressed in muscle from the *acta1a* promoter. Scale bar, 50 µm. The experiment was repeated independently three times with similar results. **d**, Fluorescence ∆*F*/*F*_0_ traces of WHaloCaMP1a_669_, jRGECO1a and iGlucoSnFR in the ROI outlined in **b**. A representative experiment from three zebrafish larvae was imaged. **e**, Schematic of the zebrafish larva’s head indicating the field of view for light-sheet imaging of neuronal and astrocyte activity. **f**, Representative images of the expression patterns of WHaloCaMP1a_669_–EGFP expressed under the *elavl3* promoter and jRGECO1b expressed under the *gfap* promoter. Scale bar, 50 µm. The experiment was repeated independently more than three times with similar results. **g**, Zoomed-in images showing single-cell resolution of fluorescent signals in the hindbrain. Scale bar, 20 µm. **h**, Images of Suite2p- and Cellpose-segmented cells from simultaneous functional imaging of WHaloCaMP1a_669_ and jRGECO1b. **i**, Rastermaps of activity from 1,228 segmented neurons (top) and 530 astrocytes (bottom) during spontaneous brain activity. Two neurons (n1 and n2) indicate the hindbrain oscillator. Two astrocytes (a1 and a1) are also indicated. **j**, The compound 4-AP was added to the imaging chamber of the zebrafish larva imaged in **i**, and functional imaging was performed. Concatenation of three imaging blocks of 6.2 min each. **k**, Fluorescence ∆*F*/*F*_0_ traces of n1 and n2 (top) and a1 and a2 (bottom) after addition of 4-AP.

Clean spectral separation of WHaloCaMP1a_669_ from green and red fluorescent proteins enabled us to perform three-color multiplexed imaging in zebrafish larvae (Fig. 4a). To simultaneously follow three physiological signals, we crossed the transgenic line expressing pan-neuronal WHaloCaMP1a with a previously established line^29^ expressing the green-emitting glucose sensor iGlucoSnFR throughout the body of the fish using the β-actin promoter and the red-emitting Ca^2+^ indicator jRGECO1a in skeletal muscle using the α-actinin promoter. After labeling WHaloCaMP1a with the JF_669_-HaloTag ligand, we performed light-sheet imaging of a single plane in the fish (Fig. 4b) with clear spectral separation of the fluorescence emission of the three sensors (Fig. 4c, Supplementary Fig. 19 and Supplementary Video 2). Ca^2+^ transients in the zebrafish hindbrain neurons recorded using WHaloCaMP1a_669_ correlated with Ca^2+^ transients in skeletal muscle recorded using jRGECO1a, but neuronal signals in other parts of the brain, such as the optical tectum, did not (Fig. 4d). Glucose changes in muscle observed using iGlucoSnFR were substantially slower than either the neuronal or muscle Ca^2+^ transients, as previously reported^29^.

Next, we asked whether WHaloCaMP could be used to follow changes in neuronal activity to report changes in physiological states during multiplexed imaging. The activities of neurons and radial astrocytes are both linked to changes in behavior^30^, as reported by dual-color functional imaging. We imaged zebrafish larvae expressing WHaloCaMP1a_669_ in neurons and the red Ca^2+^ indicator jRGECO1b in radial astrocytes, thus freeing up the green fluorescence emission channel, here using it as a fluorescent marker for neurons (Fig. 4e,f). Using light-sheet imaging, we could clearly resolve single cells in a plane of the zebrafish hindbrain (Fig. 4g). We then performed functional imaging of paralyzed zebrafish larvae at 4 Hz and used Suite2p^31^ together with Cellpose^32^ and Rastermap^33^ (Fig. 4h and Supplementary Fig. 20) to segment and follow the activity of over 1,000 neurons and hundreds of astrocytes simultaneously during spontaneous activity (Fig. 4i and Supplementary Video 3). Without any external perturbation, WHaloCaMP1a_669_ clearly showed Ca^2+^ transients in a population of neurons that form part of the hindbrain oscillator^34^ related to motor control during swimming (n1 and n2 in Fig. 4i). Leading up to and during seizure-like states, induced by the potassium channel blocker 4-aminopyridine (4-AP), the pattern of neuronal activity in the hindbrain oscillator was lost (n1 and n2 in Fig. 4j,k and Supplementary Video 4), which has previously been described to be due to hyperexcitability^35^ of the neurons. We also observed large Ca^2+^ waves in the astrocyte population in response to 4-AP (a1 and a2 in Fig. 4j,k), as previously described^36^, which we could clearly separate from the neuronal activity, as the emissions of jRGECO1b and WHaloCaMP1a_669_ are 100 nm apart. The loss of hindbrain oscillator activity and the large astrocytic Ca^2+^ waves were not observed in fish not treated with 4-AP (Supplementary Fig. 21). WHaloCaMP1a_669_ is well suited for one-photon multicolor experiments in living animals as it can be excited with common far-red and near-infrared laser lines, and the emission is separated from commonly used fluorescent proteins.

### FLIM using WHaloCaMP1a

**Fig. 5:**
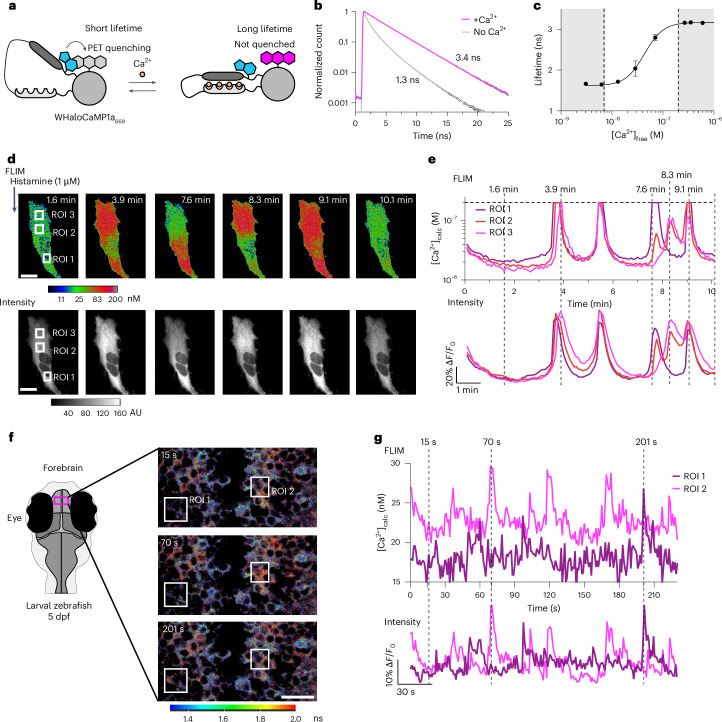
Quantitative Ca^2+^ measurements by FLIM using WHaloCaMP1a. **a**, Schematic of WHaloCaMP1a bound to a dye-ligand used as a FLIM probe. Tryptophan quenching modulates the fluorescence lifetime. **b**, Normalized fluorescence lifetime of WHaloCaMP1a_669_ in the presence or the absence of Ca^2+^, fit to a three-component fluorescence decay. **c**, Calibration curve of the averaged fluorescence lifetime of WHaloCaMP1a_669_ versus Ca^2+^ concentration. The white box indicates the range in which WHaloCaMP1a_669_ can be used to make quantitative measurements of Ca^2+^ concentration. Performed with purified protein. Mean of three replicates and s.d. are plotted. **d**, Pseudocolored concentration (top) and intensity images (bottom) of WHaloCaMP1a_669_ in HeLa cells after histamine addition. Scale bar, 20 µm. Color bar indicates Ca^2+^ concentration, calculated from a calibration curve of fluorescence lifetime. AU, arbitrary units. **e**, Quantitative Ca^2+^ concentration calculated (calc) from a FLIM calibration curve (top) and fluorescence traces ∆*F*/*F*_0_ calculated from the intensity channel (bottom) in histamine-stimulated HeLa cells in the ROI highlighted in **d**. Calibrated WHaloCaMP1a_669_ can only be used to measure Ca^2+^ concentrations up to 200 nM, indicated by a dashed horizontal line. Vertical dashed lines indicate time points in the time series at which images in **d** are shown. **f**, FLIM of WHaloCaMP1a_669_ in live zebrafish larvae showing spontaneous neuronal activity in the forebrain. The experiment was repeated independently three times with similar results. Schematic indicating the field of view during imaging (left). Overlaid images of FLIM and intensity using Leica LAS X software, with a color bar indicating the fluorescence lifetime. Scale bar, 20 µm. **g**, Ca^2+^ concentrations calculated from a FLIM calibration curve (top) and fluorescence traces ∆*F*/*F*_0_ calculated from the intensity channel (bottom) over time for two neurons in the forebrain of zebrafish larvae from the ROI indicated in **f**. Dashed lines indicate time points of images in **f**. Representative images from three imaging sessions.

The fluorescence modulation in WHaloCaMP1a on binding to Ca^2+^ is mainly due to changes in quantum yield. We thus explored WHaloCaMP1a as a FLIM probe for Ca^2+^ (Fig. 5a). Fluorescence lifetime is an inherent property of fluorophores, and FLIM allows absolute concentrations to be determined using fluorescent biosensors, which allows comparison of analytes across imaging days, samples and animals^37^. FLIM imaging has been used to determine how Ca^2+^ concentrations differ in astrocytes and neurons during development^38^ and during affinity maturation of B cells in living mice^39^. However, the technique is limited by the number of photons that can be collected for accurate fitting of fluorescent lifetimes.

**Extended Data Fig. 10:**
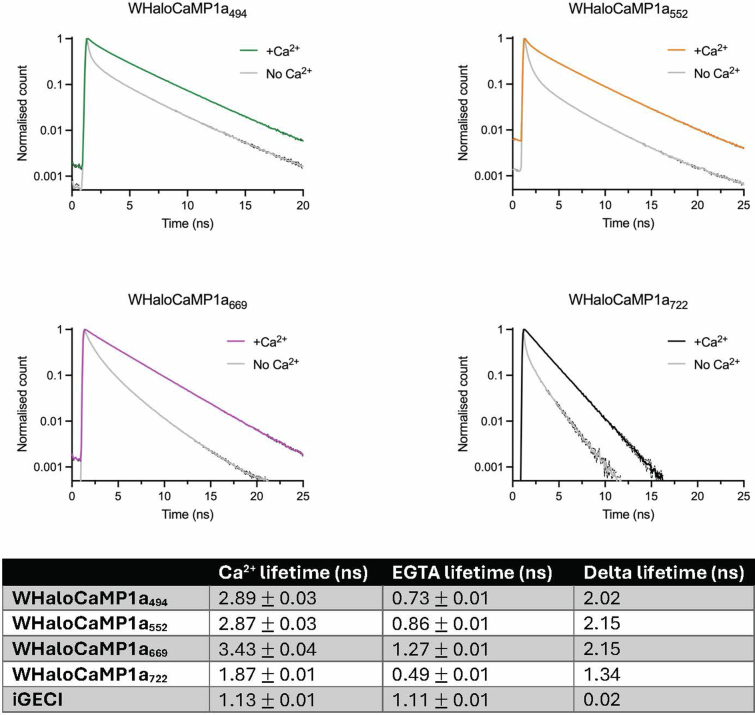
Fluorescence lifetimes of WHaloCaMP1a and iGECI. Fluorescence lifetime image microscopy (FLIM) with WHaloCaMP1a bound to JF_494_-HaloTag ligand, JF_552_-HaloTag ligand, JF_669_-HaloTag ligand or JF_722_-HaloTag ligand, and iGECI. A time domain FLIM set-up was used at 40 MHz. The lifetime decay was fit to a three-component fit for WHaloCaMP1a. A two component fit was performed for iGECI. No additional biiverdin was added to the iGECI sample before imaging. The amplitude weighted lifetime is reported. Mean and standard deviation from three replicates from at least two different samples.

WHaloCaMP1a bound to JF_494_, JF_552_, JF_669_ and JF_722_ could all be used as FLIM probes for Ca^2+^ (Extended Data Fig. 10). As WHaloCaMP1a_669_ is brightly fluorescent, we reasoned that it would perform especially well as a FLIM probe in living cells and animals. In purified protein solutions, we observed a 2.1-ns increase in the fluorescence lifetime of WHaloCaMP1a_669_ upon Ca^2+^ binding (lifetime of calcium-free *τ*_apo_ = 1.3 ns versus lifetime of calcium-saturated *τ*_sat_ = 3.4 ns) (Fig. 5b). We then generated calibration curves of lifetime versus Ca^2+^ concentration both with purified protein and with HeLa cells expressing WHaloCaMP1a_669_ that were permeabilized by digitonin (Fig. 5c and Supplementary Fig. 22). We used these calibration curves to follow Ca^2+^ dynamics in HeLa cells stimulated with histamine (Fig. 5d, Supplementary Fig. 23 and Supplementary Video 5). As the Ca^2+^ affinity of WHaloCaMP1a_669_ is higher than that of the previously reported turquoise-emitting FLIM Ca^2+^ indicator Tq-Ca-FLITS^40^, we could more accurately determine low Ca^2+^ concentrations, down to 7 nM (Fig. 5e). We measured over 100-fold changes in Ca^2+^ concentration during histamine-induced oscillations in HeLa cells. To determine whether WHaloCaMP allowed in vivo FLIM Ca^2+^ imaging, zebrafish larvae expressing WHaloCaMP1a were labeled with JF_669_-HaloTag ligand and imaged with a commercial confocal FLIM microscope (Fig. 5f and Supplementary Fig. 24). We could follow dynamic changes in absolute intracellular Ca^2+^ concentration in populations of forebrain neurons at single-cell resolution during spontaneous activity at one frame per second (Fig. 5g and Supplementary Video 6), and Ca^2+^ events seen by FLIM matched well with those observed via fluorescence intensity changes. WHaloCaMP therefore allows for the quantitative readout of physiological signaling at cellular resolution across tissues in vivo.

## Discussion

Despite their favorable photophysical properties, synthetic dyes have not been fully exploited to build chemigenetic functional indicators with applications in living animals, especially in the near-infrared. This has mostly been due to challenges with delivering the dyes in complex tissue and in vivo. Here, we started with a near-infrared dye-ligand, JF_669_-HaloTag ligand, which we knew could be delivered to many tissues in mice after vascular injection, and built a Ca^2+^ indicator around it. Because the JF_669_-HaloTag ligand did not work with our existing chemigenetic indicators, we used a different mechanism to modulate its fluorescence output: PET quenching by a strategically placed tryptophan. This strategy allowed us to record Ca^2+^ transients in the near-infrared channel in vivo in several model organisms.

The chemigenetic approach leads to several advantageous properties of WHaloCaMP compared to biliverdin-binding protein-based indicators. WHaloCaMP1a_669_ is up to 40× brighter than biliverdin-binding indicators. For some applications, the endogenous availability of biliverdin cofactor in cells is advantageous, as supplementation of the exogenous cofactor is not required^6,7^. However, the affinity for biliverdin is often weak in the Ca^2+^ indicators built from biliverdin-binding proteins, making them dim when used in animals. As opposed to the JF_669_-HaloTag ligand, biliverdin does not cross the blood–brain barrier in mice; therefore, additional biliverdin must be generated in situ by either delivery of additional biliverdin-producing enzymes^41^ or knockouts of biliverdin-consuming proteins^42^. A further advantage of WHaloCaMP’s chemigenetic nature over fixed chromophore systems is that the color of the fluorescence emission can be tuned depending on the experimental requirements. Here, we show that the same genetically encoded protein sensor, WHaloCaMP1a, can be used with a range of rhodamine dye-ligands with green to near-infrared emission for functional imaging. WHaloCaMP1a thus allows flexibility to match the excitation and emission requirements for specific imaging hardware or for spectrally multiplexed functional imaging.

WHaloCaMP was amenable to known protein engineering strategies to alter Ca^2+^ affinity and binding kinetics, and we envision further protein engineering to optimize maximum fluorescence change. Given the breadth of existing fluorescent protein^43^ and HaloTag or dye-based functional indicators^44^, we anticipate that the tryptophan-quenching chemigenetic indicator approach can be extended to in vivo imaging of other physiological signals. Additionally, as we learn more about the bioavailability of dye-ligands in animals, we envision additional chemigenetic approaches in which the specificity of genetically encoded tools is combined with the brightness, the photostability and the tunability of synthetic dyes.

## Methods

### Molecular biology

Synthetic DNA oligonucleotides were purchased from Twist Bioscience and Integrated DNA Technologies. Q5 High-Fidelity DNA Polymerase (New England Biolabs) was used for all PCR amplifications. Isothermal assembly reactions were performed with a NEBuilder HiFi kit (New England Biolabs). Small-scale DNA isolation was performed with the QIAprep Spin Miniprep Kit (Qiagen). Vector backbones were acquired from the following sources: pRSET was acquired from Life Technologies; pGP-AAV-syn-jGCaMP7f-WPRE was a gift from D. Kim (GENIE project, Janelia Research Campus, Howard Hughes Medical Institute, Ashburn, VA, USA) and the GENIE project (Addgene plasmid 104488); pAAV-CaMKIIa-EGFP was a gift from B. Roth (Department of Pharmacology, University of North Carolina at Chapel Hill School of Medicine, Chapel Hill, NC, USA; Addgene plasmid 50469); p10xUAS-IVS-Syn21-Voltron-p10 was from a previously deposited Addgene plasmid (Addgene plasmid 119041); pTol2-HuC(*elavl3*)-CaMPARI2 was from a previously deposited Addgene plasmid (Addgene plasmid 137185); piGECI-N1 was a gift from V. Verkhusha (Department of Genetics, Albert Einstein College of Medicine, Bronx, NY, USA; Addgene plasmid 160421); pBAD-iGECInano was a gift from V. Verkhusha (Addgene plasmid 186189). pCAGGS was used from an in-house source. Cloning was conducted by PCR amplification (pDUET, pRSET) or restriction enzyme digestion of vector backbones. For use in mammalian cell culture, inserts were cloned into pCAGGS at the AflII and AgeI restriction sites. For use in rodent neurons, the inserts were cloned into pAAV-hsyn or pAAV-CaMKII at the BamHI and HindIII restriction sites. For expression in *Danio rerio* pTol2 with the HuC *elavl3* promoter, the inserts were cloned into the AgeI restriction site. For expression in *Drosophila melanogaster*, the insert was cloned into 10XUAS-IVS-Syn21 at the XhoI and XbaI restriction sites.

Inserts were amplified by PCR. WHaloCaMP constructs were cloned with a nuclear exclusion signal (NES) to mimic the cellular localization of jGCaMPs in neurons. Vector backbones and inserts were assembled by isothermal assembly with an overlap of 10–30 bp, and the sequence was verified by Sanger sequencing (Azenta Life Sciences) or by nanopore full-plasmid sequencing (Plasmidsaurus). For large-scale plasmid preparation, DNA was isolated by the Janelia Molecular Biology Facility. AAVs were prepared by Janelia Virus Services. Plasmids generated in this work have been deposited with Addgene: pRSET-WHaloCaMP1a-EGFP (205303), pRSET-WHaloCaMP1a (205304), pRSET-WHaloCaMP1b-EGFP (205305), pRSET-WHaloCaMP-eNOSpep-EGFP (205306), pAAV-synapsin-WHaloCaMP1a-EGFP (205307), pAAV-synapsin-WHaloCaMP1a (205308), pAAV-CaMKII-WHaloCaMP1a-EGFP (205309), pAAV-CaMKII-WHaloCaMP1a (205310), pTol2-elavl3-WHaloCaMP1a-EGFP (205311), pTol2-elavl3-WHaloCaMP1a (205312), pCAG-WHaloCaMP1a-EGFP (205313) and p10xUAS-WHaloCaMP1a-EGFP (205314) .

### Protein expression and purification

For expression and purification of proteins, T7 Express cells (New England Biolabs) were transformed with pRSET plasmids encoding the protein of interest. The bacteria were grown in auto-induction medium using the Studier method^45^ with antibiotics at 30 °C for 48 h, shaking at 200 rpm. Cell pellets were collected by centrifugation and lysed in Tris-buffered saline (TBS) (19.98 mM Tris, 136 mM NaCl, pH 8.0) with *n*-octyl-β-d-thioglucopyranoside (5 g l^−1^). Aggregates were disrupted by sonication, and the lysate was cleared by centrifugation. Protein purification was performed using an N-terminal poly-histidine (His_6_) tag using HisPur Ni-NTA resin (Thermo Fisher Scientific) according to the manufacturer’s recommendations. Purified proteins were buffer exchanged into TBS using Amicon concentration filters (Merck). Protein aliquots were stored at 4 °C or flash frozen and stored at −80 °C until assayed.

### Polyacrylamide gel electrophoresis

To verify protein expression and covalent bond formation with HaloTag dye-ligand, proteins were run via sodium dodecyl sulfate–polyacrylamide gel electrophoresis. Proteins were prelabeled with a 1.2-molar equivalence of dye-ligands before being separated on a NuPAGE 4–12%, Bis-Tris protein gel (Thermo Fisher Scientific). Imaging of in-gel fluorescence was performed using a ChemiDoc MP system (Bio-Rad) with epi-blue (460–490 nm), epi-green (520–545 nm) or epi-red (625–650 nm) excitation, and emission filters were set to 518–546 nm, 577–613 nm or 675–725 nm. PageBlue Protein Staining Solution (Thermo Fisher Scientific) was used to visualize the total protein amount in the gel.

### Crystallography

For crystallization, HaloTag7 was further purified with a size-exclusion chromatography Superdex 200 10/300 GL column (GE Healthcare) at a flow rate of 0.5 ml min^−1^ in 50 mM Tris-HCl, 75 mM NaCl, pH 7.4. The purified protein was incubated with 2 molar equivalents of the JF_669_-HaloTag ligand for 5 h at 22 °C (room temperature), and excess dye-ligand was removed with PD MiniTrap desalting columns with Sephadex G-25 resin (Cytiva Life Sciences), pre-equilibrated with 50 mM Tris-HCl and 75 mM NaCl, pH 7.4. Crystallization was performed at 22 °C (room temperature), and crystals were grown using the sitting-drop vapor-diffusion method in 96-well plates using precipitant solutions from Crystal Screen Cryo HT (Hampton Research). HaloTag7_669_ (10 mg ml^−1^) was crystallized by mixing 1:1 (vol:vol) with a precipitant solution of 0.2 M ammonium phosphate monobasic, 0.1 M Tris, pH 8.5, 50% (vol/vol) (+/−)-2-methyl-2,4-pentanediol. Blue HaloTag7_669_ crystals were plunged into liquid nitrogen for storage and transport. X-ray diffraction data were collected at beamline 8.2.2 at the Advanced Light Source, and a wavelength of 1 Å and a temperature of 100 K under a cold nitrogen stream were used. Diffraction data were integrated using xia2 (ref. ^46^) and DIALS^47^ and scaled using Scala from the CCP4 suite^48^. The structure was solved using Phaser^49^, using a prior structure of HaloTag7 bound to the trimethyl rhodamine HaloTag7 ligand as a search module (PDB 6U32). Refinement was performed iteratively using REFMAC5 (ref. ^50^), and model rebuilding and adjustments were performed in Coot^51^. The refined structure of HaloTag7_669_ (PDB 8SW8) exhibited good model geometry, with 94.9% of residues in the favored regions of the Ramachandran plot and 1% in disallowed regions.

### Library construction

For directed evolution of WHaloCaMPs, the gene of interest fused to the sequence for EGFP as an expression marker was cloned in the desired geometry into the pRSET plasmid. NNS degenerate codons were used for single-site mutagenesis, and primers designed using the 22-codon trick^52^ were used for two-site mutagenesis. PCR was used to amplify the gene fragment including the degenerate codon site(s), and the NEBuilder HiFi kit (New England Biolabs) was then used according to the manufacturer’s instructions. The reactions were diluted 1:3 before being electroporated into T7 Express *Escherichia coli*, which was plated on LB–ampicillin plates to isolate single colonies. Around 10–20 colonies were sequenced from each reaction to verify library generation.

### Library bacterial lysate screen

For library expression, single colonies were picked and transferred to liquid growth medium in 96-well blocks containing 800 µl auto-induction medium and ampicillin (100 µg ml^−^^1^). The blocks were shaken at 400 rpm for 48 h. The bacteria were then pelleted and lysed with three freeze–thaw cycles at −20 °C or −80 °C. The pellet was resuspended in 30 mM 3-morpholinopropane-1-sulfonic acid (MOPS), 100 mM KCl at pH 7.2, with shaking for ~1 h at 37 °C, and the lysate was cleared by centrifugation. After clearing, 250 µl of the lysate was transferred into 96-well blocks of 10-µl pre-aliquoted solutions of 10 µM JF_669_-HaloTag ligand in 30 mM MOPS, 100 mM KCl at pH 7.2 (final labeling concentration of the JF_669_-HaloTag ligand was ~380 nM). Two 96-well black plates were prepared with a ‘low’ concentration of Ca^2+^ (5 µl, 10 mM CaCl_2_ in 30 mM MOPS, 100 mM KCl at pH 7.2) or a ‘low’ concentration of ethylene glycol-bis(β-aminoethyl ether)-*N*,*N*,*N*′,*N*′-tetraacetic acid (EGTA) (5 µl, 20 mM EGTA in 30 mM MOPS, 100 mM KCl at pH 7.2). The JF_669_-dye labeled lysate (95 µl) was transferred into the prepared 96-well plates (final concentration of low Ca^2+^ was 0.5 mM, and low EGTA was 1 mM), and fluorescence was read on a plate reader (Tecan Spark 20M) with a stacking module. Fluorescence was read at an excitation wavelength of 485 nm and an emission wavelength of 510 nm for EGFP and at an excitation wavelength of 670 nm and an emission wavelength of 690 nm for proteins labeled with JF_669_ with a 5-nm bandgap. After an initial reading, the Ca^2+^ and EGTA conditions were interchanged: 20 µl of a solution with 100 mM EGTA was added to the 96-well plate containing Ca^2+^ at a low concentration, and 20 µl of a solution with 50 mM CaCl_2_ was added to the 96-well plate containing EGTA at a low concentration. The fluorescence emission was again read on a plate reader (Tecan Spark 20M) with a stacking module. To identify top performing variants, variants above a threshold value of EGFP fluorescence were ranked by calculating ∆*F*/*F*_0_ (where ∆*F* is the fluorescence intensity in Ca^2+^ fluorescence intensity in EGTA and *F*_0_ is the fluorescence intensity in EGTA) of the JF_669_ signal both in the ‘low’ Ca^2+^–EGTA and ‘high’ Ca^2+^–EGTA regimes. Depending on the library, 0–38 top hits were chosen, expanded from the pelleted lysate before labeling and sequenced by Sanger sequencing.

### Library Ca^2+^ response validation

Unique sequences of top performing variants were expressed and purified using their N-terminal His_6_-tag as described above. The concentration of the purified proteins was quantified using the absorbance of EGFP fused to the C terminal (*ε*_488_ = ~ 55,000 cm^−1^ M^−1^). The variants were labeled with substoichiometric amounts of dye-ligand (15 µM purified protein with 10 µM JF_669_-HaloTag ligand) for at least 1 h at 22 °C (room temperature). The labeled variants (2 µl) were aliquoted into 98 µl of 0.5 mM ‘low’ Ca^2+^ and 1 mM ‘low’ EGTA, and the fluorescence emission was read with a plate reader, recording both the EGFP and JF_669_ fluorescence emission, as previously performed. The ‘low’ conditions were then changed to ‘high’ by adding 20 µl of 100 mM EGTA to the ‘low’ Ca^2+^ wells and 20 µl of 50 mM CaCl_2_ to the ‘low’ EGTA wells, and fluorescence was read once again on the plate reader. The ∆*F*/*F*_0_ value was calculated for each variant, both in the ‘low’ Ca^2+^–EGTA and ‘high’ Ca^2+^–EGTA regimes. The overall fluorescent intensity was compared to that of the HaloTag–EGFP label and read under the same conditions in the plate reader. The variants with the largest ∆*F*/*F*_0_ value in the validation were further validated in a dye-ligand-capture assay by fluorescent polarization.

### Fluorescence polarization dye-ligand-capture rate assay

To determine the dye-ligand-capture rate of the protein variants with the dye-HaloTag ligand, a fluorescence polarization assay was used. Purified protein was diluted into buffer containing 30 mM MOPS, 100 mM KCl, pH 7.2 and 0.5 mg ml^−^^1^ BSA, with either 10 mM EGTA or 20 mM CaCl_2_. Proteins were prepared at 625 nM, 312.5 nM, 156 nM and 78 nM. The diluted solutions (80 µl) were added to 96-well black-well plates. A solution of the JF_549_-HaloTag ligand was prepared in 30 mM MOPS, 100 mM KCl, pH 7.2, 0.5 mg ml^−^^1^ BSA, 10 mM EGTA at a final concentration of 62.5 nM. Fluorescence polarization was read over 5–20 min in a plate reader (Tecan Spark 20M) at 37 °C, after mixing 80 µl of the protein solutions with 20 µl of the dye-ligand solution with final protein concentrations of 500 nM, 250 nM, 125 nM and 62.5 nM and a final dye-ligand concentration of 12.5 nM. Excitation was set to 535 nm, and emission was set to 580 nm, with a bandwidth of 20 nm. The G factor was manually set to 1.79 after measuring a dilute concentration of dye-ligand in buffer with the same excitation and emission settings. The binding curve over time was fit with a single exponential in a custom-written Python script with curve_fit in scipy.optimize, where *y* is the fluorescence polarization in mP, *Y*_0_ is the value of *Y* when intercepting the *y* axis, Pl is the value of *Y* at plateau, *k* is the pseudo-first-order rate constant and *x* is the time in seconds.

$$y\left(x\right)={Y}_{0}+\left({\rm{Pl}}-\,{Y}_{0}\right)\times (1-{e}^{-{kx}})$$

The pseudo-first-order rate constant was calculated for each protein concentration, and an apparent or calculated second-order rate constant for dye-ligand capture was calculated, where *k*_2,calc_ is the calculated second-order rate constant in M^−1^ s^−1^, *k* is the pseudo-first-order rate constant and [protein] is the protein concentration during the run.

$${k}_{2,{\rm{calc}}}=\frac{k}{[{\rm{protein}}]}\,$$

This plate reader assay was too slow to capture rates faster than 10^6^ M^−1^ s^−1^; however, we used it to verify that we had not lost the fast dye-ligand-capture rate for the variants.

### General experimental information for synthesis

See Supplementary Note 2.

### Ultraviolet–visible spectroscopy of protein–dye conjugates

To record absorbance spectra and molar extinction coefficients of WHaloCaMP bound to dyes, proteins were prelabeled with dye-HaloTag ligand. The dye-HaloTag ligand was reconstituted from lyophilized powder at 2 mM in dimethylsulfoxide (DMSO). For excess dye-ligand addition and subsequent removal, HaloTag or variants of WHaloCaMP were diluted to 50 µM in TBS, and dye-HaloTag ligand was added at a final concentration of 100 µM. After an incubation of at least 1 h at 22 °C (room temperature), excess dye-ligand was removed with a PD SpinTrap G-25 column (Cytiva Life Sciences), pre-equilibrated with 30 mM MOPS, 100 mM KCl at pH 7.2. After elution, the protein–dye concentration was quantified with a NanoDrop One^C^ UV–Vis spectrophotometer (Thermo) by reading the absorbance at 280 nm and using a calculated molar extinction coefficient at 280 nm (Expasy, ProtParam tool). Protein concentrations were around 40 µM. For substoichiometric labeling of the protein variants, 20 µM protein was diluted in TBS and labeled with 10 µM JF dye-HaloTag ligand. DMSO concentrations did not exceed 1% (vol/vol). The protein–dye conjugates were diluted to 1.5 µM in the two solutions from Calcium Calibration Buffer Kit #1 (Invitrogen) (0 and 10 mM CaEGTA, containing either 10 mM EGTA or 39 µM free Ca^2+^ in 30 mM MOPS, 100 mM KCl at pH 7.2). Absorption spectra were recorded on a Cary Model 100 spectrometer (Agilent) using 1-cm-pathlength semi-micro quartz cuvettes with self-masking sides (1-ml volume). Spectra were normalized to the maximum absorption in the presence of saturating Ca^2+^. For molar extinction measurements, protein–dye conjugates were further diluted in with the 0 mM or 10 mM CaEGTA buffer to 750 nM, 375 nM and 187 nM, and the absorbance spectra were recorded. The molar extinction coefficient at a particular wavelength was determined by performing a background correction and plotting the absorbance (*y*) against the concentration (*x*). The molar excitation coefficient (*b*) was taken to be the line fit through the points, with a *y*-intercept of *a*.

$$y\,\left(x\right)=a+{bx}$$

### Fluorescence spectroscopy of protein–dye conjugates

To record fluorescence excitation and emission spectra, proteins were first prelabeled with dye-HaloTag ligand at a substoichiometric molar ratio. Protein (20 µM) was diluted in TBS and labeled with 10 µM JF dye-HaloTag ligand. DMSO concentrations did not exceed 1% (vol/vol). After incubation for at least 1 h at 22 °C (room temperature), the protein–dye conjugate was diluted in either 0 mM or 10 mM CaEGTA buffer. The final concentration of the protein–dye conjugate was 666 nM. Fluorescence spectra were recorded on a Cary Eclipse fluorometer (Agilent) using 1-cm-pathlength quartz spectrophotometer 3.5-ml cuvettes. Spectra were normalized to the maximum fluorescence excitation or emission in the presence of saturating Ca^2+^ concentrations.

### Two-photon spectroscopy of protein–dye conjugates

WHaloCaMP1a was prelabeled with a substoichiometric amount of dye (10 µM dye-ligand to 20 µm WHaloCaMP1a) for at least 1 h at room temperature. The protein–JF dye-ligand conjugates were then diluted to a final concentration of 1 µM of dye-ligand equivalence in either 0 mM or 10 mM CaEGTA buffers from Calcium Calibration Buffer Kit #1 (Invitrogen). Two-photon spectroscopy of WHaloCaMP–JF dye-ligand conjugates was performed as previously described^11,53^. Briefly, an inverted microscope (IX81, Olympus) equipped with a ×60 1.2-numerical aperture (NA) water objective (Olympus) was used. To excite the samples, a pulsed 80-MHz Ti–sapphire laser (Chameleon Ultra II, Coherent) was used for 710–1,080 nm and an OPO (Chameleon Compact OPO, Coherent) was used for 1,000–1,500 nm. For excitation of WHaloCaMP1a_494_ and WHaloCaMP1a_552_, a dichroic filter (675DCSXR, Omega) and a shortpass filter (720SP, Semrock) were used, and emission was collected using a bandpass filter (539BP278, Semrock). By contrast, for WHaloCaMP1a_669_ and WHaloCaMP1a_722_, laser excitation was achieved through a dichroic filter (FF825-SDio1, Semrock) and emission was collected using a bandpass filter (709BP167, Semrock). Fluorescence detection was achieved with a fiber-coupled avalanche photodiode (SPCM_AQRH-14, PerkinElmer). All the fluorescence excitation spectra are corrected for the wavelength-dependent transmission of the dichroic and bandpass filters and quantum efficiency of the detector. The corrected spectra were used to calculate the action cross-section using fluorescein^54^, rhodamine B^55^ and styryl 9M^55^ as a reference. Spectra are averages (*n* = 2).

### Quantum yield determination of protein–dye conjugates

To record quantum yields, proteins were first prelabeled with JF dye-HaloTag ligand at a substoichiometric molar ratio. The protein–dye-ligand conjugates were diluted in either 0 mM or 10 mM CaEGTA buffer. A Quantaurus-QY spectrometer (model C11374, Hamamatsu) with an integrating sphere was used, measurements were performed on dilute samples (*A* < 0.1), and self-absorption corrections were performed^56^ using the instrument software.

### Ca^2+^ titrations

To determine the Ca^2+^ affinity and cooperativity of the WHaloCaMP sensors, Ca^2+^ titrations were performed with a commercial Calcium Calibration Buffer Kit #1 (Invitrogen) with 0 mM and 10 mM CaEGTA. Proteins were prelabeled with excess dye-HaloTag ligand, and any excess dye-ligand was removed with a PD SpinTrap G-25 column (Cytiva Life Sciences), pre-equilibrated with 30 mM MOPS, 100 mM KCl at pH 7.2. The prelabeled protein–dye conjugate (2 µl) was diluted into 98 µl of a premixed solution of CaEGTA in black 96-well plates. Fluorescence intensities were read in a plate reader (Tecan Spark 20M). For WHaloCaMP1a_494_, excitation was 490 nm and emission was 530 nm. For WHaloCaMP1a_552_, excitation was 560 nm and emission was 580 nm. For WHaloCaMP1a_669_, excitation was 670 nm and emission was 690 nm. For WHaloCaMP1a_722_, excitation was 730 nm and emission was 750 nm. All bandwidths were set to 5 nm. The free Ca^2+^ concentration was calculated, taking the dissociation constant of EGTA for Ca^2+^ to be 150 nM at 22 °C and pH 7.2 (ref. ^57^). Changes in fluorescence on addition of Ca^2+^ were calculated in Microsoft Excel. The fluorescence (*y*) was plotted against the free Ca^2+^ concentration (*x*), and a four-parameter dose–response curve (variable slope) using GraphPad Prism software was fit in which *a* is the value of fluorescence at the bottom of the curve, *b* is the value of fluorescence at the top of the curve, EC_50_ is the concentration of agonist that gives a response halfway between the bottom and the top, and *h* is the Hill or cooperative coefficient.

$$y\,(x)=a+\frac{{x}^{h}\times (b-a)}{{x}^{h}+\,{{{\rm{EC}}}_{50}}^{h}}\,$$

### pH sensitivity of probes in vitro

Proteins were prelabeled with dye-HaloTag ligand at a substoichiometric molar ratio: 20 µM protein was diluted in TBS and labeled with 10 µM dye-HaloTag ligand. Labeled protein was then diluted into the following buffer systems containing 1 mM CaCl_2_ or 50 mM EGTA: citrate (pH 4.0–6.2), phosphate (pH 5.8–8.0) and Tris (pH 7.8–9.0), all with 10 mM buffer and 150 mM NaCl. Protein (2 µl) was diluted into the buffer in 96-well black plates, and fluorescence was read with a plate reader (Tecan Spark 20M).

### Stopped-flow kinetic measurements of Ca^2+^ kinetics

Ca^2+^ unbinding kinetics of WHaloCaMPs were determined using a Photophysics SX20 stopped-flow device. The prelabeled protein (~1.3 µM) was rapidly mixed with a solution of 30 mM MOPS, 100 mM KCl, 10 mM EGTA, pH 7.2 in a 1:1 ratio. The sensors were excited using a light-emitting diode (LED; 625 nm), and fluorescence emission was collected through a 665-nm longpass filter. Fluorescence decay was fit with the decay curves described in Supplementary Fig. 2.

### One-photon widefield bleaching of WHaloCaMP1a_669_

One-photon bleaching experiments were performed with an inverted Nikon Eclipse Ti2 microscope as previously described^58^. Briefly, bleaching experiments were carried out in aqueous droplets of purified protein labeled with dye-ligand isolated in 1-octanol^59^. Labeled purified protein was diluted to 500 nM using the commercial Calcium Calibration Buffer Kit #1 (Invitrogen) with 0 mM and 10 mM CaEGTA and supplemented with 0.3 mg ml^−^^1^ BSA. This solution was diluted tenfold in a 1-octanol–PBS suspension and agitated by tapping. The solution was placed on a pre-silanized glass slide and sandwiched with a coverslip. The microdroplets were illuminated using a ×40 (NA = 1.3, Plan Fluor, Nikon (field number (FN) = 25.2)) oil-immersion objective. Illumination was provided by an LED (Spectra X Light Engine, Lumencor) and imaged through the following filter cube (89000, Chroma), ET645/30x, 89100bs, ET705/72m. Images were captured with a scientific complementary metal-oxide semiconductor (sCMOS) camera (ORCA-Flash4.0, Hamamatsu), and images were acquired with 1 s of exposure every 2 s. Power at the objective was measured with a microscope slide power sensor (S170C, Thorlabs) to 7 mW, giving an irradiance of 23 mW mm^−^^2^. Each sample was bleached continuously for 6.6 min. Background subtraction was performed in Fiji^60^; fluorescence emission was normalized to the first frame. One-phase decay was fit to the data in GraphPad Prism software.

### Primary neuronal culture and labeling with dye-HaloTag ligand

Procedures involving rodent animals were conducted in accordance with protocols approved by the Howard Hughes Medical Institute Janelia Research Campus Institutional Animal Care and Use Committee and Institutional Biosafety Committee. Hippocampal neurons were extracted from P0–P1 Sprague-Dawley rat pups and plated into 24-well glass-bottom plates or 35-mm dishes (MatTek, #1.5 coverslip). A coating of poly-d-lysine was applied before plating the cells. Neurons were cultured at 37 °C with 5% CO_2_ in a humidified atmosphere in NbActiv4 medium (BrainBits). After plating (3–4 d), neurons were transduced with AAV1 virus to express the gene of interest under the human SYN1 (*hsyn1*) promoter. After transduction (17–21 d), the neurons were labeled with dye-HaloTag ligand. Dye-HaloTag ligands were first dissolved to make 2 mM stock solutions in DMSO from lyophilized powder. The neurons were labeled with 50–100 nM dye-HaloTag ligand at 37 °C for 30–45 min. The neurons were then washed with imaging buffer containing 145 mM NaCl, 2.5 mM KCl, 10 mM glucose, 10 mM HEPES (pH 7.4), 2 mM CaCl_2_ and 1 mM MgCl_2_. After three washes, imaging buffer containing synaptic blockers^61^ (10 μM 6-cyano-7-nitroquinoxaline-2,3-dione, 10 μM 3-(2-carboxypiperazin-4-yl)propyl-1-phosphonic acid, 10 μM gabazine and 1 mM (*S*)-α-methyl-4-carboxyphenylglycine) was added to block ionotropic glutamate, γ-aminobutyric acid and metabotropic glutamate receptors.

### Field stimulation of WHaloCaMP1a in primary neuronal culture

Field stimulation of neurons was performed using a platinum wire electrode inserted in the medium, controlled by a high-current isolator (A385, World Precision Instruments) set at 90 mA. Trains of APs were delivered to the platinum wire electrode from one to 160 APs, at intervals described in each imaging modality. Unless otherwise stated, the fluorescent traces from hand-segmented neurons from at least three fields of view from at least two separate neuron batches were extracted in Fiji^60^. ∆*F*/*F*_0_ values were calculated, with *F*_0_ being the average fluorescence recorded 1 s before each stimulus. Cells with ∆*F*/*F*_0_ > 0 were used. The signal-to-noise ratio was calculated from the amplitude in the fluorescent change on the field stimulation divided by the standard deviation of the baseline fluorescence 1 s before each stimulus. Calculated time to peak was calculated from the onset of stimulus to the maximal fluorescence value. The *t*_1/2_ of decay was calculated by fitting a two-phase decay in GraphPad Prism software to the ∆*F*/*F*_0_ trace after the field stimulus had stopped.

### Widefield imaging in the visible spectrum

Widefield imaging in the visible spectrum (up to 700 nm) was performed on an inverted Nikon Eclipse Ti2 microscope with a ×20 air objective (NA = 0.75, Nikon) equipped with a Spectra X Light Engine (Lumencore) and imaged with an ORCA-Flash4.0 sCMOS camera (Hamamatsu). The Spectra X Light Engine was equipped with 485/25-nm, 550/15-nm and 640/30-nm excitation filters for exciting WHaloCaMP_494_, WHaloCaMP_552_ and WHaloCaMP_669_. A quad bandpass (set number 89000, Chroma) with 490/20-nm, 555/25-nm and 645/30-nm excitation filters, a dichroic mirror (89100bs, Chroma) and 525/36-nm, 605/52-nm and 705/72-nm emission filters (Chroma) were also used. Imaging was performed at 33 Hz, and the field stimulation was controlled with an Arduino Uno board synchronized with light sources in Nikon Elements software. A train of 1, 2, 3, 5, 10, 20, 40, 80, 120 and 160 APs with a pulse duration of 1 ms at 80 Hz were elicited with 20-s time intervals between each stimulation.

### Widefield imaging in the near-infrared

For imaging WHaloCaMP1a_722_ in the near-infrared, we used a custom-built upright microscope as previously described^62^ with modifications. Neurons expressing WHaloCaMP1a and labeled with the JF_722_-HaloTag ligand were imaged using a ×40 objective (Nikon N40X-NIR, 0.8 NA and a working distance of 3.5 mm). To excite WHaloCaMP1a_722_, we used a 671-nm laser (gem 671, Laser Quantum). To image the EGFP expression marker, we used a four-wavelength LED light source (Thorlabs, LED4D067), using the LED at 470 nm. We used an imaging dichroic beam splitter with edge at 699 nm (FF699-FDI01-T1-25x36, Semrock) and a 715-nm emission filter (FF01-715/LP-25, Semrock) to clean up the fluorescence signal before detection by camera. Both visible and near-infrared fluorescence signals were imaged using an InGaAs camera with optimized sensitivity in the NIR–SWIR range (Ninox 640 II, Raptor Photonics). Images were acquired using µManager, an open-source microscopy software^63^. Images were acquired at 10 frames per second with 400–500 frames for each run. We performed field stimulation with the stimulation controlled using the MATLAB-based waveform generator WaveSurfer to elicit 1, 5, 10, 20 or 160 APs with a pulse duration of 1 ms and a square pulse train at 80 Hz and analyzed the data as described under ‘Field stimulation of WHaloCaMP1a in primary neuronal culture’.

### Simultaneous electrophysiology and fluorescence imaging in primary neuron culture

All imaging and electrophysiology measurements were performed in imaging buffer. Internal solution for current-clamp recordings contained the following: 130 mM potassium methanesulfonate, 10 mM HEPES, 5 mM NaCl, 1 mM MgCl_2_, 1 mM magnesium ATP, 0.4 mM sodium GTP and 14 mM Tris-phosphocreatine, adjusted to pH 7.3 with KOH and adjusted to 300 mOsm with sucrose. Glass capillaries with filaments (Sutter Instrument) were pulled to a tip resistance of 4–6 MΩ.

Pipettes were positioned with an MPC-200 manipulator (Sutter Instrument). An EPC 800 amplifier (HEKA) was used for acquiring electrophysiology recordings, filtered at 10 kHz with an internal Bessel filter and digitized using a National Instruments PCIe-6353 acquisition board at 20 kHz. Data were acquired from cells with access resistance <25 MΩ. WaveSurfer software was used to generate the various analog and digital waveforms to control the amplifier, the camera and light source and record voltage and current traces. Cells were initially held at −70 mV. A 440-nm light pulse (5 ms, 10 Hz at 0.45 mW mm^−2^ when CheRiff was coexpressed with WHaloCaMP1a) or current injection (when only WHaloCaMP1a was expressed) was applied to evoke APs and initiate Ca^2+^ entry into the cell.

Light (640 nm at 0.76 mW mm^−^^2^) was used to excite WHaloCaMP1a with an emission filter at 705/72 nm. Light (555 nm at 1.39 mW mm^−^^2^) was used for excitation of jRCaMP1a, and emitted fluorescence was filtered with a 607/70-nm emission filter. Fluorescence images were collected using an sCMOS camera (ORCA-Flash4.0, Hamamatsu), and image acquisition was performed using HCImage Live (Hamamatsu) with frame rate at 30 Hz.

### Current-clamp recording of CheRiff with different wavelengths of light

For testing CheRiff’s response to longer wavelength stimulation, 555-nm and 640-nm light was applied for 1 s to illuminate CheRiff-expressing neurons, and voltage signals were monitored. Light powers were measured using a power meter (Thorlabs, PM100A) with an Si photodiode (Thorlabs, S120C or S170C).

### Two-photon imaging of acute mouse slices with WHaloCaMP1a

All procedures for rodent husbandry and surgery were performed following protocols approved by the Washington University Institutional Animal Care and Use Committee and in accordance with National Institutes of Health guidelines. The sequence for FLIM-AKAR, together with a transcriptional stop codon flanked by *loxP* sites, was knocked in at the ROSA26 locus (JAX 039003)^26^. These mice were subsequently crossed with *Emx1*^IRES-Cre^ mice (JAX 005628)^64^ to create mice homozygous for both *Emx1*-Cre and the sequence for FLIM-AKAR at their respective loci. We did not select for males or females specifically for the experiment. Mice were kept on a 12-h light–dark cycle, with humidity between 30% and 70% and a temperature of 20–26 °C.

WHaloCaMP1a was delivered via stereotaxic injections. AAV1-CAMKII-NES-WHaloCaMP1a (5 × 10^12^ virus molecules per ml) was delivered using a UMP3 microsyringe pump (World Precision Instruments) via glass pipette to P0 or P1 pups. A total of 200 nl per pup was delivered at ten different coordinates (20 nl per coordinate) at a rate of 20 nl s^−1^. The coordinates are as follows, relative to lambda in millimeters: (1) anterior, 1.2; lateral, 1.2; depth from the skin, 1.2, 0.8, 0.4, (2) anterior, 0.8; lateral, 1.5; depth from the skin, 1.5, 1.0, 0.5, and (3) anterior, 0.5; lateral, 2.0; depth from the skin, 2.0, 1.5, 1.0, 0.5.

The JF_669_-HaloTag ligand fluorescent dye was delivered either by r.o. injection into mice before acute slicing or by incubation of acute brain slices (from dye-naive mice) in dye-infused artificial cerebrospinal fluid (ACSF; see composition below). For r.o. injection, a total of 200 nmol JF_669_-HaloTag ligand was first dissolved in 20 µl DMSO and subsequently diluted with 20 µl of 20% Pluronic in DMSO (Fisher) and 90 µl PBS (Corning). Imaging was performed within 24 h of dye injection. For acute slice incubation, 100 nmol JF_669_-HaloTag ligand was reconstituted in 20 µl DMSO and mixed with ACSF to create a 0.0626–0.25 μM solution. Acute brain slices were incubated in this dye–ACSF solution for 30 min and were subsequently washed with dye-free ACSF for at least 30 min before imaging.

Mice were anesthetized with isoflurane and subsequently euthanized. All solutions were bubbled with carbogen (5% CO_2_ and 95% O_2_) before use, and ACSF was continuously bubbled while in use. Mice aged P25 or younger were sectioned in cold sucrose cutting solution (containing (in mM): 87 NaCl, 25 NaHCO_3_, 1.25 NaH_2_PO_4_, 2.5 KCl, 75 sucrose, 25 glucose, 1 MgCl_2_). Mice aged P30 or older underwent intracardiac perfusion with cold ACSF (containing (in mM): 127 NaCl, 2.5 KCl, 25 NaHCO_3_, 1.25 NaH_2_PO_4_, 2 CaCl_2_, 1 MgCl_2_ and 25 glucose). Their brains were rapidly dissected and sectioned in cold choline cutting solution (containing (in mM): 25 NaHCO_3_, 1.25 NaH_2_PO_4_, 2.5 KCl, 7 MgCl_2_, 25 glucose, 0.5 CaCl_2_, 110 choline chloride, 11.6 ascorbic acid, 3.1 pyruvic acid). Acute coronal slices were collected at a thickness of 300 μm with a Leica VT1000 S vibratome.

After sectioning, slices from both groups were transferred to ACSF for recovery at 34 °C for 5–10 min. After recovery, the slices were kept in ACSF at room temperature. Once ready for imaging, the slices were transferred to a microscope chamber where they were perfused with ACSF at a flow rate of 2–4 ml min^−1^.

A custom-built microscope was used to perform two-photon imaging in acute brain slices. WHaloCaMP1a_669_ was excited at a wavelength of 1,250 nm, and FLIM-AKAR was excited at 920 nm. Two mode-locked laser sources were used in an alternating fashion to excite WHaloCaMP1a_669_ (Spectra-Physics, InSight X3, 80 MHz) and FLIM-AKAR (Spectra-Physics, Mai Tai HP Ti:Sapphire, 80 MHz), respectively. Photons were collected with fast photomultiplier tubes (PMTs; Hamamatsu, H10770PB-40) with a ×60 objective (Olympus, NA 1.1). Image acquisition was accomplished with ScanImage^65^, a custom-written software, in MATLAB 2012b.

FLIM was performed as described previously with FLIM-AKAR^24–26^. The FLIM board used was SPC-150 (Becker & Hickl), and time domain single-photon counting was performed in 256 time channels. Intensity Ca^2+^ imaging was performed with WHaloCaMP1a_669_. Neurons with nuclear-excluded basal WHaloCaMP1a_669_ signals were used for experiments. Pixel images (128 × 128) were collected.

Both sensors’ emission light was collected through a 580-nm dichroic mirror (FF580-FDi01-25x36, Semrock), followed by a 525/50-nm bandpass filter (FF03-525/50-25, Semrock) for FLIM-AKAR or a 690/50-nm bandpass filter (ET690/50m, Chroma) for WHaloCaMP1a_669_.

All acute slice experiments were performed in the presence of 1 μM DPCPX to inhibit adenosine receptors and 1 μM tetrodotoxin to block APs.

All chemicals were added to acute slices by addition into the perfusion reservoir. The final concentrations of chemicals are as follows: (+)-muscarine iodide (Mus), 10 μM, from Tocris; tetrodotoxin citrate, 1 μM, from Hello Bio; DPCPX, 1 μM, from Tocris; forskolin (FSK), 50 μM, from Cayman Chemical; potassium chloride, 55 mM, from Cayman Chemical.

The ROI for each neuronal cell body was manually selected. An ROI for background fluorescence with no cellular processes was similarly selected. The fluorescence signal for all pixels in a neuron’s ROI was averaged, and the average background fluorescence was subtracted from it. The resulting net fluorescence was plotted against time. The Δ*F*/*F*_0_ was calculated as (*F* − *F*_0_)/*F*_0_, where *F*_0_ is the fluorescence signal averaged over the entire baseline period.

The amplitudes of intensity changes were quantified as follows:

$${\rm{Baseline}}_{\rm{start}}={\rm{average}}\,\Delta {{F}/{F}}_{0}\,{\rm{of}}\,{\rm{first}}\,{\rm{three}}\,{\rm{intensity}}\,{\rm{acquisitions}}\,{\rm{of}}\,{\rm{baseline}};$$

$${\rm{Baseline}}_{\rm{end}}={\rm{average}}\,\Delta {{F}/{F}}_{0}\,{\rm{of}}\,{\rm{last}}\,{\rm{three}}\,{\rm{intensity}}\,{\rm{acquisitions}}\,{\rm{of}}\,{\rm{baseline}};$$

$$\begin{array}{l}{\rm{Mus}}_{\rm{end}}={\rm{average}}\,\Delta {{F}/{F}}_{0}\,{\rm{of}}\,{\rm{last}}\,{\rm{three}}\,{\rm{intensity}}\,{\rm{acquisitions}}\,\\\qquad\qquad{\rm{of}}\,{\rm{muscarine}}\hbox{-}{\rm{only}}\,{\rm{application}};\end{array}$$

$${\rm{Baseline}}_{\rm{max}}=\,{\rm{maximum}}\, \Delta {{F}/{F}}_{0}\,{\rm{recorded}}\,{\rm{during}}\,{\rm{baseline}};$$

$${\rm{Mus}}_{\rm{max}}=\,{\rm{maximum}}\, \Delta {{F}/{F}}_{0}\,{\rm{recorded}}\,{\rm{during}}\,{\rm{muscarine}}\hbox{-}{\rm{only}}\,{\rm{application}};$$

$${\rm{FSK}}_{\rm{max}}=\,{\rm{maximum}}\, \Delta {{F}/{F}}_{0}\,{\rm{recorded}}\,{\rm{during}}\,{\rm{forskolin}}\,{\rm{application}};$$

$${\rm{Max}}\,\Delta {\rm{Baseline}}={\rm{MaxBaseline}}-{\rm{Baseline}}_{\rm{start}};$$

$${\rm{Max}}\,\Delta {\rm{Mus}}={\rm{Mus}}_{\rm{max}}-{\rm{Baseline}}_{\rm{end}};$$

$${\rm{Max}}\,\Delta {\rm{FSK}}={\rm{FSK}}_{\rm{max}}-{\rm{Mus}}_{\rm{end}}.$$

For presentation purposes, the WHaloCaMP1a_669_ line was smoothened and presented alongside the raw data. Smoothing was performed in GraphPad Prism, using six neighboring points and a second-order polynomial for smoothing.

FLIM curve fitting, calculation of average lifetime over an ROI and ROI analysis were performed as described previously^25^. The amplitudes of cytoplasmic lifetime changes were quantified as follows:

$${\rm{Baseline}}_{\rm{start}}={\rm{average}}\,{\rm{lifetime}}\,{\rm{of}}\,{\rm{the}}\,{\rm{first}}\,{\rm{three}}\,{\rm{acquisitions}}\,{\rm{of}}\,{\rm{baseline}};$$

$${\rm{Baseline}}_{\rm{end}}={\rm{average}}\,{\rm{lifetime}}\,{\rm{of}}\,{\rm{the}}\,{\rm{last}}\,{\rm{three}}\,{\rm{acquisitions}}\,{\rm{of}}\,{\rm{baseline}};$$

$${\rm{Baseline}}_{\rm{min}}={\rm{minimum}}\,{\rm{lifetime}}\,{\rm{during}}\,{\rm{baseline}};$$

$$\begin{array}{l}{\rm{Mus}}_{\rm{end}}={\rm{average}}\,{\rm{lifetime}}\,{\rm{of}}\,{\rm{the}}\,{\rm{last}}\,{\rm{three}}\,\\\qquad\qquad{\rm{acquisitions}}\,{\rm{of}}\,{\rm{muscarine}}\,{\rm{only}}\,{\rm{application}};\end{array}$$

$${\rm{Mus}}_{\rm{min}}={\rm{minimum}}\,{\rm{lifetime}}\,{\rm{during}}\,{\rm{muscarine}}\,{\rm{only}}\,{\rm{application}};$$

$${\rm{FSK}}_{\rm{min}}={\rm{minimum}}\,{\rm{lifetime}}\,{\rm{during}}\,{\rm{forskolin}}\,{\rm{application}};$$

$${\rm{Max}}\,\Delta {\rm{Baseline}}={\rm{Baseline}}_{\rm{min}}-{\rm{Baseline}}_{\rm{start}};$$

$${\rm{Max}}\,\Delta {\rm{Mus}}={\rm{Mus}}_{\rm{min}}-{\rm{Baseline}}_{\rm{end}};$$

$${\rm{Max}}\,\Delta {\rm{FSK}}={\rm{FSK}}_{\rm{min}}-{\rm{Mus}}_{\rm{end}}.$$

### WHaloCaMP imaging in living adult flies using widefield epifluorescence microscopy

Experiments were performed on 2–10-d-old heterozygous progeny of a cross between a mushroom body driver (R13F02-Gal4) and the two WHaloCaMP variants 10XUAS-NES::WHaloCaMP1a::EGFP-P10 in VK00005 and 10XUAS-NES::WHaloCaMP1b::EGFP-P10 in VK00005. jRGECO1a (20XUAS-IVS-NES-jRGECO1a-p10 in VK00005), which shares excitation and emission spectra with JF_552_, was used as a comparison.

Dissections of head-fixed flies and dye-ligand incubation were performed similarly as in Voltron experiments^58^. Briefly, a small window was opened in the head cuticle, and fat tissue and trachea that overlaid the mushroom body calyx region were removed. The exposed brain was bathed in a drop (~200 μl) of dye-containing saline (5 μM for JF_669_-HaloTag ligand and 1 μM for JF_552_-HaloTag ligand) for 1 h. Saline contained (in mM) NaCl, 103; KCl, 3; CaCl_2_, 1.5; MgCl_2_, 4; NaHCO_3_, 26; *N*-Tris(hydroxymethyl)methyl-2-aminoethanesulfonic acid, 5; NaH_2_PO_4_, 1; trehalose, 10; and glucose, 10 (pH 7.3 when bubbled with 95% O_2_ and 5% CO_2_), 275 mOsm. The brain was then washed with fresh saline several times and maintained in the saline for 1 h. Imaging was performed on a widefield fluorescence microscope (SOM, Sutter Instrument) equipped with a ×60, 1.0-NA, water-immersion objective (LUMPlanFl/IR, Olympus) and an sCMOS camera (ORCA-Flash4.0 version 3, Hamamatsu). Images of 256 × 256 pixels were acquired at 10 Hz with 4 × 4 binning with Hamamatsu imaging software (HCImage Live). Illumination was provided from an optical beam-combining system (LB-OBC-LLG, Sutter) equipped with a 660-nm LED (FG-OBC-660, Sutter) with an excitation filter (FF01-660/13-25-STR, Semrock) and a 561-nm LED (FG-OBC-561, Sutter) with an excitation filter (FF01-554/23-25-STR, Semrock). As the incidence angles were arranged at 18° in the four-beam combiner, the bandpass spectra of the excitation filters were shifted toward a shorter wavelength by 5–10 nm (SearchLight Spectra Viewer modeling). For WHaloCaMP_669_, the 660-nm LED was used, and intensity at the sample plane was ~0.64 mW mm^−^^2^; emission was separated from excitation light using a dichroic mirror (FF677-Di01-25/36, Semrock) and an emission filter (FF01-719/60-25, Semrock). For WHaloCaMP_552_ and jRGECO1a, the 561-nm LED was used, and intensities at the sample plane were ~0.50 mW mm^−2^ and ~0.17 mW mm^−2^, respectively; emission was separated from excitation light using a dichroic mirror (FF562-Di03-25x36, Semrock) and an emission filter (FF01-590/36-25, Semrock).

Odors used were 3-octanol (218405-50G, Sigma-Aldrich), 4-methylcyclohexanol (153095-250ML, Sigma-Aldrich) and apple cider vinegar (Heinz). Odors were diluted using a two-step, air dilution-based olfactometer^27^ to a final concentration of 1:16 and delivered to flies at 200 ml min^−1^ for 2 s.

Data were analyzed in MATLAB with a custom script. Images were registered to correct for *x*–*y* movement. ROI corresponding to the calyx were manually selected, and the mean intensity of the ROI was extracted. *F*_0_ was calculated as the mean over the 5 s of the imaging session before odor onset. Mean odor responses were integrated over a time window from 0.5 s and 5.5 s after odor onset.

### Mice

All experimental protocols at Janelia Research Campus were conducted according to the National Institutes of Health guidelines for animal research and were approved by the Institutional Animal Care and Use Committee at Janelia Research Campus, Howard Hughes Medical Institute. All experimental protocols at the University of Maryland, Baltimore, were conducted according to the National Institutes of Health guidelines for animal research and approved by the Institutional Animal Care and Use Committee at the University of Maryland, Baltimore.

For two-photon experiments with WHaloCaMP1a_669_, male mice (C57BL/6J background) aged 2–4 months were used (stock 000664, Jackson Laboratory). Mice were group housed with littermates until craniotomy surgery, after which they were singly housed. Mice were maintained on a 12–12-h (6 a.m.–6 p.m.) light–dark cycle. The holding room temperature was 21 ± 1 °C with a relative humidity of 30% to 70%. Irradiated rodent laboratory chow (LabDiet 5053) was provided ad libitum.

For two-photon experiments with WHaloCaMP1a_552_, male mice (C57BL/6J background) aged 2–4 months were used in this study (stock 000664, Jackson Laboratory). Mice were group housed with littermates in temperature- and humidity-controlled rooms with ad libitum access to water and rodent chow (PicoLab Rodent Diet 20, 5053 tablet, LabDiet–Land O’Lakes) on a 12-h light–dark cycle (9 p.m.–9 a.m., dark cycle and 9 a.m–9 p.m., light cycle).

### Retro-orbital injection of JF dye-ligands to mice

Retro-orbital injection of dye-HaloTag ligands was performed as previously described^10^ with minor modifications. The lyophilized dye-HaloTag ligand (200 nmol) was suspended in 20 µl DMSO. After vortexing, 20 µl of 20% Pluronic in DMSO was added, and the solution was pipetted up and down carefully to not form bubbles. PBS (80 µl; 137 mM NaCl, 10 mM phosphate, 2.7 mM KCl, pH 7.4) was carefully added to the solution and before letting the solution sit for 10 min to let foam and bubbles settle. This solution (100 µl) was injected into one eye of an anesthetized mouse using a 0.5-ml 27G syringe to the r.o. sinus.

### Two-photon imaging of WHaloCaMP1a_669_ in the mouse visual cortex

Adult C57/BL6 male mice (2–4 months old) were used. Four mice were prepared and imaged. Representative images from two mice are presented in the article. Viral injections (300 nl, 400 µm below the brain surface) were performed, followed by implantation of a 4-mm cranial window as described previously^66^, and r.o. injection of dye-ligands was performed 24 h before imaging.

Two-photon imaging of WHaloCaMP1a_669_ in the mouse visual cortex using long-wavelength light was performed on a customized adaptive optics two-photon microscope based on the Thorlabs Bergamo II microscope, previously described^19^. Mice were anesthetized with isoflurane using a precision vaporizer, 3% (vol/vol) in oxygen for induction and 0.5–2% (vol/vol) during visual stimulation. The mouse was placed in a custom-built goniometric heated holder to restrict movement and maintained a body temperature of 37 °C. Excitation was provided from a Coherent Discovery NX TPC. The objective was an Olympus XLUMPFLN objective, ×20, 1.0 NA with water immersion. Excitation was performed at 950 nm (25% of light at laser source) for EGFP and at 1,225 nm (100% of light at laser source) for WHaloCaMP1a_669_. Power ranged from 2 to 30 mW for 950 nm and from 5 to 15 mW for 1,225 nm. Emitted light was separated from excitation light via a 735-nm longpass dichroic and then filtered through a 525/50-nm bandpass dichroic (green PMT) and a 690/60-nm bandpass dichroic (near-infrared PMT). Light was collected via two Thorlabs PMT2100 PMTs and digitized via a laser-clocked National Instruments DAQ card controlled by ScanImage^65^ 2020 (Vidrio Technologies). Imaging parameters were 512 × 512 pixels with 44.8 frames per second at 2× zoom for a 200 × 200-µm field of view. Visual stimulation was displayed on an LCD screen placed 10 cm in front of the mouse’s eye, and moving bar stimuli were generated in MATLAB using Psychtoolbox^20^ (with 8 s on, 8 s off) in eight equally spaced directions spaced by equal time periods of mean luminance and were shown to the animal synchronized with the acquisition. Light from the visual stimulation screen was filtered so that it minimally impacted the red and green PMT channels. Functional images were analyzed in Suite2p^21^, and fluorescent traces were calculated with *F*_0_ as the lowest 0.5 percentile, calculating a rolling mean over 22 frames (0.5 s).

### Two-photon imaging of WHaloCaMP1a_552_ in the mouse visual cortex

Adult C57/BL6 male mice (2–4 months old) were used. The experiments were performed on four mice. A craniotomy 3.5 mm in diameter was first made over the left V1 of mice. Next, 40 nl of virus-containing solution (AAV2/1.CAMK2.NES-WHaloCaMP1a-EGFP, 9 × 10^12^ infectious units per ml) was injected 0.5 mm below the pia into the left V1 at four injection sites at the intersection points of the two left–right lines at bregma −3.4 mm and −4.0 mm and two anterior–posterior lines at 2.2 mm and 2.6 mm from the midline over 2 min. After the pipette was pulled out of the brain, a glass window made of a single coverslip (Fisher Scientific, no. 1.5) was embedded in the craniotomy and sealed in place with dental acrylic. A titanium headpost was then attached to the skull with cyanoacrylate glue and dental acrylic.

Visual stimuli were presented with a tablet monitor (Sceptre) displaying only blue light. The screen was positioned 15 cm from the right eye, covering 70° × 70° of visual space and oriented at ~40° to the long body axis of the animal. Visual stimuli were generated using custom-written codes based on Psychophysics Toolbox^67^ in MATLAB (Mathworks). During visual stimulation, the luminance level was kept constant. Each stimulus trial consisted of an 8-s blank period (uniform gray display at mean luminance), followed by 8 s of drifting grating (0.05 cycles per degree, temporal frequency of 1 Hz, eight randomized different directions). Each oriented drifting grating was presented for a total of five trials. Imaging was synchronized with ScanImage to start 4 s after the onset of the gray session for 8 s. Therefore, each imaging session covers 4 s of gray screen and 4 s of the drifting grating period.

Mice were kept on a warm blanket (37 °C) and anesthetized using 0.5% isoflurane and sedated with chlorprothixene (20–30 μl at 0.33 mg ml^−^^1^, intramuscular). Imaging was performed with a custom-built two-photon microscope with a resonant scanner. Imaging was performed 4 weeks after virus injection and 24-h after r.o. injection of the dye-ligand. Each experimental session lasted 45 min to 2 h. Multiple sections (imaging planes) may be imaged within the same mouse. Fluorophores were excited by a different wavelength with a femtosecond laser system (Chameleon Discovery, Coherent) that was focused by an Olympus ×25, 1.05-NA objective. Emitted fluorescence photons reflected off a dichroic longpass beam splitter (FF705-Di01–25x36, Semrock) and were split with a dichroic mirror (565DCXR, Chroma) and detected by PMTs H16201P-40//004, Hamamatsu) after filtering with a 510/84-nm filter (84-097, Edmund) for the green channel and two 750SP filters (64-332, Edmund) for the red channel. Images were acquired using ScanImage^65^ (Vidrio Technologies). Functional images (512 × 512 pixels, 268 × 268 μm^2^) of L2/L3 cells (50–300 μm under the pia mater) were collected at 15 Hz. For 850 nm, post-objective laser power was 15 mW for imaging 148–210 µm deep in the brain. For 1,050 nm, post-objective laser power between 92 and 140 mW was used depending on the depth (100–362 µm) of cells.

Imaging data were processed with custom programs written in MATLAB (Mathworks) and Fiji^60^. Images were registered with an iterative cross-correlation-based registration algorithm^68^. All identifiable cell bodies were outlined by hand as ROI. The averaged fluorescence signal within the ROI was calculated as the raw ROI signal, *F*_raw_. Neuropil subtraction was performed as follows. First, a square neuropil region, centered on an ROI, was determined by adding seven pixels to the width and height of the ROI, while excluding a two-pixel gap between the ROI and the neuropil region. Next, the average fluorescence signal of the neuropil excluding the ROI was calculated as the raw neuropil signal *F*_neuropil_. The neuropil signal was then baseline subtracted by subtracting the average of the lowest 5% values of the neuropil signal time trace to obtain Δ*F*_neuropil_. Neuropil subtracted fluorescence signal within the ROI was then calculated^69^ as *F* = *F*_raw_ − 0.7 × Δ*F*_neuropil_. We calculated Δ*F*/*F*_0_ = (*F* − *F*_0_)/*F*_0_, where *F* is the instantaneous fluorescence signal and *F*_0_ is the average fluorescence in the interval 4 s before the start of the visual stimulus. Visual responses were measured for each trial as Δ*F*/*F*_0_, averaged over the stimulus period. Visually responsive neurons were defined as cells with significant stimulus-related fluorescence changes (ANOVA across blank and eight direction periods, *P* < 0.01) with an average Δ*F*/*F*_0_ at preferred orientations greater than 10%. The fraction of cells detected as responsive was calculated as the number of significantly responsive cells over all the ROI analyzed. The cumulative distribution of peak Δ*F*/*F*_0_ responses included the maximal response amplitude from all analyzed cells, calculated as described above for each cell’s preferred stimulus. The orientation selectivity index (OSI) was calculated as described before^69,70^ by fitting the fluorescence response from individual cells to the eight drifting grating stimuli with two Gaussians, centered at the preferred response angle (*R*_pref_) and the opposite angle (*R*_opp_). The OSI was calculated as:

$${\rm{OSI}}=\frac{{R}_{{\rm{pref}}}-{R}_{{\rm{orth}}}}{{R}_{{\rm{pref}}}+{R}_{{\rm{orth}}}},$$

where *R*_orth_ is the angle orthogonal to the preferred angle.

Standard functions and custom-written scripts in MATLAB were used to perform all analyses. The data were tested for normal distribution. Parametric tests were used for normally distributed data, and nonparametric tests were applied to all other data. Bar graphs and mean ± s.e.m. were used to describe data with normal distribution, while box plots and median ± interquartile range (IQR) were used to describe non-normally distributed data. Box plots represent the median and 25th–75th percentiles, and their whiskers are shown in Tukey style (±1.5 × IQR). A nonparametric test (Wilcoxon signed-rank test) was used to examine paired data. Direct nonpaired comparisons between two groups were made using the Wilcoxon rank-sum test for non-normally distributed data. Statistical significance was defined as *P < 0.05, **P < 0.01, ***P < 0.001. Experiments were not performed blind. Sample sizes were not predetermined by statistical methods but were based on those commonly used in the field. Medians, IQR, means and s.e.m. are reported throughout the text.

### One-photon widefield imaging of WHaloCaMP1a_669_–EGFP versus iGECI–EGFP

Adult C57/BL6 male mice (2–4 months old) were used. Four mice were prepared, and three mice were imaged. Representative images from two mice are presented in the article. A craniotomy 3 mm in diameter was first made over the right-whisker somatosensory cortex (barrel cortex) of mice. Virus-containing solution (300 nl; AAV1.CAMK2.NES-WHaloCaMP1a-EGFP or AAV1.CAMK2.NES-iGECI-EGFP (10^12^ infectious units per ml)) was injected 0.3 mm below the dura at the following positions relative to the bregma: −1.5 mm anterior–posterior coordinates and 2.5 mm (AAV1.CAMK2.NES-WHaloCaMP1a-EGFP) or 4 mm (AAV1.CAMK2.NES-iGECI-EGFP) in the mediolateral coordinates. A glass window made of a single coverslip (Fisher Scientific, no. 1.5) was embedded in the craniotomy and sealed in place with dental acrylic. A titanium headpost was then attached to the skull with cyanoacrylate glue and dental acrylic. Mice were r.o. injected with 200 nmol JF_669_-HaloTag ligand 24 h before the imaging session. No exogenous biliverdin was supplied for imaging.

Mice were imaged on a custom-built upright microscope as previously described^62^ with modifications. For the excitation source, we used a four-wavelength LED (Thorlabs, LED4D067). To excite WHaloCaMP1a_669_ and iGECI, we used the 625-nm LED, and to image the EGFP expression marker, we used the LED at 470 nm. We used an imaging dichroic beam splitter with edge at 660 nm (FF660-Di02-25x36, Semrock) and a 671-nm emission filter (LP02-671RU-25, Semrock) to clean up fluorescence signal before camera detection. For green fluorescence imaging, we used a Thorlabs GFP filter set (MDF-GFP). Fluorescence signals were imaged using an InGaAs camera (Ninox 640 II, Raptor Photonics). Images were acquired using µManager, an open-source microscopy software^63^. To obtain an overview image of the cranial window, we used a ×4 objective (Nikon ×4 Plan Fluor, 0.13 NA and working distance of 17.2 mm). For depth experiments and functional imaging, we used a ×10 objective (Nikon ×10 Plan Fluor, 0.3 NA and working distance of 3.5 mm). For the functional whisker-stimulation experiments, images were acquired at the focal plane. Images were acquired in a 0.11-s period for 1,727 frames. We conducted the whisker air puff stimulation using a Picospritzer III system (Parker Hannifin) with 30 psi of air pressure and a duration of 50 ms.

Stimulation was controlled using the MATLAB-based waveform generator WaveSurfer to elicit a pulse every 30 s. The pulse was delivered in a square pulse train for six consecutive trials, with a 10-s delay for the first trial.

For whisker-stimulation experiments, no ROI was chosen, and we used the whole field of view for analysis. We calculated Δ*F*/*F*_0_ = (*F* − *F*_0_)/*F*_0_, where *F* is the instantaneous fluorescence signal and *F*_0_ is the average fluorescence in the 6-s interval before the onset of the air puff stimulus.

### Zebrafish larvae

All zebrafish experiments were conducted in accordance with animal research guidelines from the National Institutes of Health and were approved by the Institutional Animal Care and Use Committee and the Institutional Biosafety Committee of Janelia Research Campus. Larvae were reared using standard protocols in 14:10 light–dark cycles at 28.5 °C.

To generate WHaloCaMP1a lines, NES-WHaloCaMP1a or NES-WHaloCaMP1a-EGFP were cloned into Tol2 vectors with the *elavl3* pan-neuronal promoter. The plasmid was injected into one to two cell embryos from *casper* background zebrafish with mRNA encoding the Tol*2* transposon. Potential founders were selected either by screening with the JF_522_-HaloTag ligand or based on EGFP expression. Potential founders were then screened by outcrossing to *casper* and selecting progeny (F_1_ generation) that expressed WHaloCaMP1a in the central nervous system. Experiments were performed on larvae at 4–5 dpf produced from crosses with *casper*, from in-crosses or from crossing with other lines for multicolor experiments. For three-color multiplexed functional imaging, we crossed Tg(*elavl3*:NES-WHaloCaMp1a) with Tg(*actb2*:iGlucoSnFR; *acta1a*:jRGECO1a)^29^ animals; for neuronal and astrocyte imaging, we crossed Tg(*elavl3*:NES-WHaloCaMp1a-EGFP) with *Tg*(*gfap:jRGECO1b*)^30^ animals. For comparing WHaloCaMP1a_669_ to jRGECO1b in the same neuronal population, we crossed Tg(*elavl3*:NES-WHaloCaMP1a-EGFP) with Tg(*elavl3*:jRGECO1b)^28^ animals.

### Dye delivery to zebrafish larvae

Dye-ligands were delivered to the central nervous system of zebrafish larvae by adding the dye-HaloTag ligand (final concentration of 4 µM from a 2-mM stock in DMSO) to the system water for 2 h. The larvae were then washed 2× for 1 h with fresh system water before mounting for whole-brain imaging.

### Whole-brain light-sheet imaging with SiMView

Whole-brain imaging was performed on a SiMView microscope as previously described^71,72^. Tg(*elavl3*:NES-WHaloCaMP1a-EGFP) zebrafish larvae at 5 dpf were labeled with the JF_669_-HaloTag ligand, as described above. The fish were screened for EGFP fluorescence, and EGFP-positive fish were paralyzed in a drop of 1 mg ml^−1^ α-bungarotoxin solution (Invitrogen) in system water for 30 s before recovering in system water for 30 min. Zebrafish were placed in 2% agarose in system water and positioned in a custom-designed glass capillary (outer diameter of 2 mm, 20 mm long; Hilgenberg), which was mounted in an imaging chamber filled with system water. Fluorescence light-sheet imaging of WHaloCaMP1a_669_ was performed with Nikon ×16, 0.8-NA water-dipping objectives and a Hamamatsu ORCA-Fusion BT camera. A 685-nm laser (1.6 mW cm^−2^ at the sample) was used to excite WHaloCaMP1a_669_, and light was collected through a 795/188-nm detection filter (Semrock). Volumetric imaging was performed with an exposure time of 5 ms at a step size of 4 μm across a 160-μm-deep volume, resulting in a volume imaging rate of four image stacks per second. Image analysis was performed as previously described^71^, using a custom-written pipeline in MATLAB (Mathworks). After analysis, WHaloCaMP1a_669_ activity was visualized by creating a multichannel composite image of the ∆*F*/*F* time series images, which were superimposed on a static anatomical reference image. The anatomical reference image was generated by computing a projection of the ∆*F*/*F* images along the *z* and time axes and applying a CLAHE filter with a block size of 15, 255 bins and a maximum gradient of 4.

### Multiplexed light-sheet imaging of far-red, red and green fluorescence in zebrafish larvae

Multiplexed light-sheet single-plane functional imaging was performed on a Zeiss Lightsheet Z.1 microscope with two pco.edge 5.5 sCMOS cameras. For single-cell resolution imaging of neuronal and astrocyte activity, fish were paralyzed in a drop of 1 mg ml^−1^ α-bungarotoxin solution (Invitrogen) in system water for 30 s before recovering in system water for 5 min. This paralysis was not performed for three-color multiplexed imaging of Tg(*elavl3*:NES-WHaloCaMP1a) × Tg(*actb2*:iGlucoSnFR; *acta1a*:jRGECO1a) animals to retain the muscle Ca^2+^ transients. Fish were mounted in 2% low melting agarose in system water in a glass capillary, and the solidified agarose extruded into an imaging chamber filled with system water for imaging. For imaging with 4-AP, a solution of 8 mM was prepared in system water, which was diluted into the imaging chamber before imaging. The final concentration of 4-AP was 500–700 nM.

Images were acquired using an Objective W Plan-Apochromat ×10, 0.5 M27 75-mm illumination objective and two ×5, 0.1-NA objectives (with a correction ring) for side illumination. As we saw artifacts on dual-side illumination, only one side of illumination was used during acquisition. Pivot scan was always on during acquisition. Before each imaging session, the cameras were manually aligned and the light sheet was corrected using the correction rings on the ×5 illumination objectives. Single-plane light-sheet imaging was performed with 488 nm, and 561-nm and 638-nm excitation laser lines and a laser-blocking filter (Filter Module LBF 405/488/561/638) were used in the excitation light path. Exposure time was 234.69 ms.

For three-color functional imaging of Tg(*elavl3*:NES-WHaloCaMP1a) × Tg(*actb2*:iGlucoSnFR; *acta1a*:jRGECO1a) animals, two tracks were used with a filter change between the track acquisitions. This filter change decreased the imaging to 5 s per frame. In track 1, 488-nm and 638-nm excitation was used with a 560-nm beam splitter. The emission filter (505–545 nm) was in front of camera 1, and a longpass 660-nm filter was in front of camera 2. In track 2, 561-nm excitation light was used with a 640-nm beam splitter and a 575–615-nm emission filter in front of camera 1. Images were acquired at 1× zoom for up to 60 min at 1,000 × 1,800 pixels. Images where registered using Suite2p^31^ before ∆*F*/*F*_0_ values of ROI determined in Fiji^60^ were calculated using a rolling average of 250 frames with the lowest 0.1 quantile used for *F*_0_.

For dual-color imaging with Tg(*elavl3*:NES-WHaloCaMP1a-EGFP) × *Tg*(*gfap:jRGECO1b*) and Tg(*elavl3*:NES-WHaloCaMP1a-EGFP) × *Tg*(*elavl3:jRGECO1b*) animals, anatomical images were first acquired, with single-fluorophore excitation of EGFP, 488-nm excitation, 560-nm beam splitter, 505–545-nm emission filter; jRGECO1b, 561-nm excitation, 640-nm beam splitter, 575–615-nm emission filter; WHaloCaMP1a_669_, 638-nm excitation, 560-nm beam splitter, longpass 660-nm emission filter. Functional imaging was performed with simultaneous 561-nm and 638-nm excitation light, a 560-nm beam splitter and a 575–615-nm emission filter in front of camera 1 and a longpass 660-nm emission filter in front of camera 2. The 561-nm light was reduced to the lowest amount to limit bleedthrough of the jRGECO1b signal to the far-red emission channel. Images were acquired at 2.5× zoom, a pixel size of 1,960 × 1,960, 354.4 µm × 354.4 µm and 0.18 µm per pixel. Time series of 1,500 frames were collected. Time series were concatenated together when longer analysis was desired. Images were downscaled for analysis.

### Image analysis of single-plane light-sheet neuronal and astrocyte imaging

For analysis of dual-color functional imaging of WHaloCaMP1a_669_ in neurons and jRGECO1b in astrocytes, a combination of Fiji^60^, Suite2p^31^, Cellpose^32^ and Rastermap^33^ was used. The two channels were analyzed separately. First, Suite2p was used for registration of the images, and cell segmentation was performed using Cellpose seeding with a manually determined diameter. Suite2p was then used to extract traces from the segmented cells. The ∆*F*/*F*_0_ was calculated using a rolling average of 100 frames (for *elavl3*-jRGECO1b acquisitions) or 200 frames (for *gfap*-jRGECO1b acquisitions), with *F*_0_ calculated from the lowest 0.1 quantile. Fluorescent traces were clustered with Rastermap before being presented as heatmaps.

### Fluorescence lifetime imaging microscopy

FLIM was performed on a STELLARIS 8 FALCON microscope (Leica Microsystems) with a white light laser with tunable excitation wavelengths from 440 to 790 nm operating at 80 MHz. For full lifetime decay calculations, the microscope was set to operate at 40 MHz. A ×20 water-immersion objective (Leica, HC PL IRAPO, NA = 0.75) or a ×25 water-immersion objective (Leica, HC FLUOTAR L, NA = 0.95) was used. HyD photon-counting detectors were used.

### FLIM of purified protein

To generate a calibration curve of lifetime versus Ca^2+^ concentration in purified protein, WHaloCaMP1a was labeled with a dye-ligand and diluted into solutions made by mixing different proportions of concentrations made from Calcium Calibration Buffer Kit #1 (Invitrogen). A small drop was placed on a microscope slide, and a double adhesive divider was placed on the slide before it was covered with a coverslip (#1.5). For WHaloCaMP1a_494_, excitation was 488 nm, and emission collected on a HyDX2 was set to 500–575 nm. For WHaloCaMP_552_, excitation was 561 nm, and emission collected on a HyDS3 was set to 575–650 nm. For WHaloCaMP_669_, excitation was 671 nm, and emission collected on a HyDX4 was set to 680–780 nm. For WHaloCaMP_722_, excitation was 730 nm, and emission collected on a HyDR5 was set to 745–850 nm. For iGECI imaging, excitation was 638 nm, and emission collected on a HyDX4 was set to 650–700 nm.

### FLIM in tissue cultured cells

The Janelia Cell Culture Facility performed tissue culture and regular *Mycoplasma* testing. HeLa cells (CCL-2) were purchased from ATCC. No authentication of the cell line was performed. Cells were cultured in DMEM medium (phenol red free, Life Technologies) supplemented with 10% (vol/vol) FBS (Life Technologies) and 1 mM GlutaMAX (Life Technologies). Cells were cultured at 37 °C in a humidified 5% (vol/vol) CO_2_ environment. Cells were transiently transfected by nucleofection (Lonza) 24 h before imaging and plated in 35-mm dishes (MatTek, #1.5 coverslip). Before imaging, cells were labeled with 50 nM JF_669_-HaloTag ligand in growth medium before being washed 2× with imaging buffer containing 145 mM NaCl, 2.5 mM KCl, 10 mM glucose, 10 mM HEPES, pH 7.4, 2 mM CaCl_2_ and 1 mM MgCl_2_ for histamine stimulation experiments or Hanks’ Balanced Salt Solution with no Ca^2+^, no magnesium and no phenol red (Thermo Fisher Scientific, 14175095) for Ca^2+^ titration experiments. Images were collected at 1,600 × 1,600 pixels, with the pinhole set to 1 AU and 2× zoom. Images were collected every 5 s for 10 min.

For cellular Ca^2+^ titration experiments, Ca^2+^-free Hanks’ Balanced Salt Solution was replaced with solutions with Ca^2+^_free_ concentrations made from Calcium Calibration Buffer Kit #1 (Invitrogen) with 0 mM and 10 mM CaEGTA and equilibrated for 10 min at 37 °C. Digitonin (Promega, G944A) was then used to permeabilize the cells. The digitonin stock was diluted to 2 mg ml^−^^1^ in buffer, and 1 µl was added to the 2-ml volume in the imaging dish immediately before acquisition, giving a final concentration of 1 µM.

For histamine stimulation experiments, histamine was diluted in imaging buffer to a 100-mM stock solution. This was further diluted by adding 20 µl to the 2 ml of imaging buffer on the cells to give a final concentration of 1 µM.

### FLIM of neurons in zebrafish larvae

Zebrafish larvae (Tg(*elavl3*:NES-WHaloCaMP1a-EGFP, 4–5 dpf) were labeled and paralyzed as described above. The fish were then mounted, with the brain closest to the coverslip, in 2% low melting agarose in a 35-mm dish (MatTek, #1.5 coverslip). The fish were imaged in system water with 6× zoom, the pinhole set to 1 AU and 512 × 512 pixels. The imaging rate was one frame per second.

### FLIM image analysis

Fluorescence lifetime fitting was performed in LAS X FLIM software. A spatial binning of 2 was performed, and each time point was split in the software. A three-component fit was used to fit the fluorescence lifetime. The amplitude-weighted fluorescence lifetime for the whole image is reported for WHaloCaMP1a labeled with different dye-ligands and imaged at 40 MHz. For quantifying concentrations of Ca^2+^ in HeLa cells and neurons of zebrafish larvae, each pixel in the image was fit to a decay curve. The fit was performed on images that had a threshold of 20–50 counts for each pixel. The image was then exported to a TIFF file with a scaling factor of 0.001 on the lifetime and analyzed in Fiji^60^. A calibration curve from the purified protein or digitonin-lysed cells was fit using a four-parameter dose–response curve (variable slope) using GraphPad Prism software. a is the value of fluorescence at the bottom of the curve, *b* is the value of fluorescence at the top of the curve, EC_50_ is the concentration of agonist that gives a response halfway between the bottom and the top, and *h* is the Hill or cooperative coefficient.

$$\begin{array}{cc}y=a+\frac{{x}^{h}\times (b-a)}{{x}^{h}+\,{{{\rm{EC}}}_{50}}^{h}} & x={{\rm{EC}}}_{50}{\left(\frac{a-y}{y-b}\right)}^{\left(\frac{1}{h}\right)}\end{array}$$

To calculate the cellular Ca^2+^ concentration, the equation was rearranged, and a value of *x* = [Ca^2+^] was calculated for each measured value of *y* (lifetime (ns)) for an ROI in a time series. The intensity channel was also exported from LAS X FLIM software, and the ∆*F*/*F* was calculated from the first quartile for the time series. For visualization purposes for the lifetime time series, a Gaussian filter of 0.8 was used. All quantitative analysis was performed on the raw exported images before any image processing.

### Reporting summary

Further information on research design is available in the Nature Portfolio Reporting Summary linked to this article.

## Online content

Any methods, additional references, Nature Portfolio reporting summaries, source data, extended data, supplementary information, acknowledgements, peer review information; details of author contributions and competing interests; and statements of data and code availability are available at 10.1038/s41592-024-02411-6.

## Supplementary information

Supplementary Information Supplementary Figs. 1–24, Tables 1–5 and Notes 1 and 2

Reporting Summary

Supplementary Video 1 Volumetric imaging in the near-infrared of neuronal activity in Tg(*elavl3*:NES-WHaloCaMP1a-EGFP) animals labeled with JF_669_-HaloTag ligand 5 dpf. The zebrafish larva was paralyzed with α-bungarotoxin. Imaged at 4 volumes per second. Displayed as ∆*F*/*F*_0_ (red-hot color look-up table) superimposed on anatomy (gray). Scale bar, 100 µm. Time stamp in min:s.

Supplementary Video 2 Single-plane light-sheet imaging of Tg(*elavl3*:NES-WHaloCaMP1a, *actb2*:iGlucoSnFR; *acta1a*:jRGECO1a) animals labeled with JF_669_-HaloTag ligand at 5 dpf. Imaged at 0.2 Hz. Three colors are displayed in raw fluorescence together with an overlay of fluorescence of all three channels.

Supplementary Video 3 Single-plane light-sheet imaging of Tg(*elavl3*:NES-WHaloCaMP1a-EGFP, *gfap*:jRGECO1b) animals labeled with JF_669_-HaloTag at 5 dpf. The zebrafish larva was paralyzed with α-bungarotoxin and imaged at 4 Hz. Each channel is displayed as raw fluorescence as grayscale, with the insert showing the overlay of WHaloCaMP1a_669_ in magenta and jRGECO1b in yellow. Time stamp in min:s.

Supplementary Video 4 Single-plane light-sheet imaging of Tg(*elavl3*:NES-WHaloCaMP1a-EGFP, *gfap*:jRGECO1b) animals labeled with JF_669_-HaloTag at 5 dpf and treated with 4-AP. The zebrafish larva was paralyzed with α-bungarotoxin. Imaged at 4 Hz. Each channel is displayed as raw fluorescence as grayscale, with the insert showing the overlay of WHaloCaMP1a_669_ in magenta and jRGECO1b in yellow. Three time series of 6.2 min are concatenated. Time stamp in min:s.

Supplementary Video 5 Raw fluorescence intensity (left) and FLIM (right) of WHaloCaMP1a labeled with JF_669_-HaloTag ligand in HeLa cells stimulated with histamine (1 µM). Lifetime color bar is scaled ×1,000 ns. One frame was collected every 5 s. Time stamp in min:s. Still images and ∆*F*/*F*_0_ or [Ca^2+^]_calc_ from a calibration curve of lifetime in Fig. 5d.

Supplementary Video 6 Overlaid near-infrared intensity and lifetime using LAS X Leica FLIM software of Tg(*elavl3*:NES-WHaloCaMP1a-EGFP) animals labeled with JF_669_-HaloTag ligand. The zebrafish larva was paralyzed with α-bungarotoxin and imaged at 1 Hz. Time stamp in min:s. Still images and ∆*F*/*F*_0_ or [Ca^2+^]_calc_ from a calibration curve of lifetime in Fig. 5f.

## Data Availability

The data used to generate the figures in this study are available from the figshare repository: 10.25378/janelia.25934782 (ref. ^73^). The structure of HaloTag7 bound to JF_669_ has been deposited in the Protein Data Bank (PDB 8SW8). Sequences have been deposited to GenBank under accession codes PQ141081 (WHaloCaMP1a), PQ141082 (WHaloCaMP1b) and PQ141083 (WHaloCaMP_eNOSpep). DNA plasmids encoding WHaloCaMP used in this work are available from Addgene as follows: 205303, pRSET-WHaloCaMP1a-EGFP; 205304, pRSET-WHaloCaMP1a; 205305, pRSET-WHaloCaMP1b-EGFP; 205306, pRSET-WHaloCaMP-eNOSpep-EGFP; 205307, pAAV-synapsin-WHaloCaMP1a-EGFP; 205308, pAAV-synapsin-WHaloCaMP1a; 205309, pAAV-CaMKII-WHaloCaMP1a-EGFP; 205310, pAAV-CaMKII-WHaloCaMP1a; 205311, pTol2-elavl3-WHaloCaMP1a-EGFP; 205312, pTol2-elavl3-WHaloCaMP1a; 205313, pCAG-WHaloCaMP1a-EGFP; 205314, p10xUAS-WHaloCaMP1a-EGFP.

## Code availability

The MATLAB programs for ScanImage for data acquisition and analysis for multiplexed imaging in acute brain slices with WHaloCaMP1a_669_ and FLIM-AKAR are available at https://github.com/YaoChenLabWashU/2pFLIM_acquisition. MATLAB 2012b was used.
