# Supplementary material for: A modular chemigenetic calcium indicator for multiplexed in vivo functional imaging
Source: Nat Methods. 2024 Sep 20;21(10):1916–25. doi: 10.1038/s41592-024-02411-6 (PMC11466818; doi:10.1038/s41592-024-02411-6)
Supplement: Supplementary file 1 — Supplementary Figs. 1–24, Tables 1–5 and Notes 1 and 2 [file 41592_2024_2411_MOESM1_ESM.pdf]

nature methods

Supplementary information https://doi.org/10.1038/s41592-024-02411-6

A modular chemigenetic calcium indicator for multiplexed in vivo functional imaging

In the format provided by the authors and unedited

Table of Contents

Supplementary Figure 1. Fluorescence emission of JF_{669}-HaloTag ligand does not change with addition of Ca^{2+} when used with HaloCaMPs. 3

Supplementary Figure 2. Kinetics of Ca^{2+} unbinding from WHaloCaMP1a bound to JF_{669}-HaloTag ligand. 4

Supplementary Figure 3. One-photon bleaching during widefield microscopy of $WHaloCaMP1a_{669}$. 5

Supplementary Figure 4. In-gel fluorescence and total protein stain of WHaloCaMP1a labeled with different dyes. 6

Supplementary Figure 5. Two-photon cross section of WHaloCaMP1a. 7

Supplementary Figure 6. pH stability of WHaloCaMP1a bound to different dyes. 8

Supplementary Figure 7. Individual traces of fluorescence response of WHaloCaMP1a or biliverdin-binding protein-based calcium indicator iGECI in a primary neuron culture field stimulation 9

Supplementary Figure 8. Summary of properties of WHaloCaMP1a bound to different dye-ligands in a field stimulation assay in cultured neurons. 10

Supplementary Figure 9. $WHaloCaMP1a_{669}$ is spectrally compatible with the channelrhodopsin variant CheRiff. 11

Supplementary Figure 10. Multiplexed imaging in acute brain slices with $WHaloCaMP1a_{669}$ and FLIM-AKAR. 12

Supplementary Figure 11. Individual traces for $WHaloCaMP1a_{669}$ imaging of odor responses in flies. 13

Supplementary Figure 12. Odor responses in fruit flies recorded with different WHaloCaMP variants and JF dye-ligands. 14

Supplementary Figure 13. Two-photon imaging of WHaloCaMP1a labeled with JF_{669}-HaloTag ligand in mouse visual cortex. 15

Supplementary Figure 14. Two-photon imaging of $WHaloCaMP1a_{552}$ in mouse primary visual cortex (V1). 16

Supplementary Figure 15. Single trial and individual traces of two-photon imaging of $WHaloCaMP1a_{552}$ in mouse primary visual cortex (V1). 17

Supplementary Figure 16. Functional imaging in the mouse V1 of $WHaloCaMP1a_{552}$ and quantitative analysis. 18

Supplementary Figure 17. One-photon imaging of $WHaloCaMP1a_{669}$-EGFP and iGECI-EGFP in mouse cortex 19

Supplementary Figure 18. Comparison of WHaloCaMP1a$_{669}$ and jRGECO1b during light sheet imaging of neurons in zebrafish larvae. **........ 20**

Supplementary Figure 19. Three-color functional multiplexed imaging in zebrafish larvae with WHaloCaMP1a$_{669}$, jRGECO1a and iGlucoSnFR. **........ 21**

Supplementary Figure 20. Dual-color functional imaging of astrocyte and neuronal Ca^{2+} in zebrafish larvae during spontaneous activity or in the presence of 4-aminopyridine. **........ 22**

Supplementary Figure 21. Dual-color functional imaging of astrocyte and neuronal Ca^{2+} in zebrafish larvae over longer time in the absence of 4-aminopyridine. **........ 23**

Supplementary Figure 22. Calibration of WHaloCaMP1a$_{669}$ for quantitative [Ca^{2+}] determination by FLIM. **........ 24**

Supplementary Figure 23. WHaloCaMP1a$_{669}$ as a FLIM probe in HeLa cells. **........ 25**

Supplementary Figure 24. In vivo quantitative FLIM for [Ca^{2+}] in zebrafish larvae. **........ 26**

Supplementary Table 1. X-ray crystallography structural and refinement statistics **........ 27**

Supplementary Table 2. Comparison of the photophysical properties of WHaloCaMP1a$_{669}$ and biliverdin-binding protein-based calcium indicators. **........ 28**

Supplementary Table 3. Fluorescence response of WHaloCaMP1a or biliverdin-binding protein-based calcium indicators in a primary neuron culture field stimulation assay **........ 29**

Supplementary Table 4. Decay properties calculated for WHaloCaMP1a bound to different dyes in a field stimulation assay in cultured neurons. **........ 30**

Supplementary Table 5. Representative Illumination imaging parameters for WHaloCaMP1a **........ 31**

Supplementary sequences of WHaloCaMPs **........ 32**

Supplementary Note 1 **........ 33**

Supplementary Note 2 **........ 37**

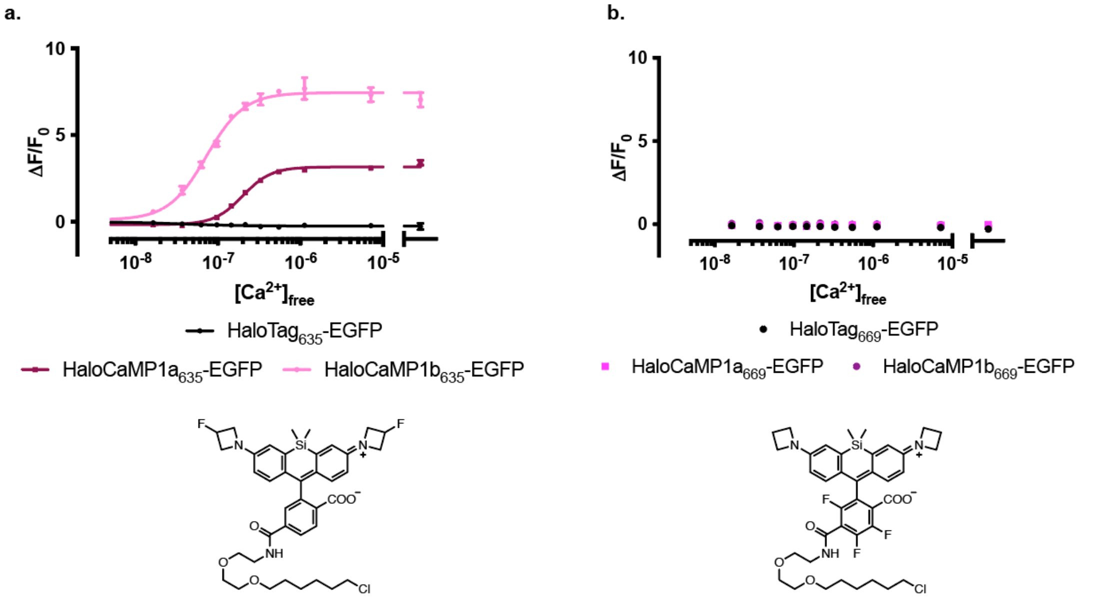

Supplementary Figure 1. Fluorescence emission of JF_{669}-HaloTag ligand does not change with addition of Ca^{2+} when used with HaloCaMPs.

a, Ca^{2+} titrations with HaloTag7[1]-EGFP, HaloCaMP1a and HaloCaMP1b[2] labeled with JF_{635}-HaloTag ligand. **b**, Ca^{2+} titrations of same protein variants as in (**a.**) but labeled with JF_{669}-HaloTag ligand. $HaloCaMP1a_{635}$ and $HaloCaMP1b_{635}$ are both calcium indicators, while $HaloCaMP1a_{669}$ and $HaloCaMP1b_{669}$ are not. The titrations are plotted with the mean and s.d. of two technical replicates. Each titration is a representative example of titrations performed at least three times with newly purified protein.

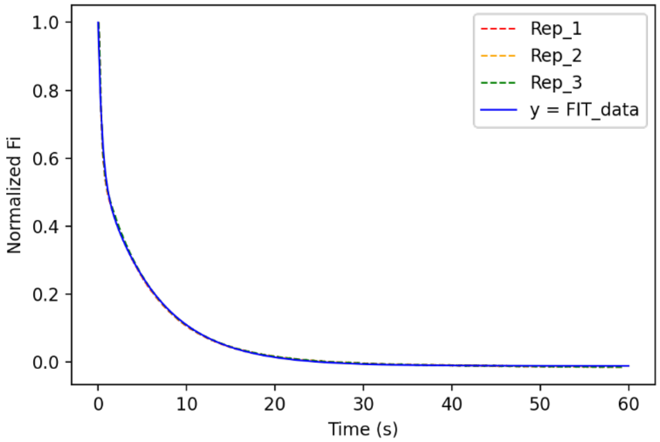

	$WHaloCaMP1_{a669}$
Model	$y = a_0 + a_1(1 - e^{-b_1 \times t}) + a_2(1 - e^{-b_2 \times t})$
fit	$y = 1 + (-0.57)(1 - e^{-0.16 \times t}) + (-0.43)(1 - e^{-2.3 \times t})$<br>$a_0$ was constrained to 1.0.
k_{off} (s^{-1})	$Dissociative_1$: 0.16<br>$Dissociative_2$: 2.3

Supplementary Figure 2. Kinetics of Ca^{2+} unbinding from WHaloCaMP1a bound to JF_{669}-HaloTag ligand.

A stopped flow instrument was used to follow the decrease in fluorescence emission from recombinant calcium saturated $WHaloCaMP1a_{669}$ following rapid mixing with excess calcium chelator (EGTA, 10 mM). Fluorescence decay was fit to a two-phase exponential model. Three technical replicates (Rep1, Rep 2, Rep 3) was performed, normalized to the initial fluorescence intensity at time 0.

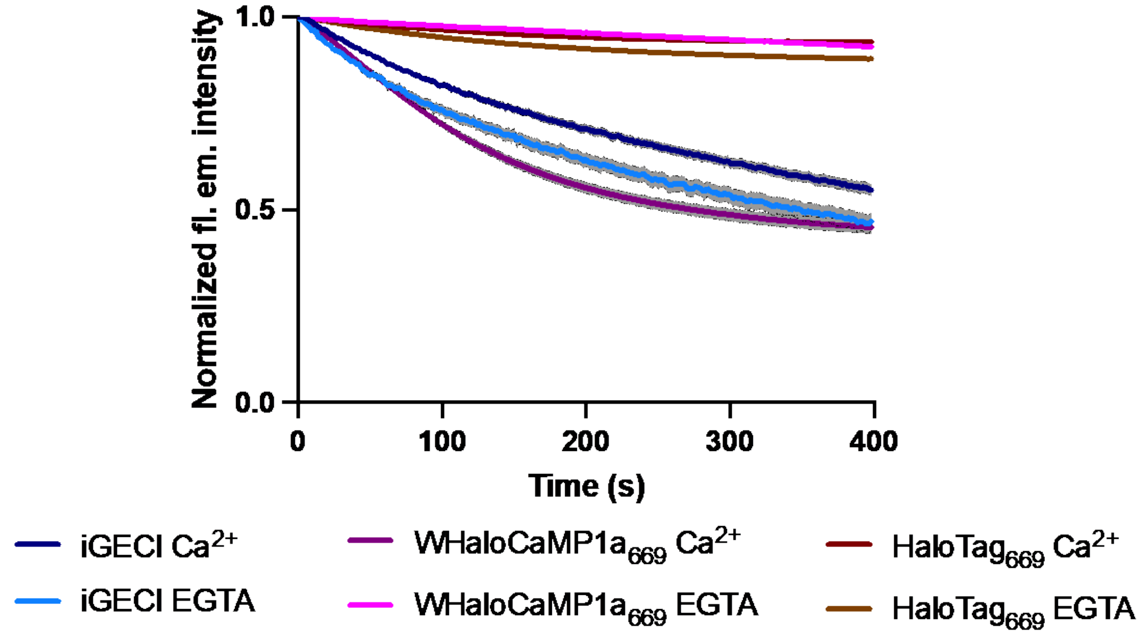

Protein*	Halftime (s)	95% confidence interval halftime (s)
**iGECI, Ca^{2+}**	204	190 to221
**iGECI, EGTA**	160	148 to 176
**WhaloCaMP1a$_{669}$, Ca^{2+}**	98	95 to 102

*one-photon bleaching halftimes of HaloTag$_{669}$ in Ca^{2+}, HaloTag$_{669}$ in EGTA, and WHaloCaMP1a$_{669}$ in EGTA could not be fit under these experimental conditions. One-photon bleaching halftimes of these proteins are longer than 400 s under these illumination conditions.

Supplementary Figure 3. One-photon bleaching during widefield microscopy of WHaloCaMP1a$_{669}$.

Aqueous droplets of purified protein in octanol were used to determine bleaching rates on an inverted widefield microscope. The droplets were continuously illuminated at 23 mW/mm^2 for 400 seconds, imaging at 0.5 Hz. Approximately 20 droplets were imaged in each bleaching trial and three bleaching trials were pooled to make the bleaching curve. Each fluorescence trace was normalized to the fluorescence emission in the first frame. The bleaching curves were fit with a one phase decay in GraphPad Prism software.

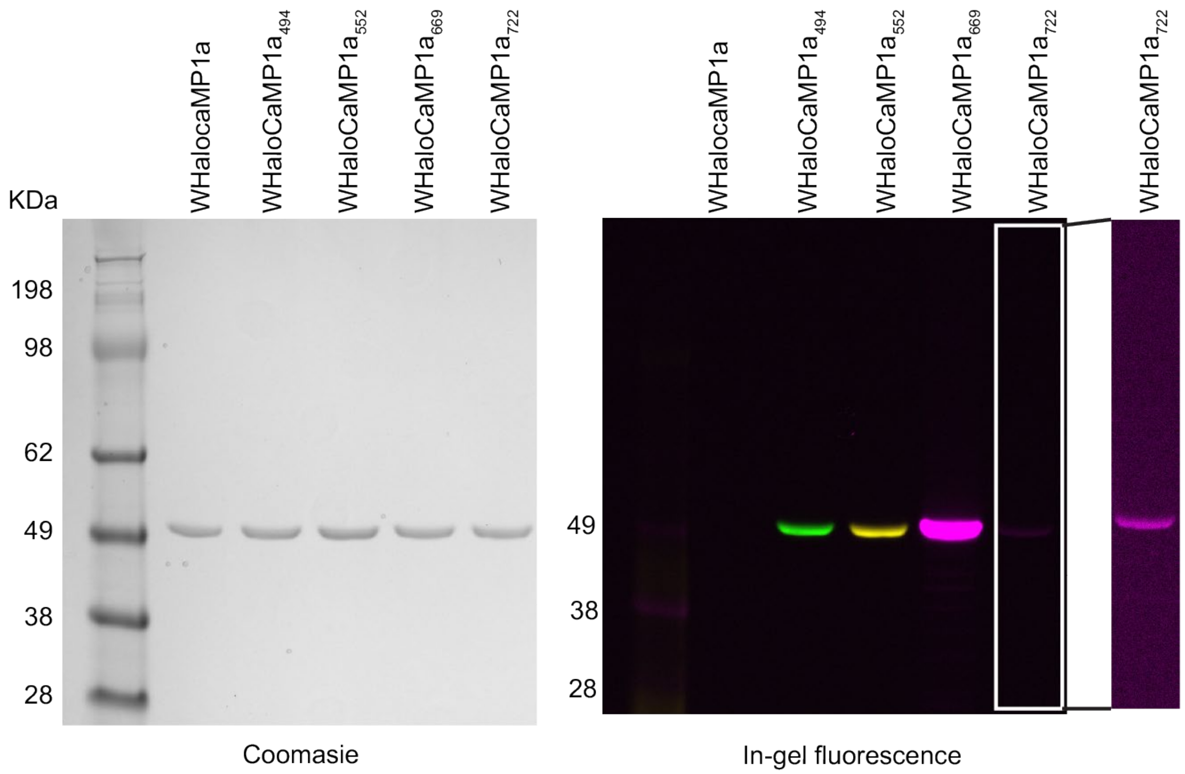

Supplementary Figure 4. In-gel fluorescence and total protein stain of WHaloCaMP1a labeled with different dyes.

Denaturing sodium dodecyl-sulfate polyacrylamide gel electrophoresis (SDS-PAGE) was used to separate purified WHaloCaMP1a labeled with different dye-ligands and LEDs with corresponding filters on a gel imager were used to image the in-gel fluorescence. WHaloCaMP1a$_{722}$ could be imaged with far-red filters if the brightness and contrast was increased in comparison to WHaloCaMP1a$_{669}$, as seen in the white box to the far right. This is a representative gel from in gel-fluorescence experiments that have been repeated more than three times with similar results.

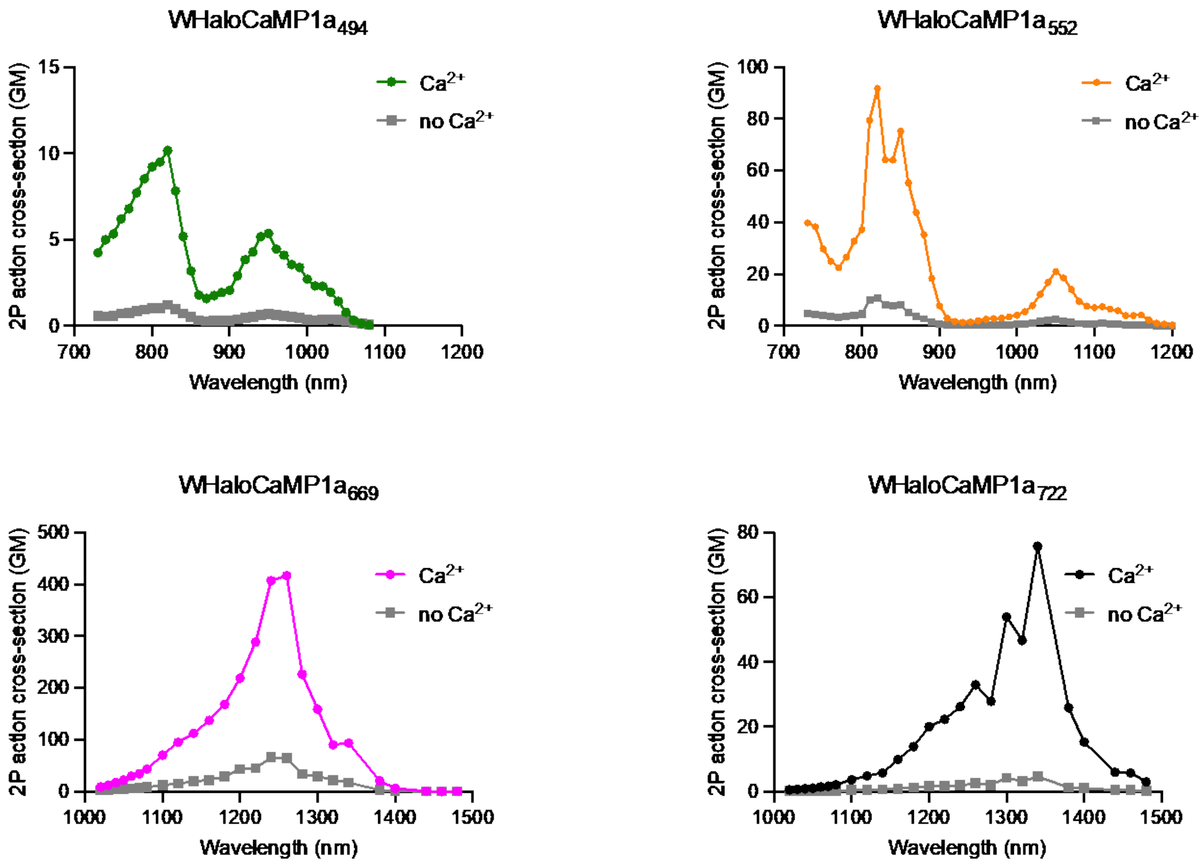

Supplementary Figure 5. Two-photon cross section of WHaloCaMP1a.

WHaloCaMP1a bound to JF_{494}-HaloTag ligand, JF_{552}-HaloTag ligand, JF_{669}-HaloTag ligand and JF_{722}-HaloTag ligand. 2P action cross section spectra were collected in purified protein prelabeled with dyes. Ca^{2+} is 39 µM free Ca^{2+} and no Ca^{2+} is 10 mM EGTA.

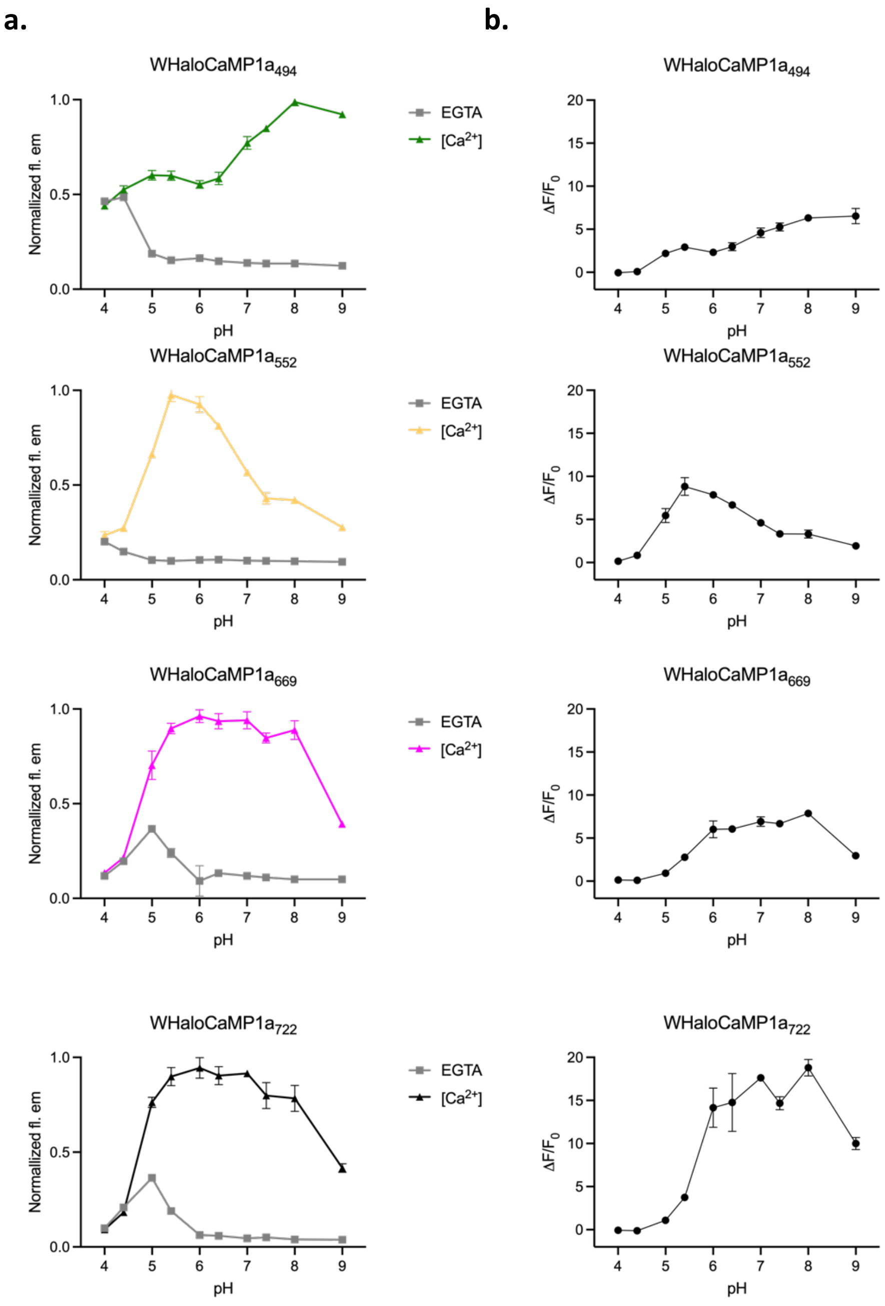

Supplementary Figure 6. pH stability of WHaloCaMP1a bound to different dyes.

a, Flourescence emission of WHaloCaMP1a bound to JF$_{494}$-HaloTag ligand, JF$_{552}$-HaloTag ligand, JF$_{669}$-HaloTag ligand and JF$_{722}$-HaloTag ligand in different pH values. Flourescence emission is normalized to the maximum flourescence emission. **b**, Calculated $\Delta F/F_0$ of WHaloCaMP1a bound to dyes at different pH. Each value is plotted as the mean and s.d. of three technical replicates.

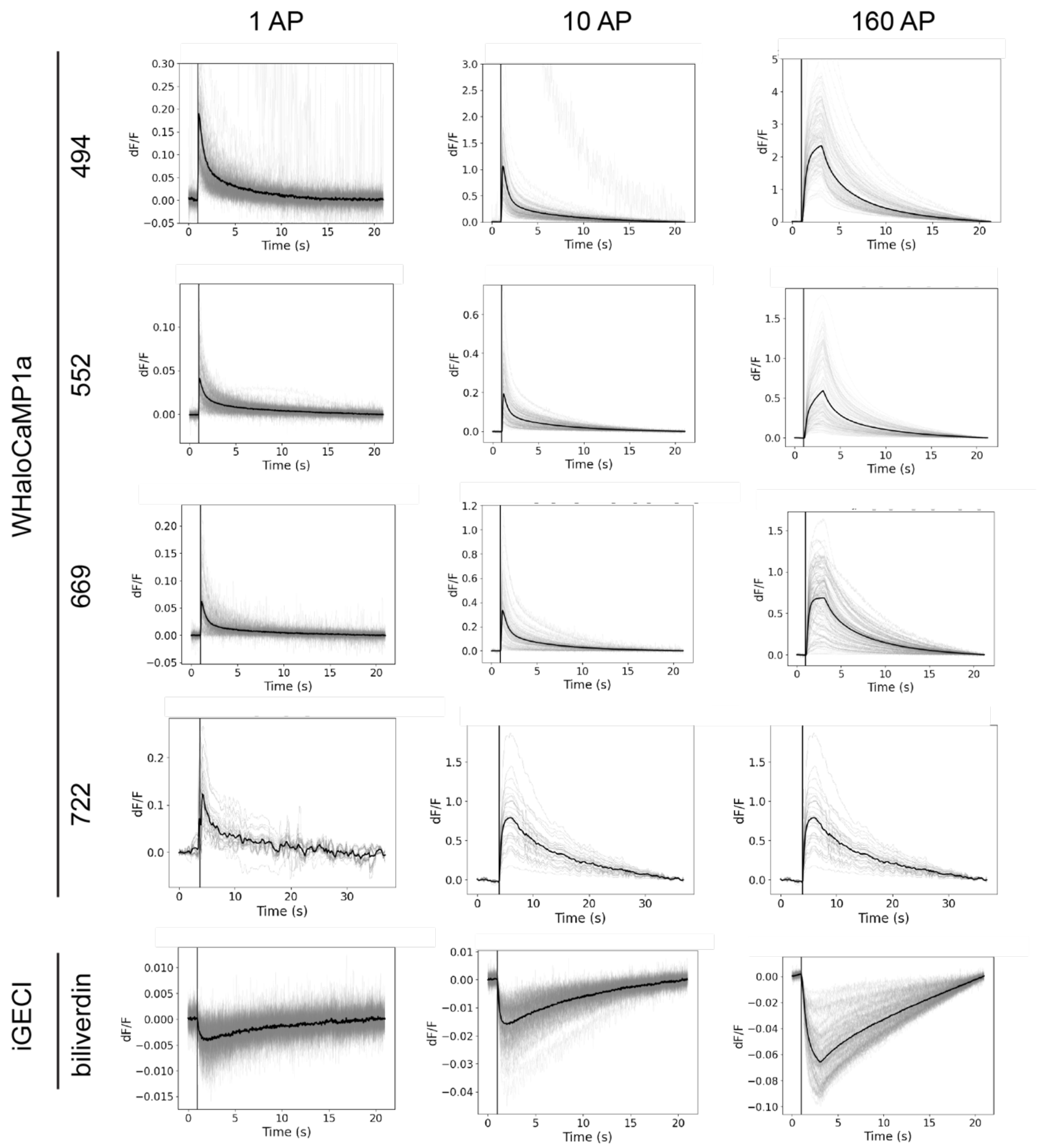

Supplementary Figure 7. Individual traces of fluorescence response of WHaloCaMP1a or biliverdin-binding protein-based calcium indicator iGECI in a primary neuron culture field stimulation

Individual traces (grey) and mean (black) for WHaloCaMP1a bound to different dyes-HaloTag ligands (HTL) and the brightest biliverdin-binding calcium indicator, iGECI. Stimulus is indicated by a black vertical line.

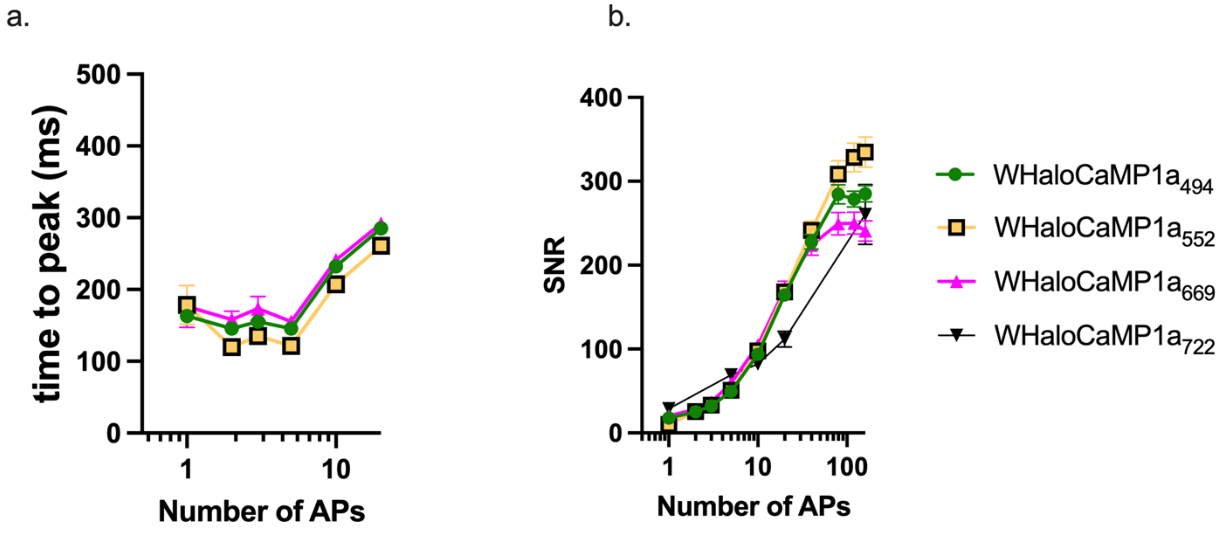

Supplementary Figure 8. Summary of properties of WHaloCaMP1a bound to different dye-ligands in a field stimulation assay in cultured neurons.

a, Time to peak (ms) from stimulation onset to max fluorescence value, imaged at 33 Hz. **b**, Signal-to-noise ratio (SNR) calculated as from the amplitude in the fluorescent change on the field stimulation divided by the standard deviation of the baseline fluorescence 1 s before each stimulus. Mean and standard error of the mean (s.e.m.) is shown. These values were calculated from data collected from at least three technical replicates across two independently seeded neuronal cultures.

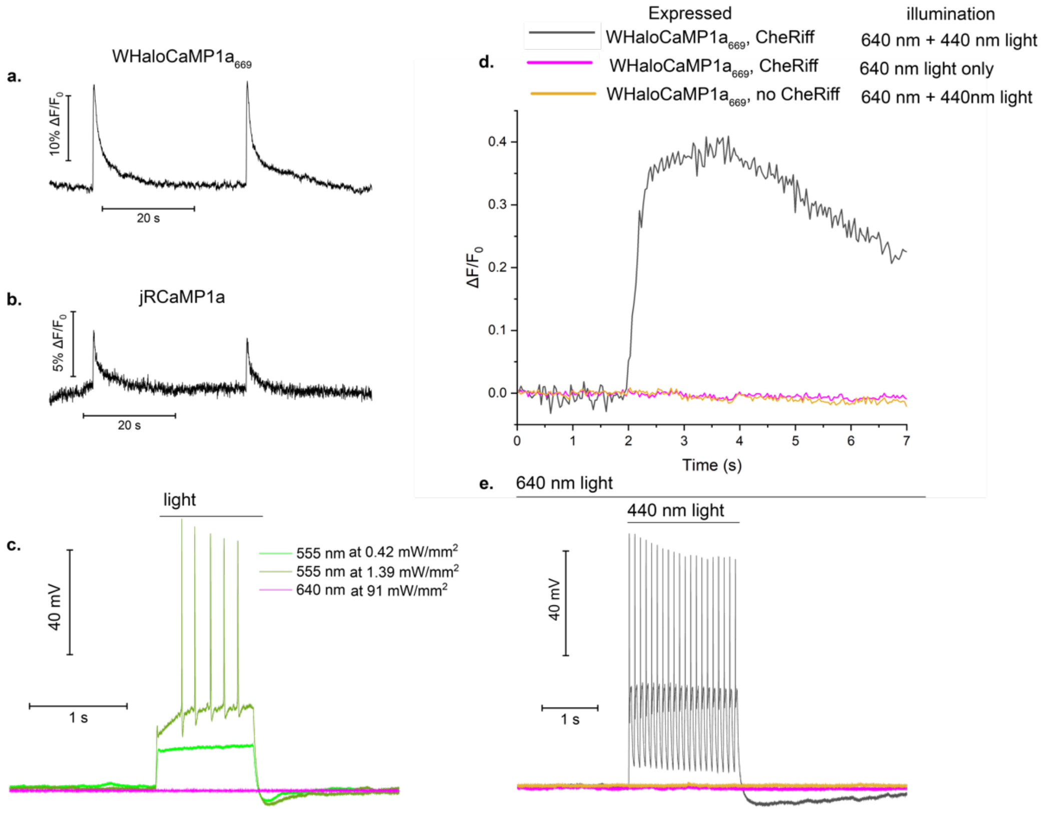

Supplementary Figure 9. WHaloCaMP1a$_{669}$ is spectrally compatible with the channelrhodopsin variant CheRiff.

Primary neuronal cultures were infected with AAV particles for WHaloCaMP1a and labeled with JF$_{669}$-HaloTag ligand, or infected with AAV particles for with jRCaMP1a, to measure spectral compatibility with the blue light-activated channel rhodopsin CheRiff via fluorescence imaging and simultaneous electrophysiology. **a**, Fluorescence trace of WHaloCaMP1a$_{669}$ after current injection, imaged with low light power (0.76 mW/mm^2, 640 nm LED). **b**, Fluorescence trace of jRCaMP1a after current injection, imaged with low light power (1.39 mW/mm^2, 555 nm LED). **c**, Voltage traces from single cell patch clamp electrophysiology of neuronal cultures expressing CheRiff, with illumination at 555 nm or 640 nm. 555 nm light depolarizes the cell at low light levels, even if it does not lead to spiking action potentials. No depolarization was observed with 640 nm illumination, even with over 10× the light power needed to image WHaloCaMP1a$_{669}$ with good SNR. **d** and **e**, WHaloCaMP1a$_{669}$ is compatible with CheRiff activation and does not show any 440 nm light dependent-photoswitching. Fluorescence trace (**d.**) and electrophysiological recoding (**e.**) are shown for the same experiment. Action potentials and changes in fluorescence are only observed when WHaloCaMP1a$_{669}$ is co-expressed with CheRiff and illuminated with 440 nm and 640 nm light (black trace). No action potentials or change in fluorescence are seen when only illuminated with 640 nm light, or when WHaloCaMP1a$_{669}$ is expressed alone.

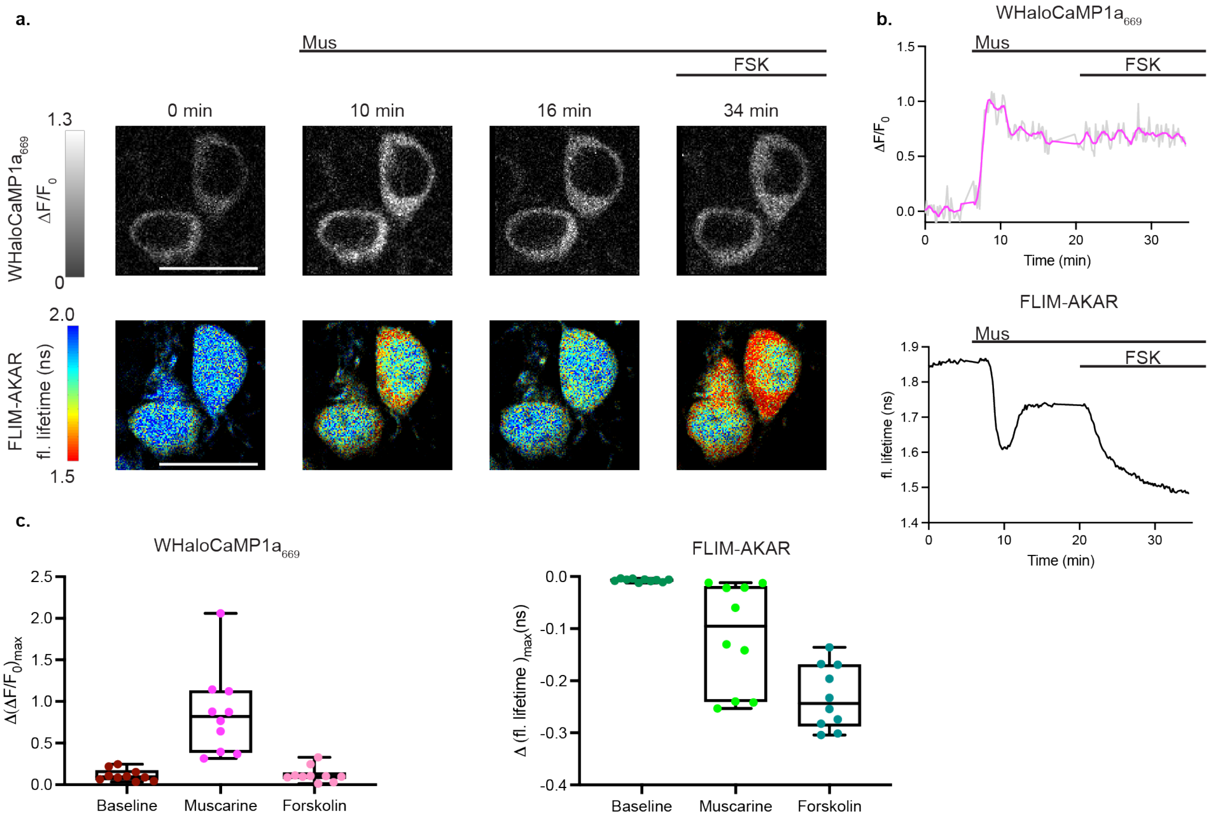

Supplementary Figure 10. Multiplexed imaging in acute brain slices with WHaloCaMP1a669 and FLIM-AKAR.

a, Heatmap showing multiplexed imaging of WHaloCaMP1a669 and FLIM-AKAR. A decrease in fluorescence lifetime in FLIM-AKAR indicates an increase in PKA activity. Top row: intensity heatmap of hippocampal CA1 neurons in acute brain slices expressing WHaloCaMP1a669, showing calcium responses to muscarine (mus, 10 µM) and subsequent forskolin (FSK, 50 µM) application. Bottom row: lifetime heatmap of FLIM-AKAR of the cells at the same time points showing PKA phosphorylation responses. Scale bar, 20 µm. **b**, Example traces of WHaloCaMP1a669 and cytoplasmic FLIM-AKAR plotted through time. The intensity and lifetime traces correspond to the top right cell in panel a. The gray intensity trace corresponds to raw $\Delta F/F_0$ values, and the trace in magenta corresponds to $\Delta F/F_0$ values smoothed through time using 6 neighboring points and a second order smoothening polynomial in GraphPad Prism. **c**, Summary of maximum responses of cells co-expressing WHaloCaMP1a669 and cytoplasmic FLIM-AKAR. N = 10 neurons from 4 mice, between ages of 15-19 days old. Each circle represents one hippocampal CA1 or cortical neuron. Each box represents median and interquartile intervals. The whiskers represent the range of data.

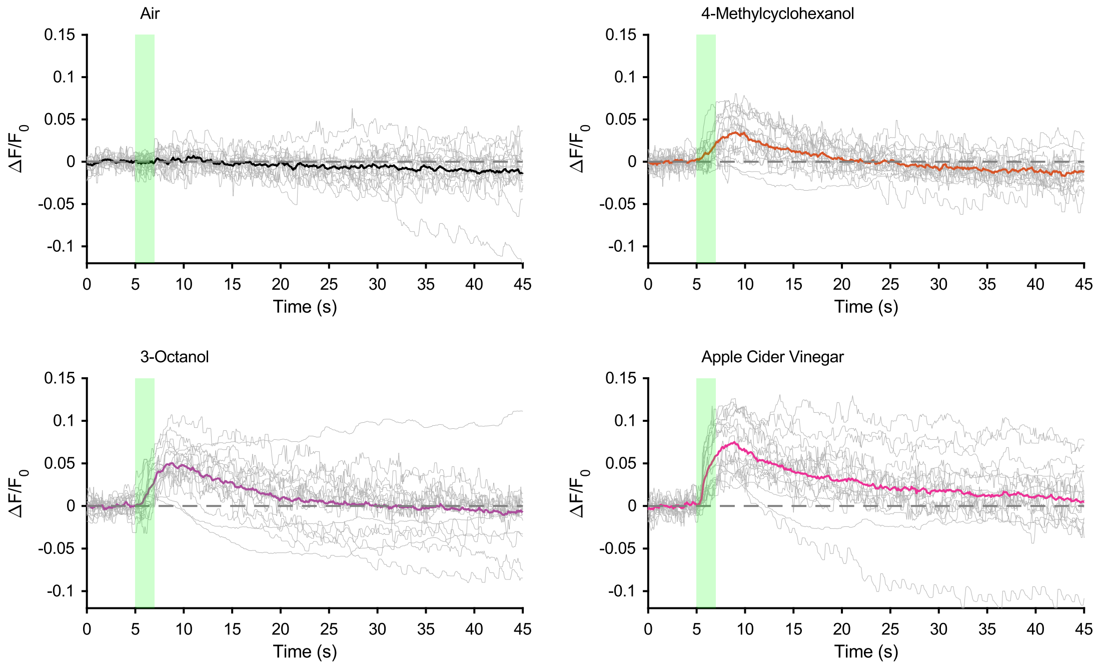

Supplementary Figure 11. Individual traces for WHaloCaMP1a$_{669}$ imaging of odor responses in flies.

The thin gray lines represent each odor presentation trial for the same dataset as depicted in Fig. 3b. The thick line denotes the mean across trials. Green shading indicates odor presentation for 2 s. A median filter with a timespan of 500 ms (5 data points) was applied.

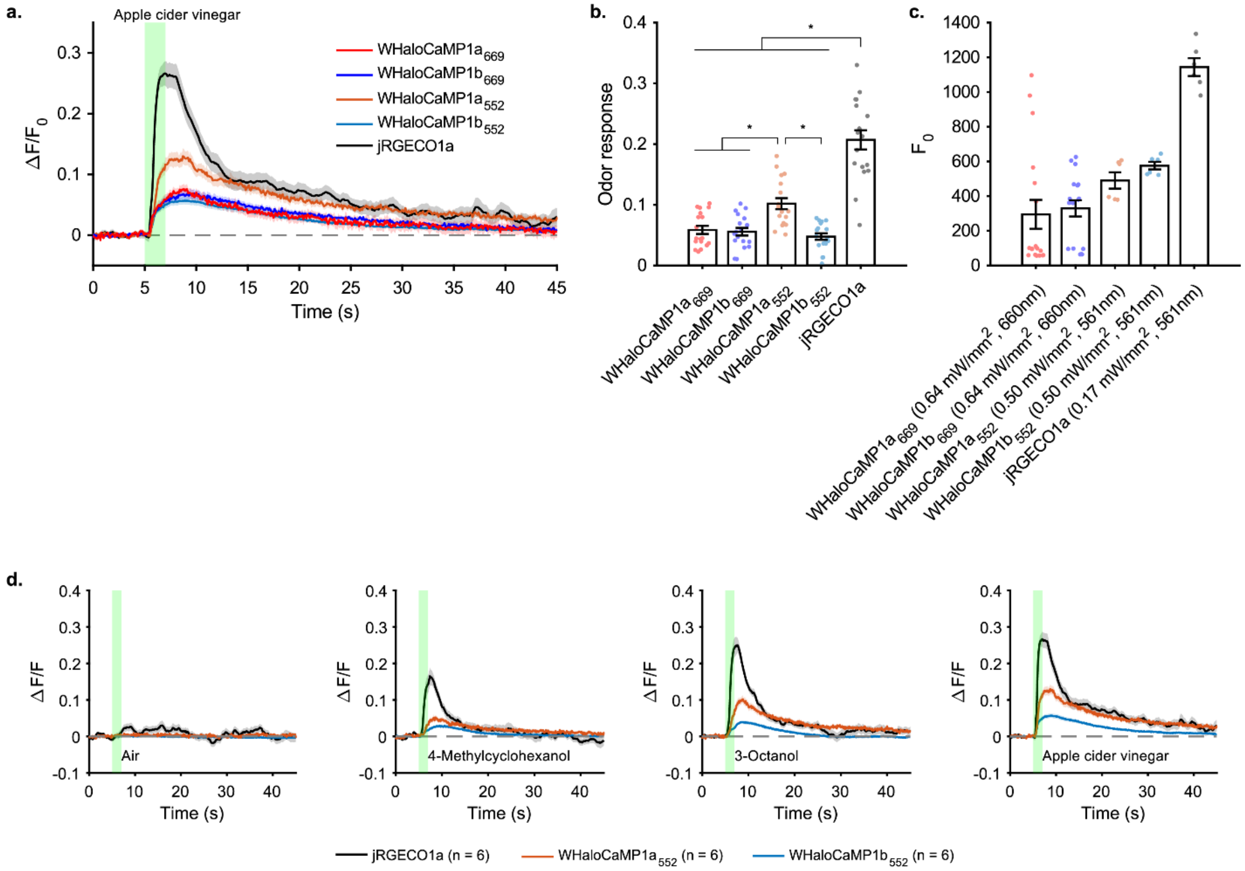

Supplementary Figure 12. Odor responses in fruit flies recorded with different WHaloCaMP variants and JF dye-ligands.

a, Fluorescence time courses to apple cider vinegar (ACV) odor. WHaloCaMP-expressing flies were loaded with JF-dye HaloTag ligands (5 µM JF_{669}-HTL or 1 µM JF_{552}-HTL) via a 1hr incubation/1hr wash-out protocol. n = 6 flies for each group and each fly received three odor presentation trials. **b**, Mean odor responses integrated during the time window from 0.5 and 5.5 seconds after odor onset. Data points are odor trials, while bars are means ± standard error of the mean (s.e.m.). *, P < 0.01 Two-sided t-test with Bonferroni correction of multiple comparisons (P values after correction, 0.006, 0.002, 0.0001 for $WHaloCaMP1a_{552}$ vs $1a_{669}$, $1b_{669}$ and $1b_{552}$, < 0.0001 for jRGECO1a vs all the other groups). Same data as in (**a.**). **c**, Resting florescence (F_0). Bars are means ± s.e.m. from odor trials. LED wavelength and intensity at the sample plane are noted. n = 6, 6, 2, 2, 2 flies for each group at the designated illumination intensities respectively; three odor trials for each fly. **d**, Responses of $WHaloCaMP1a_{552}$ and $WHaloCaMP1b_{552}$ in comparison to jRGECO1a for different odor presentations for n = 6 flies. Odors were presented three times for each fly. The thick line and shaded areas indicate mean and s.e.m. across odor trials from all 6 flies. Green shading indicates odor presentation.

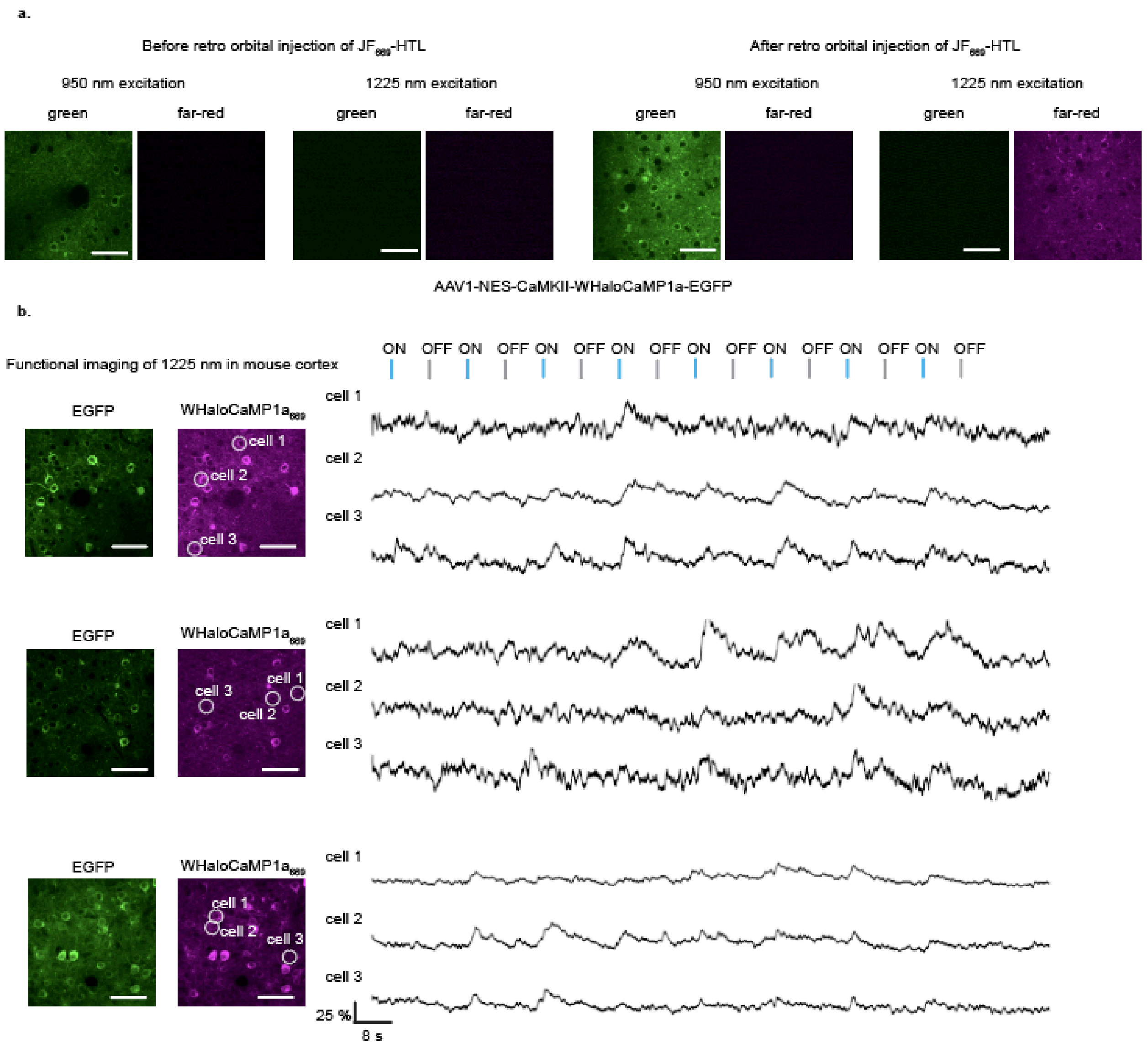

Supplementary Figure 13. Two-photon imaging of WHaloCaMP1a labeled with JF_{669}-HaloTag ligand in mouse visual cortex.

a, Two photon imaging of mouse cortex expressing WHaloCaMP1a-EGFP with excitation at 950 nm and 1225 nm before and after retro orbital injection with JF_{669}-HaloTag ligand. **b**, functional imaging of $WHaloCaMP1a_{669}$ in mouse visual cortex. Left, images of EGFP and $WHaloCaMP1a_{669}$ imaging channels and right, fluorescence $\Delta F/F_0$ traces of highlighted neurons during visual presentation stimulus in front of the mouse eye as indicated by the “ON” and “OFF” indications above the traces. All scale bars, 50 µm. All images are taken 100 µm-250 µm below the dura. The experiment was repeated three times with similar results.

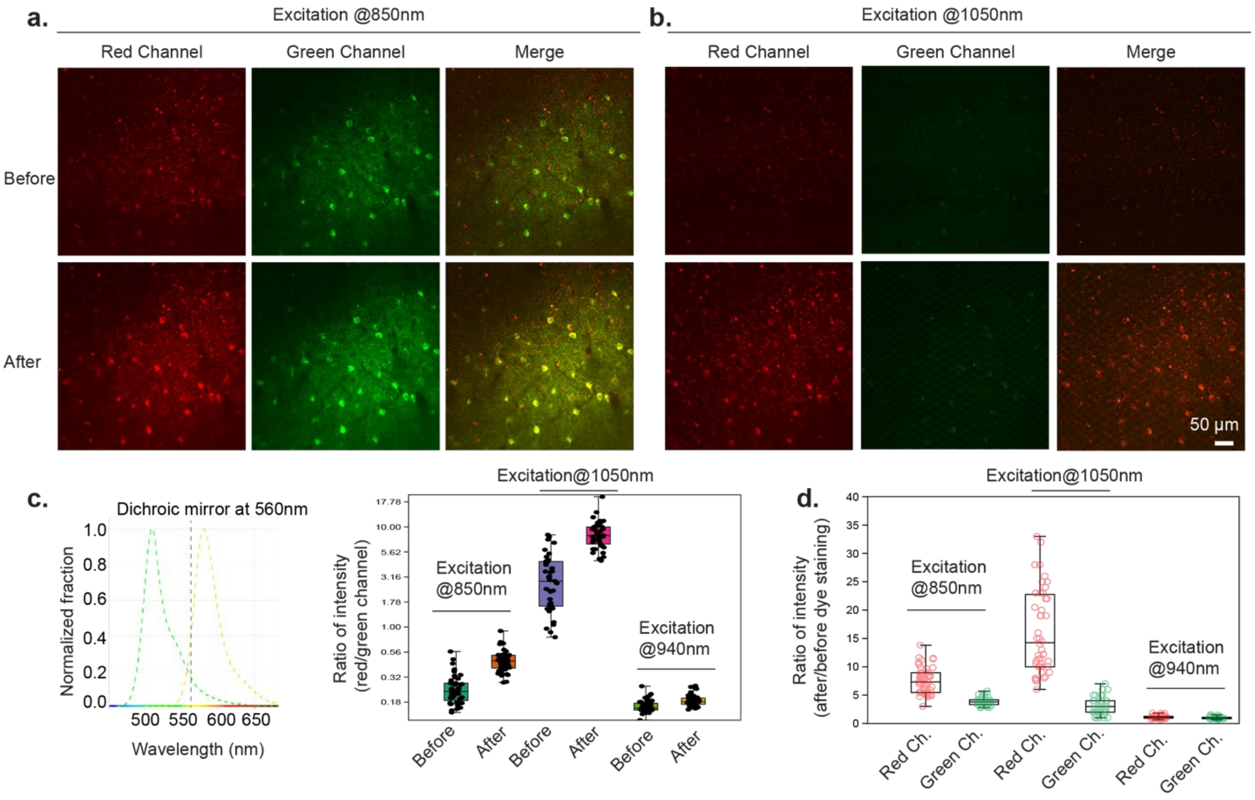

Supplementary Figure 14. Two-photon imaging of WHaloCaMP1a$_{552}$ in mouse primary visual cortex (V1).

a and **b**, Images acquired under excitation at 850 nm (**a.**) or 1050 nm (**b.**) Images of the same field-of-view were acquired before (top) and after (bottom) the injection of JF$_{552}$-HaloTag ligand. Scale bar, 50 µm. **c**, Left: Emission spectrum of EGFP and JF$_{552}$ -HaloTag ligand (https://public.brain.mpg.de/shiny/apps/SpectraViewer/). Dashed line indicates the dichroic mirror cutoff wavelength. Right: Quantification of changes in the ratio of intensity (red over green channels) before and after dye injection at 850 nm, 1050 nm, and 940 nm 2-photon excitation wavelengths. Y axis is displayed in $\log_{10}$ for better visualization of data trend. Median values/cell numbers are (from left to right): 0.23/45; 0.46/45; 2.87/39; 8.2/41; 0.16/46; 0.18/46. Each dot represents a cell. **d**, Quantification of the ratio of intensity (after dye labeling over before dye labeling) at the three different excitation wavelengths (850 nm, 1050 nm, and 940 nm). Median values/cell numbers (N number in the boxplot) are (from left to right): 7.57/48; 3.81/48; 14.25/44; 3.0/44; 1.10/49; 0.94/49. Each dot represents a cell. **c** and **d**, bounds of boxes are 25-75 percentile. Whiskers are shown in Tukey style (plus or minus 1.5 times interquartile range (IQR)).

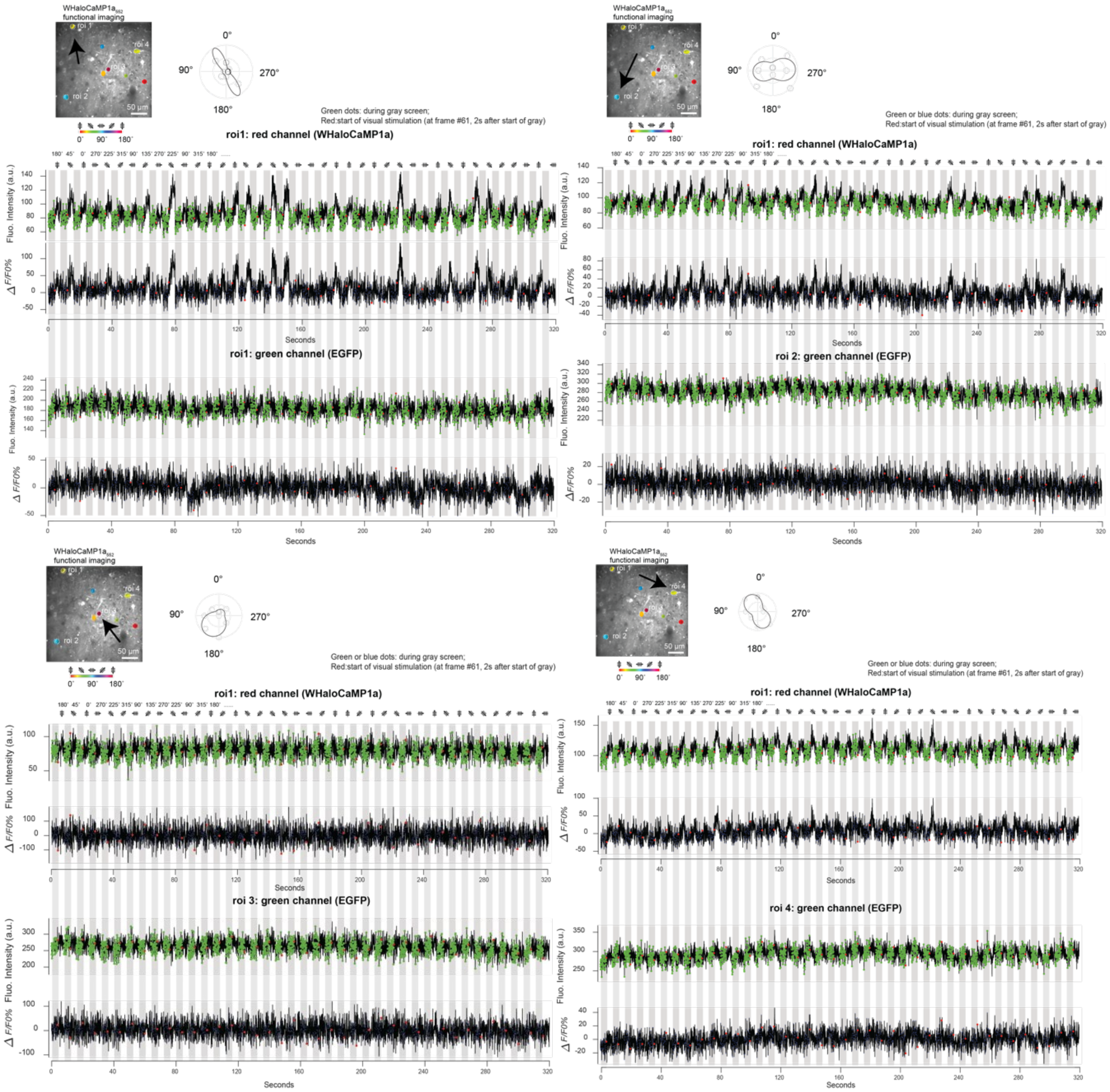

Supplementary Figure 15. Single trial and individual traces of two-photon imaging of WHaloCaMP1a$_{552}$ in mouse primary visual cortex (V1).

Four examples of single trial data from individual cells during 2-photon imaging of WHaloCaMP1a$_{552}$. Each example shows the neuron in question by a black arrow, and the orientation tuning of the neurons. Below are the red WHaloCaMP1a$_{552}$ responses and EGFP normalization channel, with both fluorescence intensity and calculated $\Delta F/F_0$ shown for each trial. The red dots indicate the start of the visual stimulation.

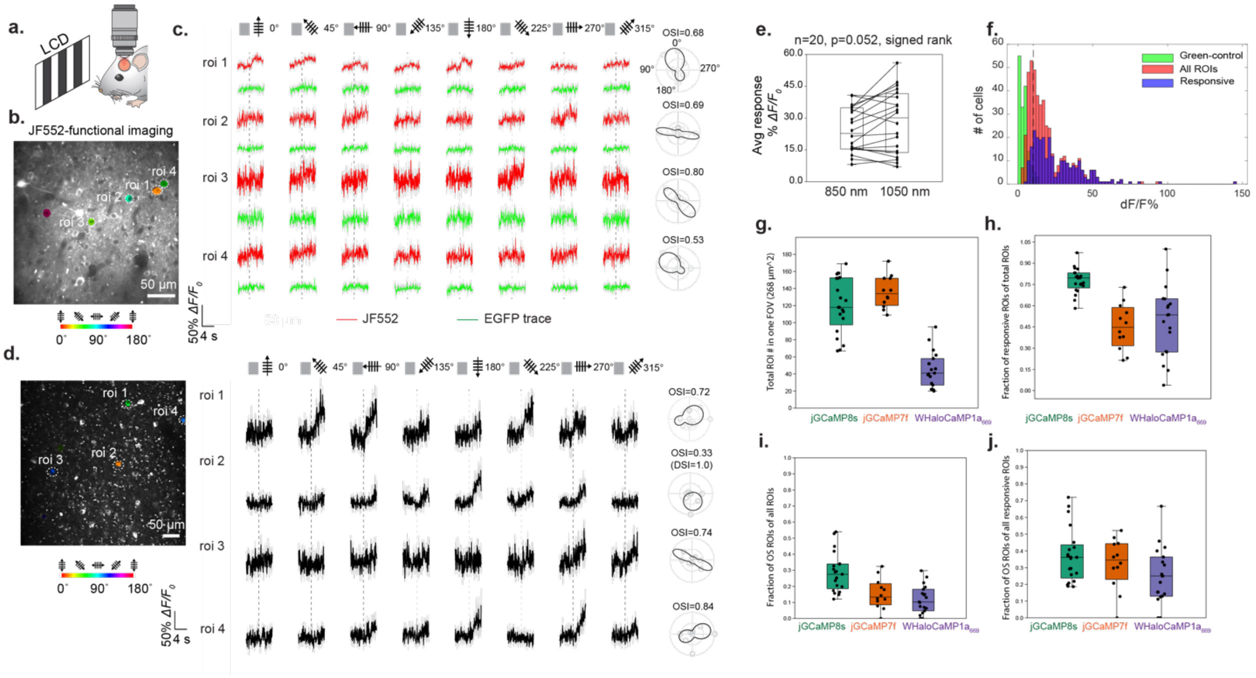

Supplementary Figure 16. Functional imaging in the mouse V1 of WHaloCaMP1a$_{552}$ and quantitative analysis.

a, Schematic of the experimental setup: the animal is anesthetized and exposed to moving grafting of different orientations. **b** and **c**, Orientations selectivity (OS) map (**b.**) and calcium (JF$_{552}$ channel) or control (EGFP channel) traces (ROIs 1-4) under 850 nm excitation, depth 148 µm, laser power 15 mW at 850 nm excitation (**c.**). **d**, OS map (left) and calcium (JF$_{552}$ channel) traces (ROIs 1-4) under 1050 nm excitation. depth 288 µm, imaging power is 139 mW at 1050 nm. Imaging rate is 15 Hz. Orientation selectivity index (OSI) of cells were shown in the right panel of (**c, d**). **e**, Side-by-side comparison of response magnitude under 850 nm and 1050 nm excitation for the same cells. N = 20 cells. p = 0.052 from two-sided Wilcoxon signed-rank test. **f**, Distribution of response magnitude in green-control channel ($\Delta F/F_0$ % as 10.2% at 95 percentile shown as dashed line, n = 160 cells from 2 mice), all ROIs in the JF$_{552}$ channel ($\Delta F/F_0$ % as 25.5% at 75 percentile, n = 470 cells from 4 mice) and responsive ROIs in JF$_{552}$ channel ($\Delta F/F_0$ % as 35.4% at 75 percentile, n = 260 cells from 4 mice). **g-j,** Comparison of three calcium indicators in terms of **g**, total number of recognizable ROI (median as 118/134/41 in the order of jGCaMP8s/ jGCaMP7f/ WHaloCaMP1a, same order in the following text); **h**, fraction of responsive ROIs (median as 0.79/0.45/0.53) ; **i**, fraction of OS cells of all ROIs (median as 0.28/0.15/0.12); **j**, fraction of OS cells of all responsive cells (median as 0.36/0.34/0.25);. Fractions of responsive or orientation-selective cells from 19 field-of-views (N number in the boxplot) in 4 mice for WHaloCaMP1a. For jGCaMP7f and jGCaMP8s, data were from 12 FOVs (N number in the boxplot) in 3 mice and from 21 FOVs (N number in the boxplot) in 5 mice, respectively, which is the same dataset as used in one of our recent publications[3]. **g to j**, bounds of boxes are 25-75 percentile. Whiskers are shown in Tukey style (plus or minus 1.5 times interquartile range (IQR)).

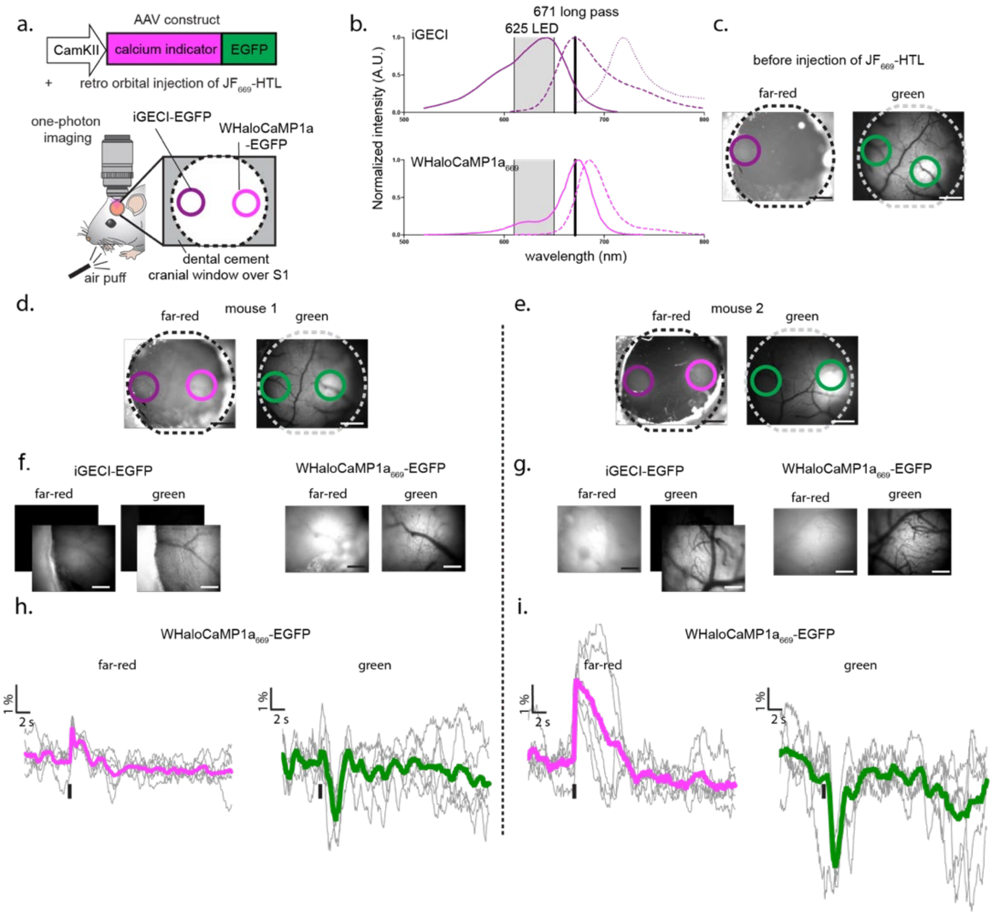

Supplementary Figure 17. One-photon imaging of WHaloCaMP1a$_{669}$-EGFP and iGECI-EGFP in mouse cortex

a, Top: schematic of adeno-associated viral (AAV) particles expression construct. bottom: schematic of one-photon imaging in the somatosensory barrel cortex (S1) of mice, indicating cranial window, dental cement, and injection positions of AAV particles. **b**, excitation and emission spectra of WHaloCaMP1a$_{669}$ and iGECI. Note that the 625 LED better fits the excitation spectrum of iGECI than WHaloCaMP1a$_{669}$. **c**, one-photon imaging of the cranial window before injection of JF$_{669}$-HTL. The purple circle in the far-red channel indicated spot of iGECI florescence. No fluorescence from the site of WHaloCaMP1a-EGFP was seen. Two green circles indicate green fluorescence from iGECI-EGFP and WHaloCaMP1a-EGFP. Round dashed circles outline edge of visible window. Scale bars, 500 µm. **d**, **e**, examples the overview of two mice expressing WHaloCaMP1a$_{669}$-EGFP and iGECI-EGFP after injection of JF$_{669}$-HTL. Scale bars, 500 µm. **f**, **g**, Imaging of spots seen in d and e with higher magnification. Imaging set-up and contrast was identical per channel, so higher contrast images of iGECI-EGFP are overlayed. Scale bars, 200 µm. **h**, **i**, one-photon imaging of WHaloCaMP1a$_{669}$-EGFP from the two mice in the far-red and green channel in response to whisker simulation indicated by black line. Individual traces from each trial and the averaged over 6 trials as a thicker line are shown. Note the negative response in the green channel from the haemodynamic response. Y axis indicated $\Delta F/F_0$. c-g. experiments were repeated more than three times with similar results.

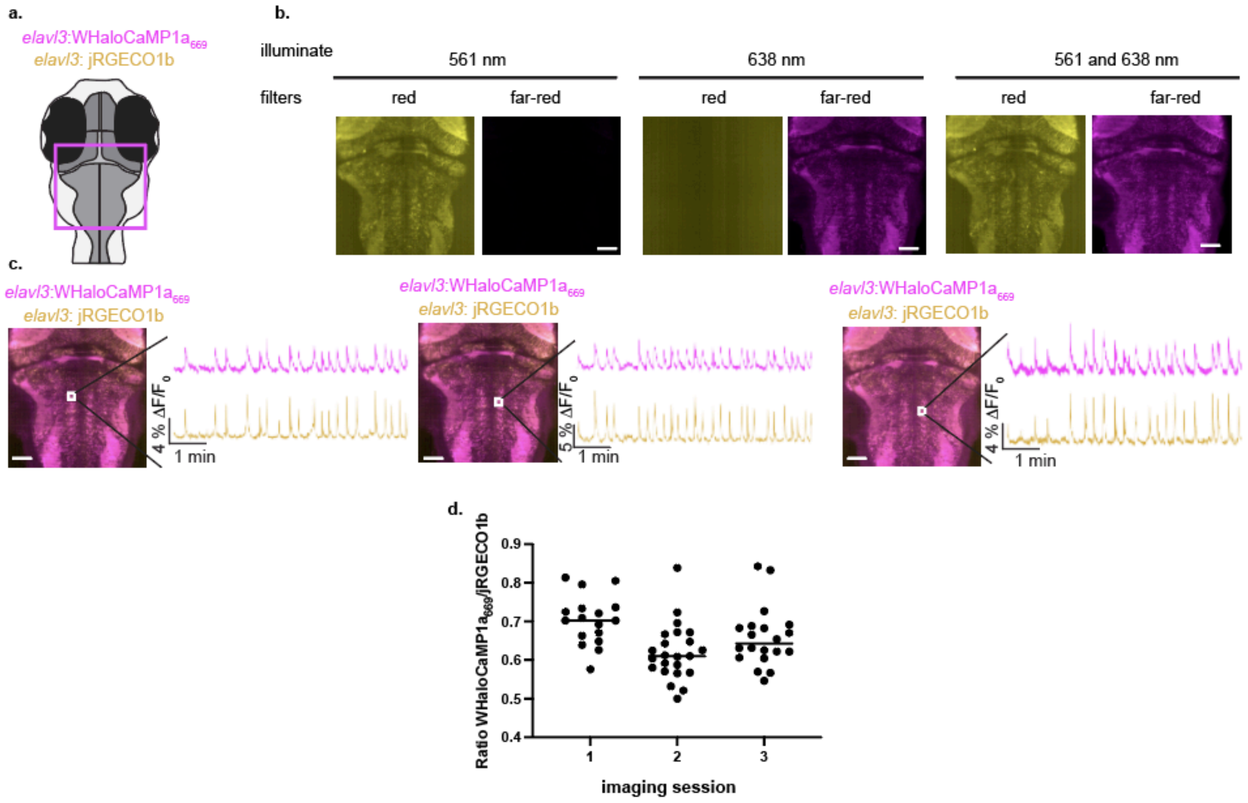

Supplementary Figure 18. Comparison of WHaloCaMP1a669 and jRGECO1b during light sheet imaging of neurons in zebrafish larvae.

a, Schematic of a zebrafish larva to indicate with a magenta box where imaging was performed. **b**, Fluorescence images of Tg(*elavl3*: NES-WHaloCaMP1a-EGFP), Tg(*elavl3*: jRGECO1b), with single laser illumination of 561 nm or 638 nm to excite jRGECO1b or WHaloCaMP1a669, respectively, or simultaneous illumination for dual color imaging. Experiment was repeated twice with similar results. **c**, Functional imaging of WHaloCaMP1a669 and jRGECO1b in neurons of zebrafish larvae with comparison of the $\Delta F/F_0$ fluorescence traces from the two channels. Three imaging sessions from two fish are shown. **d**, Quantitative comparison between the WHaloCaMP1a669 channel and jRGECO1b channel for each Ca^{2+} transient in the fluorescence traces. Each dot is a Ca^{2+} transient in the fluorescence trace. Overall, WHaloCaMP1a669 showed 66% of the jRGECO1b signal during the Ca^{2+} transients. Cellular responses from three imaging sessions from two fish were analyzed. All scale bars, 50 µm.

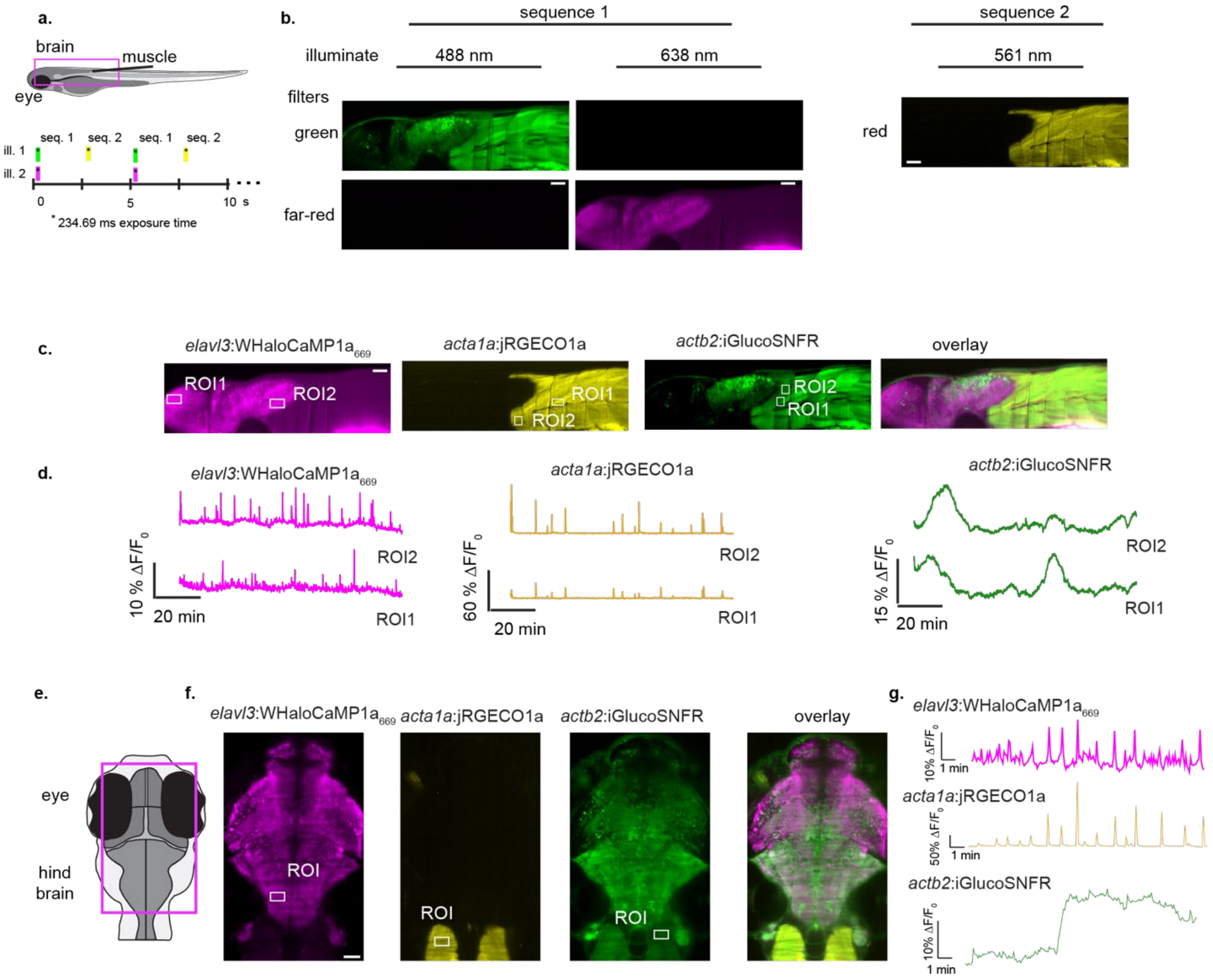

Supplementary Figure 19. Three-color functional multiplexed imaging in zebrafish larvae with WHaloCaMP1a$_{669}$, jRGECO1a and iGlucoSnFR.

a, Top: Schematic of a zebrafish larva from the side view, highlighting the eye, brain, and muscle. bellow: Illumination (ill.) scheme highlighting sequential imaging of green and far-red signals, followed by red signals with a 5s total time per frame due to a filter change on the microscope. The exposure time was 234.69 ms. **b**, Fluorescence images from sequential illumination sequences of sequence 1: 488 nm and 638 nm excitation; sequence 2: 561 nm excitation with representative images showing clear separation of the fluorescence channels. **c**, Fluorescence images of zebrafish larvae used in three color multiplexed functional imaging, with ROI for further analysis highlighted. **d**, Fluorescence $\Delta F/F_0$ traces from ROIs shown in (**c.**) ROI2 in the neuronal WHaloCaMP1a$_{669}$ channel correlates well with the muscle jRGECO1a channel, while ROI1 does not. iGlucoSnFR fluorescence changes were slower than the Ca^{2+} transients in neurons and muscle. **e**, Schematic of a zebrafish larva for three color multiplexed imaging. **f**, Fluorescence images of zebrafish larvae used in three color multiplexed functional imaging, with an ROI for further analysis highlighted. **g**, Fluorescence $\Delta F/F_0$ traces from ROI shown in (**f.**). All scale bars, 50 µm. **b, c and f**, experiments were repeated two or more times with similar results.

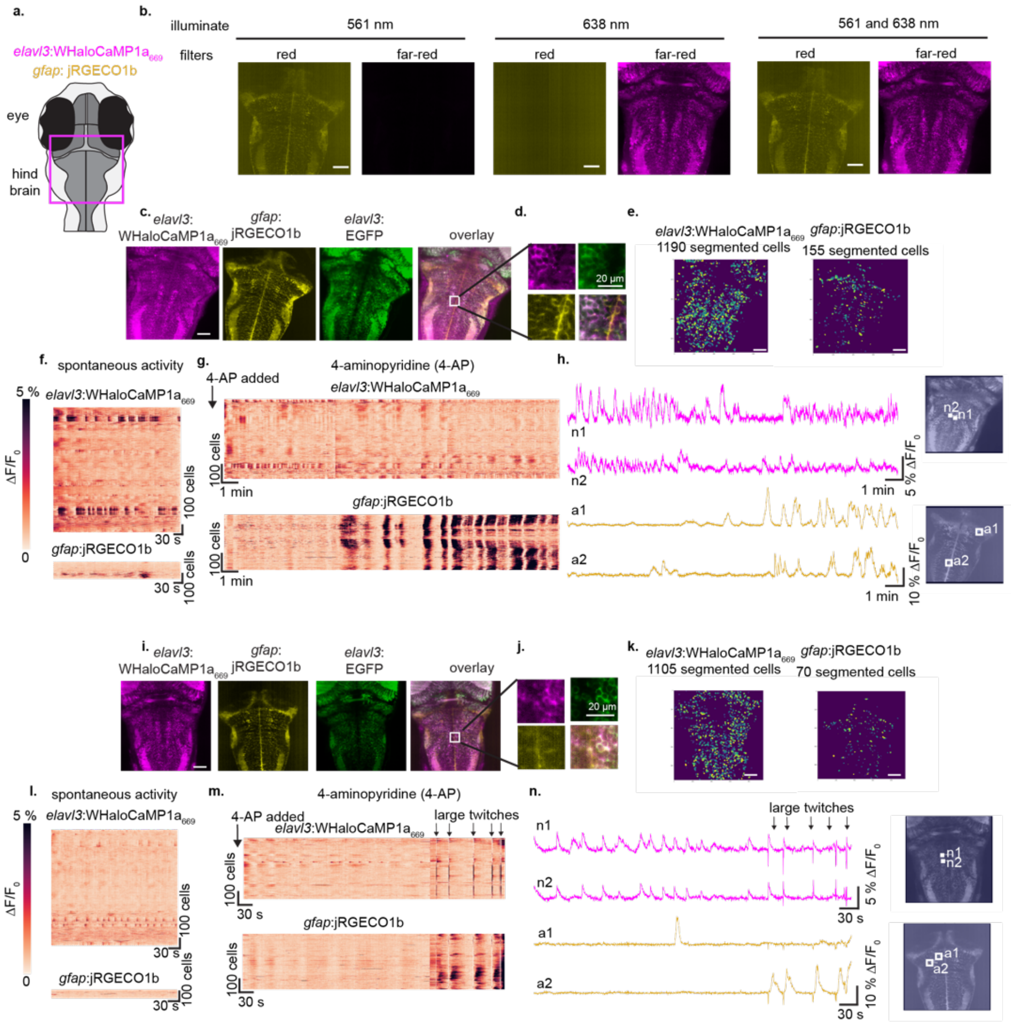

Supplementary Figure 20. Dual-color functional imaging of astrocyte and neuronal Ca^{2+} in zebrafish larvae during spontaneous activity or in the presence of 4-aminopyridine.

a, Schematic showing hind brain region where functional imaging was conducted. **b**, fluorescence images of single illumination of 561 nm or 638 nm, and dual illumination of 561 nm and 638 nm. Clear separation of channels for dual color imaging is possible. **c** and **i**, Fluorescence images of zebrafish larvae expressing NES-WHaloCaMP1a-EGFP in neurons and labeled with JF_{669}-HaloTag ligand and jRGECO1b in astrocytes. **d** and **j**, Zoom in of (**c.**) and (**i.**) showing single cell resolution. **e** and **k**, Suite2P and CellPose segmentation of dual color functional imaging. **f**, **g**, and **l**, **m**, Rasterplot of fluorescence changes in neuronal $WHaloCaMP1a_{669}$ (top) and astrocyte jRGECO1b (bottom), during spontaneous activity (**f. and l.**) or after addition of 4-AP (**g. and m.**). **h** and **n**, Two neurons and two astrocytes highlighted fluorescence $\Delta F/F_0$ trace. Functional imaging in (m.) and (**n.**) was cut short by large twitches during imaging. Unless otherwise stated, scale bars, 50 µm. The experiments were independently repeated three times with similar results.

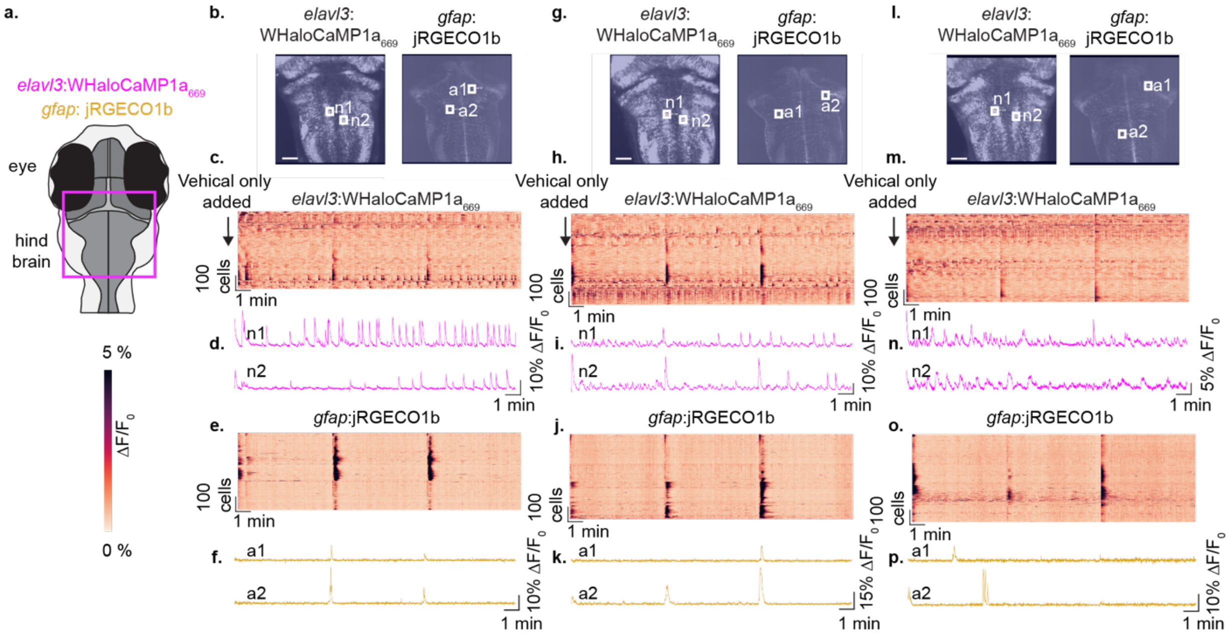

Supplementary Figure 21. Dual-color functional imaging of astrocyte and neuronal Ca^{2+} in zebrafish larvae over longer time in the absence of 4-aminopyridine.

To validate that we could follow Ca^{2+} in neurons reported by $WHaloCaMP1a_{669}$ for up to 18 minutes, we performed light sheet imaging as described in the main text on Tg(*elavl3*:NES-WHaloCaMP1a) crossed with Tg(*gfap*:jRGECO1b), but without the addition of 4-AP. **a**, Schematic of zebrafish larvae showing hind brain. **b**, **g** and **l**, images of $WHaloCaMP1a_{669}$ and jRGECO1b in three individual fish, indicating neurons and astrocyte that are later followed. **c**, **h** and **m**, Rastermap heatmap of neuronal activity as reported by $WHaloCaMP1a_{669}$. Features can be distinguished during the full 18-minute imaging time. **d**, **l** and **n**, Fluorescence $\Delta F/F_0$ traces from highlighted neurons, part of the hind brain oscillator, which we could distinguish during the full 18-minute imaging time. **e**, **j** and **o**, Rastermap heatmap of astrocyte activity as reported by jRGECO1b. No large waves of activity were seen. **f**, **k** and **p**, Fluorescence $\Delta F/F_0$ traces from highlighted astrocytes. Images were acquired as three time series of 6 min 14 s and concatenated for analysis. Scale bars, 50 µm.

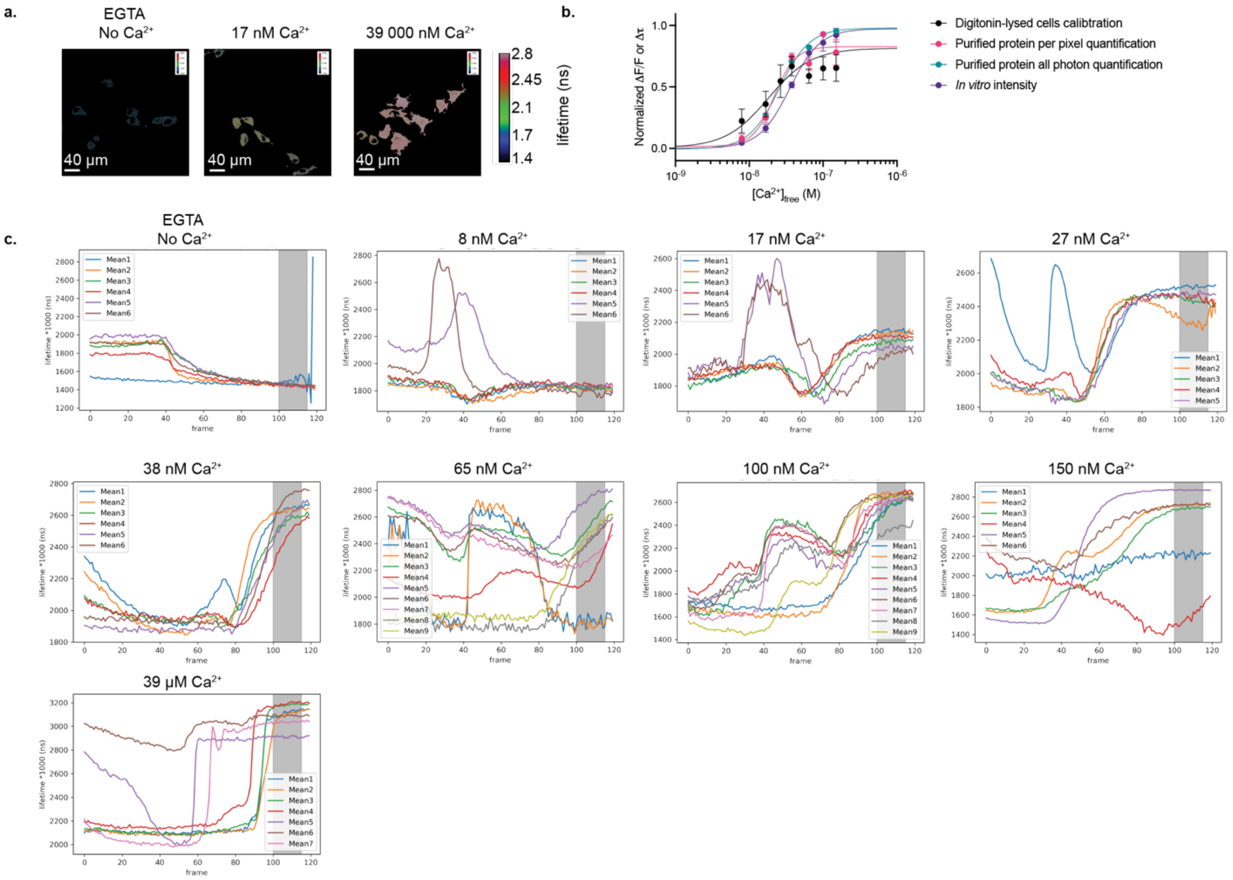

fit-parameters from calibration curves of normalized $\Delta F/F_0$ or $\Delta\tau$ vs. $[Ca^{2+}]$in (**b**.).

Fit	EC_{50} (nM)	Hill coeff.
**Digitonin-lysed cells calibration (lifetime) (black)**	18 ± 5	1.5 ± 0
**Purified protein per pixel quantification (lifetime) (pink)**	22 ± 3	3 ± 0.8
**Purified protein all photon quantification(lifetime) (green)**	26 ± 1	2.1 ± 0.1
***In vitro* intensity (purple)**	35 ± 2	2.0 ± 0.2

*the hill coefficient was constrained to greater than 1.5.

Supplementary Figure 22. Calibration of $WHaloCaMP1a_{669}$ for quantitative $[Ca^{2+}]$ determination by FLIM.

a, FLIM images from HeLa cells transfected with WHaloCaMP1a and labeled with JF_{669}-HaloTag ligand. Cells were bathed in known concentration of free calcium solution and permeabilized by digitonin. Three representative images are shown for No Ca^{2+}, 17 nM Ca^{2+} and 39 µM Ca^{2+}. Scalebar, 40 µm. **b**, Fluorescence lifetime vs. $[Ca^{2+}]$ titration calibration curves from digitonin lysed cells (black), purified protein per pixel lifetime fit (pink), purified protein all-photon lifetime fit (green), or purified protein plate reader intensity experiment. All titrations are normalized to max value within the titration curve. Mean and s.d. are plotted. Fit values from the titrations curve in the table below the images. **c**, Quantified lifetime from cells after permeabilization with digitonin and bathed in indicated concentration of free calcium. Shaded area indicates region which was used to generate the calibration curve. Representative example from three independent calibration experiments.

To calculate the [Ca^{2+}] (x, M) from a measured lifetime (y, ns, scaled by 1000) for images acquired under imaging parameters used for the HeLa cell calibration experiment:

$$x = EC_{50}\left(\frac{a-y}{y-b}\right)^{\left(\frac{1}{h}\right)}$$

a (*1000) (ns)	b (*1000) (ns)	EC_{50} (M)	h
1300	2900	1.6×10^{-8}	1.5

Where (a) is the value of fluorescence at the bottom of the calibration curve, (b) is the value of fluorescence at the top of the curve, (EC_{50}) is the concentration of agonist that gives a response halfway between bottom and the top, and (h) is the hill or cooperative coefficient.

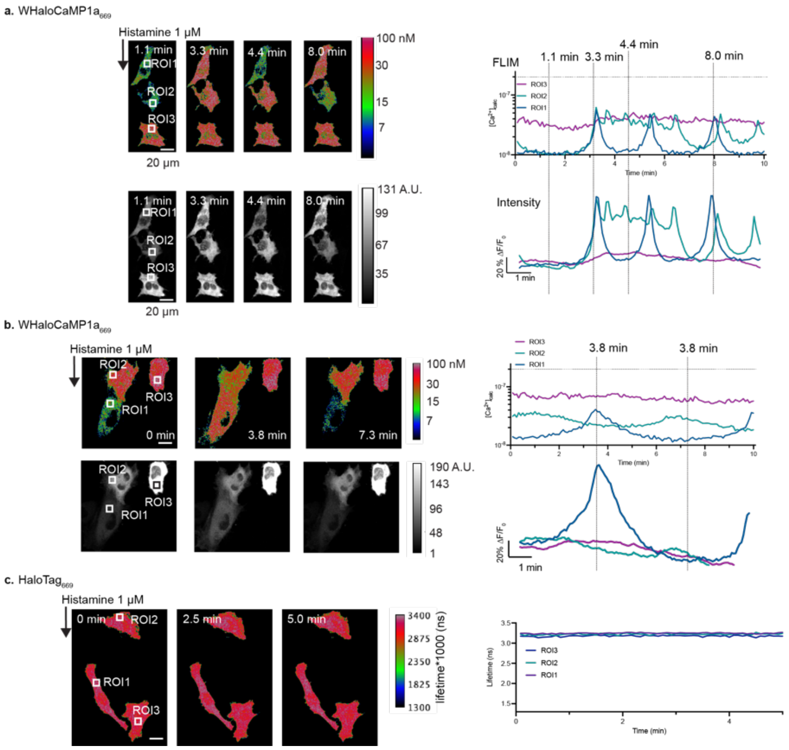

Supplementary Figure 23. WHaloCaMP1a$_{669}$ as a FLIM probe in HeLa cells.

Equation used to calculate [Ca^{2+}] from a given lifetime (scaled by 1000 ns), as well as the fit constant values used, derived from in-cell titration with permeabilized HeLa cells and known free Ca^{2+} solutions. **a** and **b**, Two further examples of WHaloCaMP1a$_{669}$ in HeLa cells used to determine Ca^{2+} concentrations during histamine-induced oscillations. Example images of the lifetime images converted to [Ca^{2+}] (top) and intensity images (bottom) to the left, as well as traces of calculated [Ca^{2+}] from the fluorescence lifetime and fluorescence $\Delta F/F_0$ traces for three ROIs highlighted in the images. The maximum concentration of [Ca^{2+}] that could be quantified is indicated by a horizontal line on the y-axis (200 nM), and vertical lines indicate time points of images in the left representative images. **c**, HaloTag$_{669}$ expressed in HeLa cells and stimulated with histamine shows no changes in fluorescence lifetime. Scale bar, 20 µm.

To calculate the $[Ca^{2+}]$ (x, M) from a measured lifetime (y, ns, scaled by 1000 ns) for the images acquired under images parameters *for in vivo* zebrafish larvae imaging:

$$x = EC_{50}\left(\frac{a-y}{y-b}\right)^{\left(\frac{1}{\hbar}\right)}$$

a (*1000) (ns)	b (*1000) (ns)	EC_{50} (M)	h
1400	2900	3.8×10^{-8}	2.1

Where (a) is the value of fluorescence at the bottom of the calibration curve, (b) is the value of fluorescence at the top of the curve, (EC_{50}) is the concentration of agonist that gives a response halfway between bottom and the top, and (h) is the hill or cooperative coefficient.

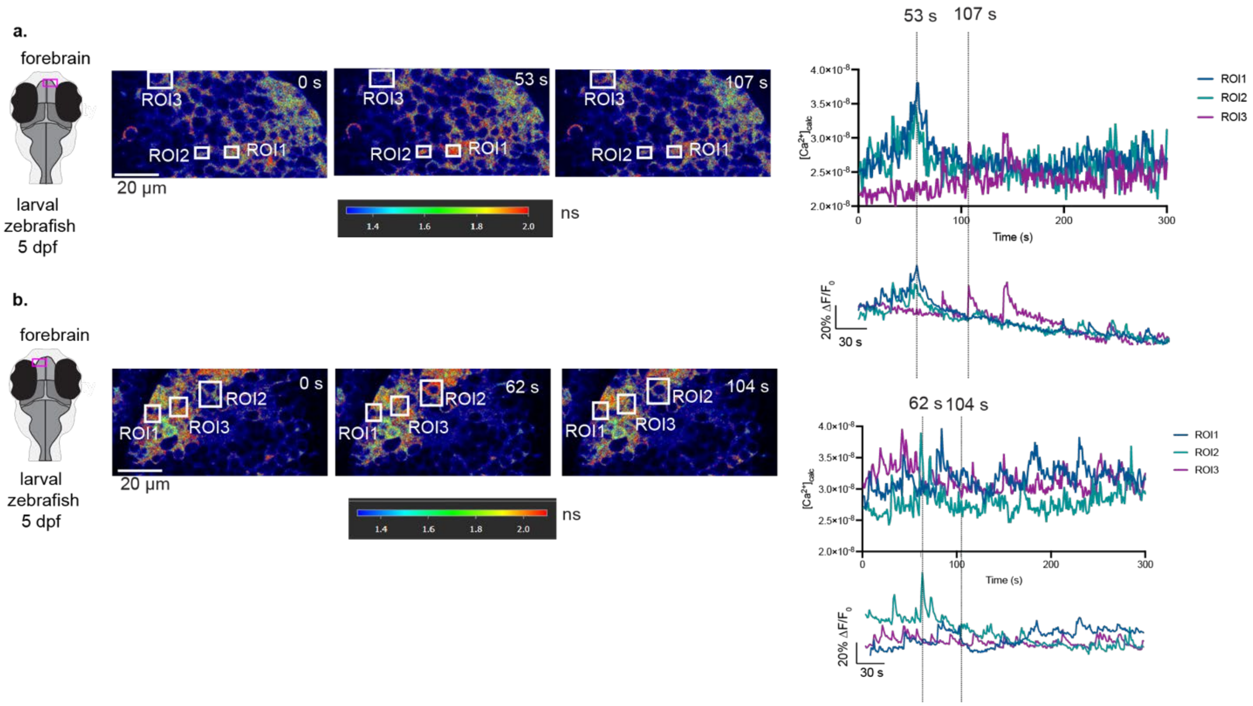

Supplementary Figure 24. *In vivo* quantitative FLIM for $[Ca^{2+}]$ in zebrafish larvae.
Equation used to calculate $[Ca^{2+}]$ from a given lifetime (scaled by 1000 ns), as well as the fit constant values used, derived from purified protein calibration at these image settings. **a** and **b**, Two further examples of in vivo FLIM to determine $[Ca^{2+}]$ in zebrafish larvae on the single cell resolution. Tg(*elavl3*:NES-WHaloCaMP1a-EGFP) was labeled with JF_{669}-HaloTag ligand and zebrafish larvae mounted on an inverted FLIM set-up, with zoom in on the forebrain as indicated in the schematic. FLIM images and intensity images overlaid in the Leica LASX software are shown, with the color bar for lifetime. Analysis was done on raw images of lifetime, which were used to calculate the $[Ca^{2+}]$ from a calibrated Ca^{2+} titration in purified protein. $[Ca^{2+}]$ calculated from fluorescence lifetimes (top) in ROIs highlighted in the images, as well as fluorescence $\Delta F/F_0$ traces (bottom) are shown. Dashed lines indicated time points at which representative images are shown. Scale bar, 20 µm. The experiment was independently repeated three times with similar results.

Supplementary Table 1. X-ray crystallography structural and refinement statistics

	HaloTag7$_{669}$ (PDB 8SW8)
**Data collection**	
Space group	$P4_32_12$
Cell dimensions	
a, *b*, *c* (Å)	62.74, 62.74, 163.55
α, β, γ (°)	90, 90, 90
Resolution (Å)	58.65-1.90 (1.94-1.90)
*R*merge	0.121(1.341)
I / σ*I*	23.0 (4.0)
Completeness (%)	99.6 (98.9)
Redundancy	26.3 (27.1)
**Refinement**	
Resolution (Å)	58.65-1.90
No. reflections	26365
R_{work} / R_{free}	0.193, 0.246
No. atoms	
Protein	4635
Dye-HaloTag ligand	96
Chloride ion	1
Water	173
B-factors	
Protein	23.4
Dye-HaloTag ligand	44.3
Chloride ion	19.2
Water	29.1
R.m.s. deviations	
Bond lengths (Å)	0.010
Bond angles (°)	1.662

*Structure was determined for one crystal.

**Values in parentheses are for highest-resolution shell.

	Ex/Em$_{apo}$ (nm)	Ex/Em$_{sat}$ (nm)	Dynamic range (F_{max}/F_{min})	K_d (nM)	Hill coeff.	ε_{apo} (x1000) (M^{-1} cm^{-1})	ε_{sat} (x1000) (M^{-1} cm^{-1})	Φ_{apo}	Φ_{sat}	Brightness$_{apo}$ (mM^{-1} cm^{-1})	Brightness$_{sat}$ (mM^{-1} cm^{-1})
WHaloCaMP1a$_{669}$	677/688	679/690	7	37 ± 2	2.5 ± 0.3	127	174	0.08	0.50	10.1	87.0
NIR-GECO1*	678/704	678/704	($\Delta F/F_{min}$) -9	885	1.0	69	20	0.06	0.02	4.1	0.4
NIR-GECO2*	678/704	678/704	($\Delta F/F_{min}$) -15	331	0.9	67	18	0.06	0.01	4.0	0.3
NIR-GECO2G*	678/704	678/704	($\Delta F/F_{min}$) -9	480	0.8	74	21	0.06	0.02	4.5	0.4
iBB-GECO1*	648/668	646/653	($\Delta F/F_{min}$) -13	105	1.9	52	16	0.11	0.03	6.2	0.5
GAF-CaMP2-sfGFP°	630/676	642/674	($\Delta F/F_{min}$) 0.9	289	1.53°°	16	28	0.03	0.06	0.5	1.6
GAF-CaMP3-sfGFP°°	636/674	648/676	($\Delta F/F_{min}$) 2	433	1.36	14	27	0.05	0.08	0.7	2.1
iGECI#	donor 640/670 acceptor 702/720	donor 640/670 acceptor 702/720	6	15 and 890	2.5 and 0.9	-	-	-	-	12.3	2.0
iGECInano##	donor 645/670 acceptor 702/720	donor 640/670 acceptor 702/720	4	530	1.53	-	-	-	-	-	-
jGCaMP8s###	488/510	488/510	($\Delta F/F_{min}$) 46	46± 1	2.2 ± 0.1	2.1	57	0.47	0.53	1.0	30.2

Supplementary Table 2. Comparison of the photophysical properties of WHaloCaMP1a$_{669}$ and biliverdin-binding protein-based calcium indicators.

Summary of measured photophysical properties of purified protein. apo = in presence of EGTA. sat = in presence of saturating Ca^{2+}. Ex = excitation maximum. Em = emission maximum. K_d = estimated dissociation constant to Ca^{2+} or effective concentration$_{50}$ (EC_{50}) from titrations curves. Hill coeff = Hill coefficient. ε = molar extinction coefficient. Φ = quantum yield. Brightness (mM^{-1} cm^{-1}) = the product of molar extinction coefficient × quantum yield, divided by 1000.

*from (ref)[4]
°from (ref)[5]
°°from (ref)[6]
#from (ref)[7]
##from (ref)[8]
###From (ref)[3]

1AP

Protein scaffold	Dye/ supplement	Number of cells	ΔF/F$_0$ (%) ± s.e.m	SNR	Time to peak (ms)	Decay $t_{1/2}$ (s) 1st comp. (Percentage 1st comp.) 2nd comp.
**WHaloCaMP1a**						
	JF_{494}-HTL	154	20 ± 4	18 ± 1	163 ± 5	0.4 ± 0.0 (59 ± 1%) 3.3 ± 0.1
	JF_{552}-HTL	165	4.4 ± 0.2	20 ± 1	178 ± 27	0.4 ± 0.0 (60 ± 1%) 5.7 ± 0.2
	JF_{669}-HTL	130	7.0 ± 0.4	20 ± 1	176 ±29	0.5 ± 0.1 (68 ± 1%) 5.1 ± 0.3
	JF_{722}-HTL*	21	13 ± 6	29 ± 3	*	1.0 ± 0.2 (55 ± 1%) 11 ± 1
**iGECI**	with biliverdin (25 µM)	In our hands (106)	-0.8 ± 0.01	5± 1	1140±10	#
**iGECI**	with biliverdin (25 µM)	Reported from literature**	-12.9	-	700	14 (100%)
**iGECInano**	with biliverdin (25 µM)	Reported from literature***	-21.7	-	700	2.4 (100%)

*imaged at 10 Hz which is too slow to accurately capture time to peak.
# not calculated as imaging parameters were not set up for capturing slow kinetics
SNR = signal to noise ratio.
**from (ref)[7]
***from (ref)[8]

160AP

Protein scaffold	Dye/ supplement	Number of cells	ΔF/F$_0$ (%) ± s.e.m
**WHaloCaMP1a**			
	JF_{494}-HTL	153	288 ± 0.1
	JF_{552}-HTL	168	60 ± 0.03
	JF_{669}-HTL	141	77 ± 0.03
	JF_{722}-HTL*	34	80 ± 0.03
**iGECI**	with biliverdin (25 µM)	In our hands (106)	-6.0 ± 0.5
**iGECI#**	with biliverdin (25 µM)	Reported from literature#	-30.4

#from (ref)[7]

Supplementary Table 3. Fluorescence response of WHaloCaMP1a or biliverdin-binding protein-based calcium indicators in a primary neuron culture field stimulation assay

Mean and standard error of the mean (s.e.m.) is reported for the number of neurons stated. HTL stands for HaloTag ligand.

WHaloCaMP1a$_{494}$

Number AP	Traces fit	Percentage fast % ± s.e.m	$t_{1/2}$ fast (s) ± s.e.m. (s)	$t_{1/2}$ slow (s) ± s.e.m. (s)	Weighted average $t_{1/2}$ (s)
**1**	147	59 ± 1	0.4 ± 0.0	3.3 ± 0.1	1.6
**2**	152	62 ± 1	0.4 ± 0.0	4.1 ± 0.1	1.8
**3**	153	63 ± 1	0.4 ± 0.0	4.5 ± 0.1	1.9
**5**	153	65 ± 1	0.4 ± 0.0	4.8 ± 0.1	1.9
**10**	152	67 ± 1	0.4 ± 0.0	4.9 ± 0.1	1.9
**20**	146	67 ± 1	0.4 ± 0.0	4.9 ± 0.1	1.9
**40**	120	65 ± 1	0.5 ± 0.0	4.9 ± 0.1	2.0

WHaloCaMP1a$_{552}$

Number AP	Traces fit	Percentage fast % ± s.e.m	$t_{1/2}$ fast (s) ± s.e.m. (s)	$t_{1/2}$ slow (s) ± s.e.m. (s)	Weighted average $t_{1/2}$ (s)
**1**	138	60 ± 1	0.4 ± 0.0	5.9 ± 0.3	2.6
**2**	155	60 ± 1	0.4 ± 0.0	5.7 ± 0.2	2.5
**3**	158	58 ± 1	0.4 ± 0.0	5.1 ± 0.2	2.4
**5**	164	56 ± 1	0.4 ± 0.0	4.9 ± 0.1	2.4
**10**	167	57 ± 1	0.4 ± 0.0	5.1 ± 0.1	2.4
**20**	167	56 ± 1	0.5 ± 0.0	5.0 ± 0.1	2.5
**40**	106	51 ± 1	0.5 ± 0.0	5.5 ± 0.1	3.0

WHaloCaMP1a$_{669}$

Number AP	Traces fit	Percentage fast % ± s.e.m	$t_{1/2}$ fast (s) ± s.e.m. (s)	$t_{1/2}$ slow (s) ± s.e.m. (s)	Weighted average $t_{1/2}$ (s)
**1**	118	68 ± 1	0.5 ± 0.1	5.1 ± 0.3	2.0
**2**	128	68 ± 1	0.3 ± 0.0	4.0 ± 0.1	1.5
**3**	129	66 ± 1	0.4 ± 0.0	4.0 ± 0.1	1.6
**5**	132	64 ± 1	0.5 ± 0.0	4.3 ± 0.1	1.8
**10**	141	62 ± 1	0.4 ± 0.0	4.3 ± 0.1	1.8
**20**	141	58 ± 1	0.4 ± 0.0	4.3 ± 0.1	2.0
**40**	141	54 ± 1	0.5 ± 0.0	4.6 ± 0.4	2.4

WHaloCaMP1a$_{722}$

Number AP	Traces fit	Percentage fast % ± s.e.m	$t_{1/2}$ fast (s) ± s.e.m. (s)	$t_{1/2}$ slow (s) ± s.e.m. (s)	Weighted average $t_{1/2}$ (s)
**1**	23	55 ± 1	1.0 ± 0.2	11 ± 1	5.5
**5**	21	46 ± 1	0.8 ± 0.0	10 ± 1	5.7
**10**	34	41 ± 2	0.7 ± 0.0	8.3 ± 0.5	5.1
**20**	31	42 ± 1	1.0 ± 0.1	10 ± 1	6.2

Supplementary Table 4. Decay properties calculated for WHaloCaMP1a bound to different dyes in a field stimulation assay in cultured neurons.

Decay was calculated from the end of the stimulus given. Mean and standard error of the mean (s.e.m.) is reported for the number of neurons stated.

Supplementary Table 5. Representative Illumination imaging parameters for WHaloCaMP1a

	Dye	Illumination source (laser frequency)	objective	Illumination wavelength (nm)	Power at objective (mW) or irradiance (mW mm^{-2})
Purified protein, bleaching	JF_{669}-HTL	SPECTRA X light engine LED	40× oil objective (NA = 1.3, PLAN Flour, Nikon)	640	23 mW mm^{-2}
Neuronal field stimulation widefield	JF_{494}-HTL	SPECTRA X light engine LED	20× air objective (NA = 0.75, Nikon)	485	4 mW mm^{-2}
Neuronal field stimulation widefield	JF_{552}-HTL	SPECTRA X light engine LED	20× air objective (NA = 0.75, Nikon)	550	6 mW mm^{-2}
Neuronal field stimulation widefield	JF_{669}-HTL	SPECTRA X light engine LED	20× air objective (NA = 0.75, Nikon)	640	3 mW mm^{-2}
Neuronal electrophysiology and optogenetics widefield	JF_{669}-HTL	SPECTRA X light engine LED	20× air objective (NA = 0.75, Nikon)	640	0.76 mW mm^{-2}
Mouse acute slice 2-photon	JF_{669}-HTL	Spectra-Physics, Insight X3 (80 MHz)	Olympus , 60X objective, NA 1.1	1250	3.5 mW
Drosophila mushroom body, one-photon	JF_{552}-HTL	FG-OBC-561, Sutter, LED	60×, NA 1.0, water-immersion objective (LUMPlanFl/IR; Olympus)	561	0.50 mW mm^{-2}
Drosophila mushroom body, one-photon	JF_{669}-HTL	FG-OBC-660, Sutter. LED	60×, NA 1.0, water-immersion objective (LUMPlanFl/IR; Olympus)	660	0.64 mW mm^{-2}
Mouse cortex, two-photon	JF_{669}-HTL	Coherent Discovery NX TPC (80 MHz)	Olympus XLUMPFLN objective, 20x, 1.0 NA	1225	5 mW to 15 mW
Mouse cortex, two-photon	JF_{552}-HTL	Chameleon Discovery, Coherent (80 MHz)	Olympus 25×, 1.05 NA objective	850	15 mW
Mouse cortex, two-photon	JF_{552}-HTL	Chameleon Discovery, Coherent (80 MHz)	Olympus 25×, 1.05 NA objective	1050	92 to 140 mW
Mouse cortex, one-photon	JF_{669}-HTL	Thorlabs LED4D067	Nikon 10X Plan Fluor, 0.3 NA, and 3.5 mm working distance	625	7 mW mm^{-2}
Zebrafish, SimView light sheet	JF_{669}-HTL	One-photon Laser	Nikon 16x/0.8 NA water-dipping objective	685	160 mW mm^{-2}
Zebrafish, Z1 light sheet	JF_{669}-HTL	Zeiss Z.1 lasers	Objective W Plan-Apochromat 10x/0.5 M27 75mm illumination objective and two 5x/0.1 detection objectives	638	0.1 mW to 0.7 mW
FLIM, Falcon module on a Leica Stellaris microscope	JF_{669}-HTL	Leica Microsystems white light laser (40 or 80 MHz)	20× water immersion objective (Leica, HC PL IRAPO, NA = 0.75)<br>Or 25x water immersion objective (Leica, HC FLUOTAR L NA = 0.95)	671	<0.1 mW

Supplementary sequences of WHaloCaMPs

WHaloCaMP1a

MAEIGTGFPFDPHYVEVLGERMHYVDVGPRDGTPVLFLHGNPTSSYVWRNIIPHVAPTHRCIAPDLIGMGK
SDKPDLGYFFDDHVRFMDAFIEALGLEEVVLVIHDWGSALGFHWAKRNPERVKGIAFMEFIRPIPTWDEWP
EFARETFQAFRTTDVGRKLIIDQNVFIEWTLPMAVARARRKWQKTGHAVRAIGRLSSGGSGGSGGSGGSDQ
LTEEQIAEFKEAFSLFDKDGDGTITTKELGTVMRSLGQNPTEAELQDMINEVDADGNGTIDFPEFLTMMARK
MKDTDSEEEIREAFRVFDKDGNGYISAAELRHVMTNLGEKLTDEEVDEMIREADIDGDGQVNYEEFVQMM
TAKYLTEVEMDHYREPFLNPVDREPLWRFPNELPIAGEPANIVALVEEYMDWLHQSPVPKLLFWGTPGVLIP
PAEAARLAKSLPNCKAVDIGPGLNLLQEDNPDLIGSEIARWLSTLEISG

HaloTag,MLCK-peptide, Linker, Calmodulin, HaloTag
Rationally placed tryptophan: G171W
Hits from directed evolution screen: G176A, V178A, P180Y

WHaloCaMP with eNOS peptide

MAEIGTGFPFDPHYVEVLGERMHYVDVGPRDGTPVLFLHGNPTSSYVWRNIIPHVAPTHRCIAPDLIGMGK
SDKPDLGYFFDDHVRFMDAFIEALGLEEVVLVIHDWGSALGFHWAKRNPERVKGIAFMEFIRPIPTWDEWP
EFARETFQAFRTTDVGRKLIIDQNVFIEWTLPMAVIRRKKTFKEVANAVKISASLMGGGSGGSGGSGGSDQL
TEEQIAEFKEAFSLFDKDGDGTITTKELGTVMRSLGQNPTEAELQDMINEVDADGNGTIDFPEFLTMMARK
MKDTDSEEEIREAFRVFDKDGNGYISAAELRHVMTNLGEKLTDEEVDEMIREADIDGDGQVNYEEFVQMM
TAKTLTEVEMDHYREPFLNPVDREPLWRFPNELPIAGEPANIVALVEEYMDWLHQSPVPKLLFWGTPGVLIP
PAEAARLAKSLPNCKAVDIGPGLNLLQEDNPDLIGSEIARWLSTLEISG

HaloTag, eNOS-peptide, Linker, Calmodulin, HaloTag
Rationally placed tryptophan: G171W
Hits from directed evolution screen: G176A, V178I, P180T

WHaloCaMP1b

MAEIGTGFPFDPHYVEVLGERMHYVDVGPRDGTPVLFLHGNPTSSYVWRNIIPHVAPTHRCIAPDLIGMGK
SDKPDLGYFFDDHVRFMDAFIEALGLEEVVLVIHDWGSALGFHWAKRNPERVKGIAFMEFIRPIPTWDEWP
EFARETFQWFRTARRKWQKTGHAVRAIGRLSSGGSGGSGGSGGSDQLTEEQIAEFKEAFSLFDKDGDGTITT
KELGTVMRSLGQNPTEAELQDMINEVDADGNGTIDFPEFLTMMARKMKDTDSEEEIREAFRVFDKDGNGYI
SAAELRHVMTNLGEKLTDEEVDEMIREADIDGDGQVNYEEFVQMMTAKVDRKLIIDQNVFIEGTLPMGVVR
PLTEVEMDHYREPFLNPVDREPLWRFPNELPIAGEPANIVALVEEYMDWLHQSPVPKLLFWGTPGVLIPPAE
AARLAKSLPNCKAVDIGPGLNLLQEDNPDLIGSEIARWLSTLEISG

HaloTag, MLCK-peptide, Linker, Calmodulin, HaloTag
Rationally placed tryptophan: A151W
Hits from directed evolution screen: G158D

Supplementary Note 1

The $K_{L\text{–}Z}$

Standard rhodamines that have an *ortho*-carboxyl group on the pendant phenyl ring can exist in two forms in equilibrium (**Fig. SN. 1a**). The lactone form (**L**) occurs when the carboxyl group forms a bond with the central carbon atom in an intramolecular reaction, forming a lactone. In this uncharged structure, the two aryl groups are independent, yielding a colorless and nonfluorescent molecule. This is in equilibrium with the zwtterionic form (**Z**), where the lactone bond is broken, yielding a positively charged xanthylium or xanthylium-like species and a carboxylate moiety. The extended conjugated system in the zwitterionic form gives rise to the strong visible absorption and fluorescence properties that are characteristic of rhodamine dyes.

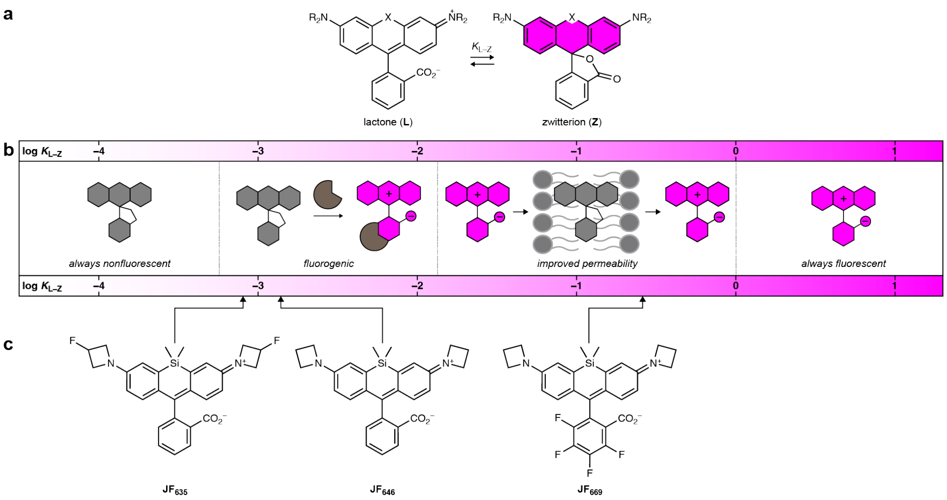

Figure Supplementary Note (SN) 1. The lactone–zwitterion equilibrium constant ($K_{L\text{–}Z}$) predicts performance of rhodamine dyes in biological systems. (**a**). General rhodamine structure showing the equilibrium between the lipophilic colorless lactone (**L**) and fluorescent zwitterion (**Z**). (**b**) Framework relating the $K_{L\text{–}Z}$ to performance of rhodamine dyes. (**c**) Structures of Si-rhodamine dyes **JF$_{635}$**, **JF$_{646}$**, and **JF$_{669}$**.

The dynamic equilibrium between the lactone and zwitterionic forms of rhodamine dyes is dependent on both the environment around the dye and the chemical structure of the fluorophore. Differences in the lactone–zwitterion equilibrium can be measured in various ways, for example by dioxane-water titrations used in various works,[9-13] or by measuring the $K_{L\text{-}Z}$[14-16].

$K_{L\text{–}Z}$ is calculated using equation 1:

$$K_{L-Z} = \frac{\varepsilon_{dw}/\varepsilon_{max}}{1-\varepsilon_{dw}/\varepsilon_{max}}$$

Where is ε_{dw} is the molar extinction coefficient when the dye is in a 1:1 (v/v) dioxane:water mixture. ε_{max} is the maximum molar extinction coefficient measured in either 0.1% (v/v)

trifluoroacetic acid (TFA) in ethanol or 0.1% (v/v) TFA in 2,2,2-trifluoroethanol (TFE) depending on dye type. These strongly acidic conditions presumably protonate the *ortho*-carboxyl group; the resulting cationic species is a proxy for the zwitterionic form allowing estimation of the absorptivity of pure zwitterion and subsequent calculation of $K_{L–Z}$.

We previously developed a general framework correlating $K_{L–Z}$ with the performance of dyes in biological environments[14-16] (**Fig. SN. 1b**). We found that both fluorogenicity—dyes that turn on when they bind their cognate biomolecular target—and bioavailability strongly depend on $K_{L–Z}$. HaloTag ligands based on dyes with $4\times10^{-4} < K_{L–Z} < 9\times10^{-3}$ are typically highly fluorogenic. Dyes with $9\times10^{-3} < K_{L–Z} < 1$ usually show high cell and tissue permeability. An example is the Si-rhodamine series **JF$_{646}$**, **JF$_{635}$**, and **JF$_{669}$** (**Fig. SN. 1c**). **JF$_{646}$** exhibits a $K_{L–Z} = 0.0014$, which puts it squarely in the fluorogenic region (**Fig. SN. 1b**); **JF$_{646}$-HaloTag ligand** shows modest fluorogenicity with a ~20-fold increase in absorption and fluorescence intensity upon binding the HaloTag protein.[13] Addition of a single fluorine atom on each azetidine auxochrome to give **JF$_{635}$** substantially decreases $K_{L–Z} = 6\times10^{-4}$; this leads to a higher degree of fluorogenicity for the **JF$_{635}$-HaloTag ligand** (~200-fold).[14] Installation of fluorine atoms on the pendant phenyl ring to give **JF$_{669}$** increases the $K_{L–Z} = 0.262$; the **JF$_{669}$-HaloTag ligand** shows only modest fluorogenicity but high bioavailability with the ability to cross the blood–brain barrier after *intra venous* administration.[15] This rubric is also applicable for standard oxygen-containing rhodamines and carborhodamines.[15] The example of **JF$_{646}$**, **JF$_{635}$**, and **JF$_{669}$** demonstrates the challenge of creating a fluorophore that is both fluorogenic and highly bioavailable, since both attributes depend on a single property.

The $K_{L–Z}$ parameter is not perfect and we note several criticisms. First, the measurement is done in a mixture of dioxane and water. Although this gives a wide range of values that show clear trends with bioavailability and fluorogenicity, these conditions are not biologically relevant; it is difficult to relate the measured $K_{L–Z}$ to the dye state in a biological environment. Second, we measure the $K_{L–Z}$ of parent dyes, and not conjugated to functional handles for example the chloroalkane in the HaloTag Ligand. Functional handles can affect this equilibrium, making this parameter useful in comparing compounds within a specific class (*e.g.*, HaloTag ligands) but not across different compound types. Third, other properties affect cell-permeability. For example, the phosphine oxide dye **JF$_{722}$** exhibits a $K_{L–Z} = 0.026$, which should indicate high bioavailability for HaloTag ligand derivatives.[15] The **JF$_{722}$-HaloTag ligand** does not show efficient labeling in animals models, however, presumably due to the additional lipophilic phenyl group on the fluorophore structure.

In summary, the $K_{L–Z}$ is a useful predictor of the performance of dye conjugates in biological environments. The relationship between $K_{L–Z}$ and bioavailability and fluorogenicity provides a straightforward framework for optimization of dye ligands within a structural class[15]. For chemigenetic sensors, however, the dependence of both fluorogenicity and bioavailability on a single property is problematic. Previous sensors, such as HaloCaMP,[2] relied on fluorogenic dye ligands derived from fluorophores with relatively low $K_{L–Z}$ values. Although these dyes show robust fluorescence changes in response to changes in the environment, they did not exhibit good bioavailability in the central nervous system in animal models, limiting the ability to allow functional imaging in the central nervous system with single cell resolution. In this work, we focus instead on building chemigenetic Ca^{2+} sensors around dye ligands where the dye has higher K_{L-Z}

values, such as **JF$_{669}$**. As **JF$_{669}$-HaloTag ligand** shows only modest fluorogenicity we needed to explore a different way of modulating the fluorescence emission – here we use a strategically placed tryptophan, which can quench the fluorescence of the dye in a Ca^{2+} dependent manner.

Supplementary References:

1. Encell, L.P. et al. Development of a dehalogenase-based protein fusion tag capable of rapid, selective and covalent attachment to customizable ligands. *Curr Chem Genomics* **6**, 55-71 (2012).
2. Deo, C. et al. The HaloTag as a general scaffold for far-red tunable chemigenetic indicators. *Nature Chemical Biology* **17**, 718-723 (2021).
3. Zhang, Y. et al. Fast and sensitive GCaMP calcium indicators for imaging neural populations. *Nature* **615**, 884-891 (2023).
4. Hashizume, R. et al. A genetically encoded far-red fluorescent calcium ion biosensor derived from a biliverdin-binding protein. *Protein Science* **31**, e4440 (2022).
5. Subach, O.M., Barykina, N.V., Anokhin, K.V., Piatkevich, K.D. & Subach, F.V. Near-Infrared Genetically Encoded Positive Calcium Indicator Based on GAF-FP Bacterial Phytochrome. *International Journal of Molecular Sciences* **20**, 3488 (2019).
6. Subach, O.M. & Subach, F.V. GAF-CaMP3–sfGFP, An Enhanced Version of the Near-Infrared Genetically Encoded Positive Phytochrome-Based Calcium Indicator for the Visualization of Neuronal Activity. *International Journal of Molecular Sciences* **21**, 6883 (2020).
7. Shemetov, A.A. et al. A near-infrared genetically encoded calcium indicator for in vivo imaging. *Nature Biotechnology* **39**, 368-377 (2021).
8. Matlashov, M.E., Vera, J., Kasatkina, L.A., Khodakhah, K. & Verkhusha, V.V. Design and Initial Characterization of a Small Near-Infrared Fluorescent Calcium Indicator. *Frontiers in Cell and Developmental Biology* **10** (2022).
9. Ioffe, I. & Otten, V. Rhodamine dyes and related compounds. XIV. Mutual conversions of colorless and colored forms of rhodamine and rhodol. *Zh. Obshch. Khim* **1**, 343-346 (1965).
10. Watkins, R.W., Lavis, L.D., Kung, V.M., Los, G.V. & Raines, R.T. Fluorogenic affinity label for the facile, rapid imaging of proteins in live cells. *Organic & Biomolecular Chemistry* **7**, 3969-3975 (2009).
11. Lukinavičius, G. et al. A near-infrared fluorophore for live-cell super-resolution microscopy of cellular proteins. *Nature Chemistry* **5**, 132-139 (2013).
12. Butkevich, A.N. et al. Fluorescent Rhodamines and Fluorogenic Carbopyronines for Super-Resolution STED Microscopy in Living Cells. *Angew Chem Int Edit* **55**, 3290-3294 (2016).
13. Grimm, J.B. et al. A general method to improve fluorophores for live-cell and single-molecule microscopy. *Nature Methods* **12**, 244-250 (2015).
14. Grimm, J.B. et al. A general method to fine-tune fluorophores for live-cell and in vivo imaging. *Nature Methods* **14**, 987-994 (2017).

15. Grimm, J.B. et al. A general method to optimize and functionalize red-shifted rhodamine dyes. *Nature Methods* **17**, 815-821 (2020).
16. Zheng, Q. et al. Rational Design of Fluorogenic and Spontaneously Blinking Labels for Super-Resolution Imaging. *ACS Central Science* **5**, 1602-1613 (2019).
17. Sun, W.-C., Gee, K.R., Klaubert, D.H. & Haugland, R.P. Synthesis of Fluorinated Fluoresceins. *The Journal of Organic Chemistry* **62**, 6469-6475 (1997).

Supplementary Note 2

General Experimental Information for Synthesis

Commercial reagents were obtained from reputable suppliers and used as received. All solvents were purchased in septum-sealed bottles stored under an inert atmosphere. All reactions were sealed with septa through which a nitrogen atmosphere was introduced unless otherwise noted. Reactions were conducted in round-bottomed flasks or septum-capped crimp-top vials containing Teflon-coated magnetic stir bars. Heating of reactions was accomplished with a silicon oil bath or an aluminum reaction block on top of a stirring hotplate equipped with an electronic contact thermometer to maintain the indicated temperatures.

Reactions were monitored by thin layer chromatography (TLC) on precoated TLC glass plates (silica gel 60 F_{254}, 250 µm thickness) or by LC/MS (Phenomenex Kinetex 2.1 mm × 30 mm 2.6 µm C18 column; 5 µL injection; 5–98% $MeCN/H_2O$, linear gradient, with constant 0.1% v/v HCO_2H additive; 6 min run; 0.5 mL/min flow; ESI; positive ion mode). TLC chromatograms were visualized by UV illumination or developed with *p*-anisaldehyde, ceric ammonium molybdate, or $KMnO_4$ stain. Reaction products were purified by flash chromatography on an automated purification system using pre-packed silica gel columns or by preparative HPLC (Phenomenex Gemini–NX 30 × 150 mm 5 µm C18 column). Analytical HPLC analysis was performed with an Agilent Eclipse XDB 4.6 × 150 mm 5 µm C18 column under the indicated conditions. High-resolution mass spectrometry was performed by the High Resolution Mass Spectrometry Facility at the University of Iowa.

NMR spectra were recorded on a 400 MHz spectrometer. ^{1}H and ^{13}C chemical shifts were referenced to TMS or residual solvent peaks, and ^{19}F chemical shifts were referenced to $CFCl_3$. Data for ^{1}H NMR spectra are reported as follows: chemical shift (δ ppm), multiplicity (s = singlet, d = doublet, t = triplet, q = quartet, dd = doublet of doublets, m = multiplet), coupling constant (Hz), integration. Data for ^{13}C NMR spectra are reported by chemical shift (δ ppm) with hydrogen multiplicity (C, CH, CH_2, CH_3) information obtained from DEPT spectra.

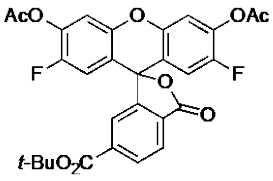

6-*tert*-Butoxycarbonyl Oregon Green diacetate (1): 6-Carboxy Oregon Green diacetate[17] (808 mg, 1.63 mmol) was taken up in CH_2Cl_2 (23 mL) under nitrogen, and *tert*-butyl *N*,*N'*-diisopropylcarbamimidate (3.26 g, 16.3 mmol, 10 eq) was added. After stirring the reaction at room temperature for 24 h, the resulting white suspension was filtered through a 2.5g celite cartridge. Purification by flash chromatography (0–30% EtOAc/hexanes, linear gradient, with constant 20% v/v CH_2Cl_2 additive) yielded 538 mg (60%) of **1** as an off white solid. ^{1}H NMR ($CDCl_3$, 400 MHz) δ 8.30 (dd, J = 8.0, 1.3 Hz, 1H), 8.09 (d, J = 8.0 Hz, 1H), 7.76 (d, J = 1.4 Hz, 1H), 7.16 (d, $^4J_{HF}$ = 6.3 Hz, 2H), 6.56 (d, $^3J_{HF}$ = 9.8 Hz, 2H), 2.36 (s, 6H), 1.59 (s, 9H); ^{19}F NMR ($CDCl_3$, 376 MHz) δ -131.90 (dd, J_{FH} = 9.9, 6.5 Hz); ^{13}C NMR ($CDCl_3$, 101 MHz) δ 167.9 (C), 167.7 (C), 163.8 (C), 151.9 (C), 150.7 (d, $^1J_{CF}$ = 247.8 Hz, CF), 147.2 (d, $^4J_{CF}$ = 2.3 Hz, C), 140.4 (d, $^2J_{CF}$ = 14.9 Hz, C), 139.1 (C), 131.9 (CH), 128.9 (C), 125.6 (CH), 125.0 (CH), 115.9 (d, $^3J_{CF}$ = 6.2 Hz, C), 114.9 (d, $^2J_{CF}$ = 21.6 Hz, CH), 112.9 (CH), 83.2 (C), 81.5 (C), 28.2 (CH_3), 20.6 (CH_3); HRMS (ESI) calcd for $C_{29}H_{23}F_2O_9$ [M+H]$^+$ 553.1305, found 553.1301.

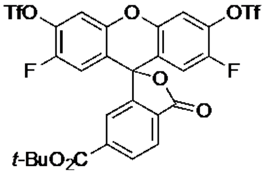

6-*tert*-Butoxycarbonyl Oregon Green ditriflate (2): To a solution of 6-*tert*-butoxycarbonyl Oregon Green diacetate (**1**; 1.27 g, 2.30 mmol) in 1:1 THF/MeOH (30 mL) was added 1 M NaOH (5.53 mL, 5.53 mmol, 2.4 eq). The reaction was stirred at room temperature for 1 h. The resulting red-orange solution was acidified with 1 N HCl (7.5 mL), diluted with water, and extracted with EtOAc (2×). The organics were washed with brine, dried over anhydrous $MgSO_4$, filtered, and concentrated *in vacuo* to provide a red solid. The crude solid was suspended in CH_2Cl_2 (15 mL) and cooled to 0 °C. Pyridine (1.49 mL, 18.4 mmol, 8 eq) and trifluoromethanesulfonic anhydride (1.55 mL, 9.21 mmol, 4 eq) were added, and the ice bath was removed. The reaction was stirred at room temperature for 1 h. It was subsequently diluted with water and extracted with CH_2Cl_2

(2×). The combined organic extracts were dried over anhydrous $MgSO_4$, filtered, and evaporated. Silica gel chromatography (0–30% EtOAc/hexanes, linear gradient) yielded 1.24 mg (74%) of **2** as a white solid. ^{1}H NMR ($CDCl_3$, 400 MHz) δ 8.32 (dd, *J* = 8.0, 1.3 Hz, 1H), 8.14 (d, *J* = 8.0 Hz, 1H), 7.77 (d, *J* = 1.3 Hz, 1H), 7.39 (d, $^4J_{HF}$ = 6.1 Hz, 2H), 6.73 (d, $^3J_{HF}$ = 9.3 Hz, 2H), 1.59 (s, 9H); ^{19}F NMR ($CDCl_3$, 376 MHz) δ -73.31 (d, *J* = 4.7 Hz, 6F), -130.67 (dp, *J* = 9.9, 5.0 Hz, 2F); ^{13}C NMR ($CDCl_3$, 101 MHz) δ 167.2 (C), 163.6 (C), 151.4 (C), 150.4 (d, $^1J_{CF}$ = 252.4 Hz, CF), 146.8 (d, $^4J_{CF}$ = 2.5 Hz, C), 139.6 (C), 138.3 (d, $^2J_{CF}$ = 15.3 Hz, C), 132.4 (CH), 128.2 (C), 126.2 (CH), 124.8 (CH), 118.9 (d, $^3J_{CF}$ = 5.8 Hz, C), 118.8 (q, $^1J_{CF}$ = 321.0 Hz, CF_3), 116.0 (d, $^2J_{CF}$ = 21.2 Hz, CH), 113.3 (C), 83.5 (C), 80.0 (C), 28.2 (CH_3); HRMS (ESI) calcd for $C_{27}H_{17}F_8O_{11}S_2$ $[M+H]^+$ 733.0079, found 733.0076.

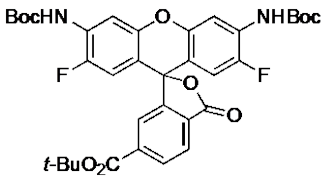

6-*tert*-Butoxycarbonyl-JF$_{494}$ bis(*tert*-butyl carbamate) (3): A vial was charged with 6-*tert*-butoxycarbonyl Oregon Green ditriflate (**2**; 150 mg, 0.204 mmol), *tert*-butyl carbamate (57.6 mg, 0.491 mmol, 2.4 eq), Pd_2dba_3 (18.6 mg, 0.0204 mmol, 0.1 eq), Xantphos (35.5 mg, 0.0610 mmol, 0.3 eq), and Cs_2CO_3 (186.8 mg, 0.573 mmol, 2.8 eq). The vial was sealed and evacuated/backfilled with nitrogen (3×). Dioxane (3 mL) was added; after flushing the reaction again with nitrogen (3×), it was stirred at 100 °C for 3 h. It was then cooled to room temperature, diluted with MeOH, deposited onto Celite, and concentrated *in vacuo*. The solid was purified by silica gel chromatography (0–20% EtOAc/hexanes with constant 20% CH_2Cl_2 additive, linear gradient; dry load with Celite) to afford **3** (97.1 mg, 71%) as a white solid. ^{1}H NMR ($CDCl_3$, 400 MHz) δ 8.25 (dd, *J* = 8.0, 1.3 Hz, 1H), 8.11 (d, $^4J_{HF}$ = 6.3 Hz, 2H), 8.06 (dd, *J* = 8.0, 0.8 Hz, 1H), 7.69 – 7.64 (m, 1H), 6.77 (d, $^4J_{HF}$ = 3.5 Hz, 2H), 6.41 (d, $^3J_{HF}$ = 11.1 Hz, 2H), 1.56 (s, 9H), 1.55 (s, 18H); ^{19}F NMR ($CDCl_3$, 376 MHz) δ -137.40 – -138.04 (m); ^{13}C NMR ($CDCl_3$, 101 MHz) δ 168.2 (C), 164.0 (C), 152.7 (C), 152.0 (C), 148.12 (d, $^1J_{CF}$ = 240.0 Hz, CF), 148.08 (d, $^4J_{CF}$ = 1.8 Hz, C), 138.9 (C), 131.5 (CH), 129.8 (d, $^2J_{CF}$ = 11.9 Hz, C), 129.2 (C), 125.3 (CH), 124.9 (CH), 112.6 (d, $^2J_{CF}$ = 22.0 Hz, CH), 110.6 (d, $^3J_{CF}$ = 6.7 Hz, C), 108.0 (CH), 82.92 (C), 82.87 (C), 82.0 (C), 28.4 (CH_3), 28.2 (CH_3); HRMS (ESI) calcd for $C_{35}H_{37}F_2N_2O_9$ $[M+H]^+$ 667.2462, found 667.2462.

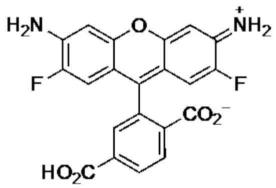

6-Carboxy-JF$_{494}$ (4): 6-*tert*-Butoxycarbonyl-JF$_{494}$ bis(*tert*-butyl carbamate) (**3**; 100 mg, 0.150 mmol) was taken up in CH_2Cl_2 (3 mL), and trifluoroacetic acid (0.6 mL) was added. The reaction was stirred at room temperature for 4 h. Toluene (3 mL) was added; the reaction mixture was concentrated to dryness and then azeotroped with MeOH three times to provide **4** as a metallic red-pink solid (70.5 mg, 90%, TFA salt). Analytical HPLC and NMR indicated that the material was >95% pure and did not require further purification prior to amide coupling. ^{1}H NMR (CD_3OD with 1% TFA, 400 MHz) δ 8.46 (dd, J = 8.1, 0.7 Hz, 1H), 8.42 (dd, J = 8.2, 1.6 Hz, 1H), 8.00 (dd, J = 1.5, 0.7 Hz, 1H), 7.07 (d, $^{4}J_{HF}$ = 7.0 Hz, 2H), 6.90 (d, $^{3}J_{HF}$ = 11.1 Hz, 2H); ^{19}F NMR (CD_3OD with 1% TFA, 376 MHz) δ -130.55 (dd, J_{FH} = 11.0, 7.0 Hz); Analytical HPLC: 98.9% purity (10–95% MeCN/H_2O, linear gradient, with constant 0.1% v/v TFA additive; 20 min run; 1 mL/min flow; ESI; positive ion mode; detection at 500 nm); HRMS (ESI) calcd for $C_{21}H_{13}F_2N_2O_5$ $[M+H]^+$ 411.0787, found 411.0782.

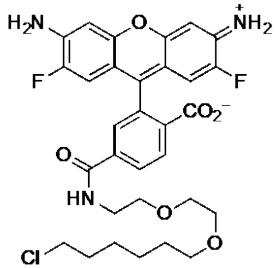

JF$_{494}$–HaloTag ligand (5): 6-Carboxy-JF$_{494}$ (**4**; 30 mg, 0.0572 mmol) was combined with DSC (36.6 mg, 0.143 mmol, 2.5 eq) in DMF (2 mL). After adding Et_3N (47.8 µL, 0.343 mmol, 6 eq) and DMAP (0.699 mg, 5.72 µmol, 0.1 eq), the reaction was stirred at room temperature for 1 h. A solution of 2-(2-((6-chlorohexyl)oxy)ethoxy)ethanamine ("HaloTag(O2)amine"; TFA salt; 57.9 mg, 0.172 mmol, 3 eq) in DMF (500 µL) was then added. The reaction was stirred an additional 1 h at room temperature. Purification of the crude reaction mixture by reverse phase HPLC (20–60% MeCN/H_2O, linear gradient, with constant 0.1% v/v TFA additive) afforded 20.9 mg (50%, TFA

salt) of **5** as a dark red fluffy solid. ^{1}H NMR (CD_3OD, 400 MHz) δ 8.79 (t, *J* = 5.5 Hz, 1H), 8.39 (d, *J* = 8.2 Hz, 1H), 8.21 (dd, *J* = 8.2, 1.7 Hz, 1H), 7.78 (d, *J* = 1.7 Hz, 1H), 7.04 (d, $^4J_{HF}$ = 7.0 Hz, 2H), 6.88 (d, $^3J_{HF}$ = 11.2 Hz, 2H), 3.70 – 3.55 (m, 8H), 3.52 (t, *J* = 6.6 Hz, 2H), 3.43 (t, *J* = 6.5 Hz, 2H), 1.76 – 1.67 (m, 2H), 1.54 – 1.45 (m, 2H), 1.43 – 1.28 (m, 4H); ^{19}F NMR (CD_3OD, 376 MHz) δ -130.59 (dd, J_{FH} = 11.1, 7.0 Hz); Analytical HPLC: 98.6% purity (10–95% MeCN/H_2O, linear gradient, with constant 0.1% v/v TFA additive; 20 min run; 1 mL/min flow; ESI; positive ion mode; detection at 500 nm); HRMS (ESI) calcd for $C_{31}H_{33}ClF_2N_3O_6$ $[M+H]^+$ 616.2020, found 616.2019.
