# Supplementary material for: A modular chemigenetic calcium indicator for multiplexed in vivo functional imaging
Source: Nat Methods. 2024 Sep 20;21(10):1916–25. doi: 10.1038/s41592-024-02411-6 (PMC11466818; doi:10.1038/s41592-024-02411-6)
Supplement: Supplementary file 2 — Reporting Summary [file 41592_2024_2411_MOESM2_ESM.pdf]

nature portfolio

Corresponding author(s): Eric Schreiter and Helen Farrants

Last updated by author(s): 06/03/2024

Reporting Summary

Nature Portfolio wishes to improve the reproducibility of the work that we publish. This form provides structure for consistency and transparency in reporting. For further information on Nature Portfolio policies, see our Editorial Policies and the Editorial Policy Checklist.

Statistics

For all statistical analyses, confirm that the following items are present in the figure legend, table legend, main text, or Methods section.

n/a	Confirmed	
☐	☒	The exact sample size (*n*) for each experimental group/condition, given as a discrete number and unit of measurement
☐	☒	A statement on whether measurements were taken from distinct samples or whether the same sample was measured repeatedly
☐	☒	The statistical test(s) used AND whether they are one- or two-sided<br>*Only common tests should be described solely by name; describe more complex techniques in the Methods section.*
☒	☐	A description of all covariates tested
☒	☐	A description of any assumptions or corrections, such as tests of normality and adjustment for multiple comparisons
☐	☒	A full description of the statistical parameters including central tendency (e.g. means) or other basic estimates (e.g. regression coefficient) AND variation (e.g. standard deviation) or associated estimates of uncertainty (e.g. confidence intervals)
☐	☒	For null hypothesis testing, the test statistic (e.g. *F*, *t*, *r*) with confidence intervals, effect sizes, degrees of freedom and *P* value noted<br>*Give P values as exact values whenever suitable.*
☒	☐	For Bayesian analysis, information on the choice of priors and Markov chain Monte Carlo settings
☒	☐	For hierarchical and complex designs, identification of the appropriate level for tests and full reporting of outcomes
☒	☐	Estimates of effect sizes (e.g. Cohen's *d*, Pearson's *r*), indicating how they were calculated

Our web collection on statistics for biologists contains articles on many of the points above.

Software and code

Policy information about availability of computer code

Data collection	The Cary Model 100 spectrometer was controlled by CaryWinUV 4.20(468) software. The Cary Eclipse was controlled by Cary Eclipse Scan Application 1.1(132) software. The Quantaurus Quantum Yield spectrophotometer was controlled by PLQY software U6039-05 3.4.2. The Zeiss Lightsheet Z.1 was controlled by Zen. The Leica STELLARIS 8 FALCON microscope was controlled by Leica Application Suite X. The Nikon Eclipse Ti microscope was controlled by NIS-Elements AR 4.40.0, 64-bit (build 1084) software. Scanimage was implemented in MATLAB R2020b for image acquisitions. Wavesurfer Version 1.0.0.0 was also used implemented via MATLAB R2020b. µManager Version 1.4. The plate reader was run via Tecan Sparkcontrol method editor v. 3.0.
Data analysis	Data analysis was performed in Microsoft Excel Version 16.85 and MATLAB 2012b and R2023B. Snapgene v. 7.2.1. was used to design constructs. Image analysis was performed in FIJI v. 2.14.0/1.54f and Leica LASX FLIM/FCS software. GraphPad Prism software v. 10.2.3 was used for data representation, curve fitting and statistical tests. Neuronal activity was analyzed using suite2p, Cellpose and Rastermap. The protein-dye structure was solved in the CCP4 suite, using Xia2/DIALS, scala, PHASER, REFMAC5 and Coot.

For manuscripts utilizing custom algorithms or software that are central to the research but not yet described in published literature, software must be made available to editors and reviewers. We strongly encourage code deposition in a community repository (e.g. GitHub). See the Nature Portfolio guidelines for submitting code & software for further information.

Data

Policy information about availability of data

All manuscripts must include a data availability statement. This statement should provide the following information, where applicable:

- Accession codes, unique identifiers, or web links for publicly available datasets
- A description of any restrictions on data availability
- For clinical datasets or third party data, please ensure that the statement adheres to our policy

The authors declare that the source data used to generate the figures in this manuscript are available from the Figshare repository: https://doi.org/10.25378/janelia.25934782 . The structure of HaloTag7 bound to JF669 has been deposited to the Protein Data Bank (PDBID = 8SW8).

DNA plasmids encoding WHaloCaMP used in this work are available from Addgene as follows:
205303 pRSET-WHaloCaMP1a-EGFP
205304 pRSET-WHaloCaMP1a
205305 pRSET-WHaloCaMP1b-EGFP
205306 pRSET-WHaloCaMP-eNOSpep-EGFP
205307 pAAV-synapsin-WHaloCaMP1a-EGFP
205308 pAAV-synapsin-WHaloCaMP1a
205309 pAAV-CaMKII-WHaloCaMP1a-EGFP
205310 pAAV-CaMKII-WHaloCaMP1a
205311 pTol2-elavl3-WHaloCaMP1a-EGFP
205312 pTol2-elavl3-WHaloCaMP1a

Human research participants

Policy information about studies involving human research participants and Sex and Gender in Research.

Reporting on sex and gender	N/A
Population characteristics	N/A
Recruitment	N/A
Ethics oversight	N/A

Note that full information on the approval of the study protocol must also be provided in the manuscript.

Field-specific reporting

Please select the one below that is the best fit for your research. If you are not sure, read the appropriate sections before making your selection.

☒ Life sciences ☐ Behavioural & social sciences ☐ Ecological, evolutionary & environmental sciences

For a reference copy of the document with all sections, see nature.com/documents/nr-reporting-summary-flat.pdf

Life sciences study design

All studies must disclose on these points even when the disclosure is negative.

Sample size	No specific sample size calculations were performed. Generally, sample size was the maximum possible within reasonable time and resource application limits.
Data exclusions	No data was excluded from analysis.
Replication	All attempts at replication were successful. Number of replications for each experiment are described in the methods, figure captions and/or main text.
Randomization	Where appropriate for comparative analysis, samples were randomly allocated into experimental groups.
Blinding	No blinding was performed, primarily to reduce experimental complexity as no novel comparative experiments were central to the conclusions of this work.

Reporting for specific materials, systems and methods

We require information from authors about some types of materials, experimental systems and methods used in many studies. Here, indicate whether each material, system or method listed is relevant to your study. If you are not sure if a list item applies to your research, read the appropriate section before selecting a response.

Materials & experimental systems

n/a	Involved in the study
☒	☐ Antibodies
☐	☒ Eukaryotic cell lines
☒	☐ Palaeontology and archaeology
☐	☒ Animals and other organisms
☒	☐ Clinical data
☒	☐ Dual use research of concern

Methods

n/a	Involved in the study
☒	☐ ChIP-seq
☒	☐ Flow cytometry
☒	☐ MRI-based neuroimaging

Eukaryotic cell lines

Policy information about cell lines and Sex and Gender in Research

Cell line source(s)	HeLa cells (CCL-2) were purchased from ATCC.
Authentication	No authentication of the cell line was performed.
Mycoplasma contamination	Cells were regularly tested by Janelia Cell Culture Facility and were negative.
Commonly misidentified lines (See ICLAC register)	No commonly misidentified cell lines were used in this study.

Animals and other research organisms

Policy information about studies involving animals; ARRIVE guidelines recommended for reporting animal research, and Sex and Gender in Research

Laboratory animals	Adult C57/BL6 male mice, 2-4 months old. Emx1IRES cre mice (JAX #005628 strain) and FLIM-AKAR mice (JAX #039003), both C57/BL6 background, P15-P19. Rattus norvegicus (rat), Sprague-Dawley, 0-1 days. Zebra fish larvae was of the species Danio rerio and were transgenic and used at 4-6. d.p.f. Flies were of the species Drosophila melanogaster and used 2-10 days progeny of a heterozygous cross of drivers and WHaloCaMP variants.
Wild animals	No wild animals were used in this study.
Reporting on sex	The gender of zebrafish larvae cannot be determined, thus sex was not considered in the experimental design. Flies (adult, females) were used. Male mice were used for imaging WHaloCaMP in the visual cortex. For the slice experiments in mice, the sex of the mice was not determined.
Field-collected samples	No field-collected samples were used.
Ethics oversight	Experimental protocols were conducted according to the National Institutes of Health guidelines for animal research and were approved by the Institutional Animal Care and Use Committee at the Janelia Research Campus, HHMI, Institutional Animal Care and Use Committee at University of Maryland Baltimore, and Washington University Institutional Animal Care and Use Committee.

Note that full information on the approval of the study protocol must also be provided in the manuscript.
